# Are Noncovalent C─H⋯ Au Bonds Comparable to C─H⋯ π Bonds? A Theoretical Perspective

**DOI:** 10.1002/asia.202500736

**Published:** 2025-07-24

**Authors:** Sergi Burguera, Antonio Bauzá

**Affiliations:** ^1^ Departament de Química Universitat de les Illes Balears Ctra. de Valldemossa km 7.5 Palma de Mallorca 07122 Spain

**Keywords:** C─H···Au and C─H···π interactions, DFT‐D3 study, EDA study, QTAIM and NCIplot analyses, Supramolecular chemistry

## Abstract

Herein, we have theoretically studied and compared the physical nature of C─H···Au and C─H···π bonds at the PBE0‐D3/def2‐TZVP level of theory. To achieve this, we have used a series of alkanes exhibiting i) linear, ii) cyclic, and iii) branched geometries and Au and C layers, mimicking a decahedral Au nanoparticle's face and a graphene surface portion, respectively. The calculated supramolecular complexes were compared in terms of their geometry and stability, unveiling interesting trends regarding the i) length, ii) shape, and iii) substitution degree of the aliphatic chain. In addition, they were characterized using the Quantum Theory of Atoms in Molecules (QTAIM) and Non‐Covalent Interaction plot (NCIplot) methodologies, which shed light on the aliphatic C─H bond distribution along the complexes as well as the extension in real space of the interactions. Lastly, the Energy Decomposition Analysis (EDA) technique was useful to unveil resemblances/differences in the energetic contributors of both supramolecular forces. We believe that this conceptual study will be of interest to both supramolecular and materials science scientists, owing to the implications of these noncovalent interactions in molecular recognition, catalysis, and solid‐state chemistry.

## Graphical abstract

In this study, the geometry and stability features as well as the physical nature of C─H···Au and C─H···π noncovalent complexes have been analyzed and compared at the PBE0‐D3/def2‐TZVP level of theory involving a series of alkanes and i) graphene and ii) an Au layer.

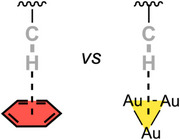

## 1 Introduction

In recent years, nanotechnology has established new frontiers in science and engineering, leading to crucial advances in materials science,^[^ ^1^ ^]^ chemistry,^[^ ^2^ ^]^ biology,^[^ ^3^ ^]^ and environmental sciences.^[^ ^4^ ^]^ In this context, gold nanoparticles (AuNPs) and graphene stand out as nanomaterials of considerable interest, owing to their extraordinary physicochemical properties.^[^ ^5^, ^6^ ^]^ These nanostructures exhibit highly tunable surfaces, significant catalytic activity, excellent electronic features, and remarkable surface‐to‐volume ratios, making them ideal frameworks for studying and facilitating interactions involving organic compounds^[^ ^7^, ^8^ ^]^ with high efficiency and specificity. This is possible because of their electronic structure, which regulates the adsorption of reaction substrates or other organic compounds of interest on the AuNP and graphene surfaces. Through molecular mechanisms that encompass metal coordination, electrostatics, and van der Waals (vdW) interactions (including π–π stacking and C─H···Au/C bonds), these materials exhibit promising features in supramolecular chemistry,^[^ ^9^ ^]^ catalysis,^[^ ^10^ ^]^ environmental remediation^[^ ^11^ ^]^ as well as in biomedicine fields.^[^ ^12^ ^]^

For instance, in the case of AuNPs, one of the primary modes of interaction is surface adsorption, which is facilitated by the large surface area and high surface energy of the particles.^[^ ^13^, ^14^ ^]^ Thus, organic molecules containing functional groups such as thiols, amines, and carboxylates can form stable covalent or coordinate bonds with the Au surface.^[^ ^15^ ^]^ This can be related to the nanoparticle's catalytic activity, particularly at their surface atoms, which are often undercoordinated and thus highly reactive.^[^ ^16^, ^17^ ^]^ In this regard, several theoretical studies have pointed out that these polar groups can noncovalently interact with the AuNP surface through the formation of Regium bonding interactions (an attractive noncovalent force involving elements of group 11 acting as electrophiles and electron‐rich species).^[^ ^18^, ^19^, ^20^, ^21^ ^]^ These supramolecular bonds are mainly based on the presence of low electron density sites exhibiting electron‐rich (lumps) and electron‐poor (holes) properties over the metal nanoparticle surface, which facilitate its engagement in weak binding events. Additionally, vdW forces and hydrophobic interactions can also contribute to the adsorption of non‐polar organic molecules onto the nanoparticle surface,^[^ ^22^, ^23^ ^]^ such as C─H···Au and Regium–π interactions, which involve either aliphatic or aromatic carbon‐based moieties and noble metal nanoparticles.^[^ ^24^, ^25^, ^26^ ^]^

On the other hand, graphene and its derivatives interact with aromatic compounds predominantly through π–π stacking interactions.^[^ ^27^ ^]^ This type of noncovalent bonding is particularly strong with polycyclic aromatic hydrocarbons, dyes, and many biologically active molecules.^[^ ^28^ ^]^ Despite this, aliphatic molecules are also able to interact with pristine graphene through the establishment of dispersion‐based interactions, such as C─H··· π bonds.^[^ ^29^ ^]^ This type of interaction becomes especially significant in long‐chain aliphatic compounds, such as fatty acids and hydrocarbons (e.g., hexane, octane, dodecane), which can maximize surface contact with graphene by increasing the adsorption strength due to greater surface contact and cumulative weak forces.^[^ ^30^, ^31^ ^]^ Furthermore, studies have shown that aliphatic molecules can influence the wetting behavior of graphene surfaces and even form self‐assembled monolayers through the establishment of vdW interactions, thus affecting graphene's electronic and optical properties.^[^ ^32^ ^]^ Lastly, aliphatic volatile organic compounds (VOCs) like butane, propane, and pentane can adsorb onto pristine graphene, influencing its electrical properties and making graphene suitable for chemiresistive sensors.^[^ ^33^ ^]^

**Figure 1:**
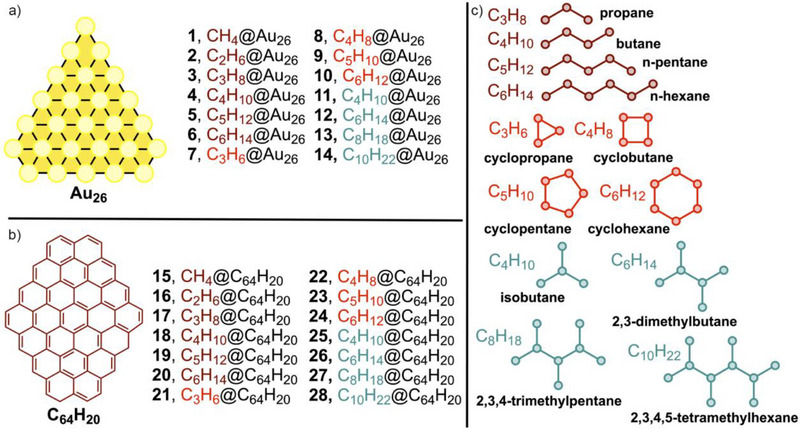
Complexes **1**–**14** a), **15**–**28** b), and schematic representation of the alkane molecules used herein c).

Although challenges still exist with regard to toxicity, scalability, and long‐term stability of these materials, there is also room for further molecular understanding of the noncovalent binding phenomena associated with the recognition and adsorption of aliphatic organic compounds onto them. Herein, we have performed a theoretical comparative analysis between noncovalent C─H···Au and C─H···π bonds using the PBE0‐D3/def2‐TZVP level of theory. Concretely, we have used a series of alkanes showing different i) linear, ii) cyclic, and iii) branched chain structures. On the other hand, Au_26_ and C_64_H_20_ layers were used, mimicking a decahedral Au nanoparticle's face and a graphene surface portion, respectively (see Figure 1 below). The supramolecular complexes obtained were compared in terms of their geometry and stability, unveiling interesting trends regarding i) the length, ii) the shape, and iii) the substitution degree of the aliphatic chain. In addition, they were characterized using the Quantum Theory of Atoms in Molecules (QTAIM) and Non‐Covalent Interaction plot (NCIplot) methodologies, which shed light on the aliphatic C─H bond distribution along the complexes as well as the extension in real space of the interactions studied herein. Lastly, the Energy Decomposition Analysis (EDA) technique was useful to unveil resemblances/differences in the energetic contributors of both supramolecular forces. We believe that this conceptual study will be of interest to both supramolecular and materials science scientists, owing to the implications of these noncovalent interactions in molecular recognition, catalysis, and solid‐state chemistry.

## 2 Computational Methods

The interaction energies of all complexes included in this study were computed at the PBE0^[^ ^34^, ^35^ ^]^‐D3^[^ ^36^ ^]^/def2‐TZVP^[^ ^37^ ^]^ level of theory. The combination of PBE0 and triple zeta basis set is sufficient to describe the present systems, as it has been used in several previous studies on the topic.^[^ ^20^, ^24^, ^26^ ^]^ In addition, a small set of complexes was computed using the TPSS^[^ ^38^ ^]^ and CAM‐B3LYP^[^ ^39^ ^]^ methods to test their performance in this type of system. The calculations have been performed using the program TURBOMOLE version 7.7.^[^ ^40^ ^]^ More in detail, the Au layer was built using the geometries retrieved from the study of Sawabe and collaborators^[^ ^41^ ^]^ by utilizing a portion of 26 atoms belonging to the 282‐atom cluster (which belongs to one of the “faces” of the metal decahedron structure). On the other hand, the C layer was built and its structure optimized to offer a similar molecular surface to the Au layer, thus making the results comparable between them. Both layers were kept frozen while the alkane molecules were left freely to explore the most favourable disposition.

The interaction energies (see Table 1) were calculated using the supermolecule approximation (Δ*E* = *E*_Alkane‐Layer complex_ − E_Alkane_–E_layer_). On the other hand, the ΔΔE values gathered in Table 1 correspond to the energetic difference between complexes **1**/**15** and complexes **2**–**6** and **16**–**20** (to energetically quantify the reinforcement of the interaction upon elongating the alkane molecule), between complexes **7**/**21** and complexes **8**–**10** and **22**–**24** (to energetically quantify the reinforcement of the interaction upon increasing the ring size) and between complexes **11**/**25** and complexes **12**–**14** and **26**–**28** (to energetically quantify the effect of increasing the number of ramifications in the main alkyl chain).

**Table 1:** Average electrostatic potential surface values over the H atoms of the alkane molecules used in this study at the PBE0‐D3/def2‐TZVP level of theory (*V*_H_, in kcal mol^−1^). The maximum (*V*_max_) and minimum (*V*_min_) electrostatic potential values are also included in kcal mol^−1^.

Compound	*V*_H_	*V*_max_	*V*_min_
**CH_4_ (methane)**	+9.1	+10	−3
**C_2_H_6_ (ethane)**	+7.5	+8	−3
**C_3_H_8_ (propane)**	+7.4	+8	−4
**C_4_H_10_ (butane)**	+7.0	+8	−4
**C_5_H_12_ (n‐pentane)**	+6.9	+8	−4
**C_6_H_14_ (n‐hexane)**	+6.8	+8	−4
**C_3_H_6_ (cyclopropane)**	+10.6	+11	−13
**C_4_H_8_ (cyclobutane)**	+7.4	+8	−4
**C_5_H_10_ (cyclopentane)**	+6.8	+8	−4
**C_6_H_12_ (cyclohexane)**	+6.3	+7	−3
**C_4_H_10_ (isobutane)**	+7.4	+8	−3
**C_6_H_14_ (2,3‐dimethylbutane)**	+7.4	+8	−3
**C_8_H_18_ (2,3,4‐trimethylpentane)**	+7.2	+8	−4
**C_10_H_22_ (2,3,4,5‐tetramethylhexane)**	+7.2	+9	−4

The optimizations were carried out without imposing symmetry on the systems. The optimized structures do not correspond to fully relaxed geometries since it was considered that the molecular systems modeled (an Au nanoparticle and graphene) preserve their structure upon interacting with the alkane moieties; therefore, frequency analysis calculations were not performed.

The Molecular Electrostatic Potential (MEP) surfaces were computed at the PBE0‐D3/def2‐TZVP level of theory by means of the Gaussian 16 software^[^ ^42^ ^]^ and analyzed using the Gaussview 5.0 program.^[^ ^43^ ^]^ The calculations for the QTAIM analyses were carried out at the PBE0‐D3/def2‐TZVP level of theory using the Multiwfn software.^[^ ^44^ ^]^ In addition, the EDA^[^ ^45^, ^46^ ^]^ scheme was used to understand the role of electrostatics, exchange‐repulsion, orbital, dispersion, and electron correlation contributions in the formation of the noncovalent complexes studied herein at the PBE0‐D3/def2‐TZVP level of theory, also using TURBOMOLE 7.7 software.

Lastly, the NCIplot^[^ ^47^ ^]^ isosurfaces correspond to both favorable and unfavorable interactions, as differentiated by the sign of the second‐density Hessian eigenvalue and defined by the isosurface color. The color scheme is a red−yellow−green−blue scale, with red for repulsive (ρ_cut_^+^) and blue for attractive (ρ_cut_^−^) NCI interaction density. Yellow and green surfaces correspond to weak repulsive and weak attractive interactions, respectively. The surfaces were visualized using the Visual Molecular Dynamics (VMD) software.^[^ ^48^ ^]^

## 3 Results and Discussion

### 3.1 Electrostatic Potential Surface Analyses

**Figure 2:**
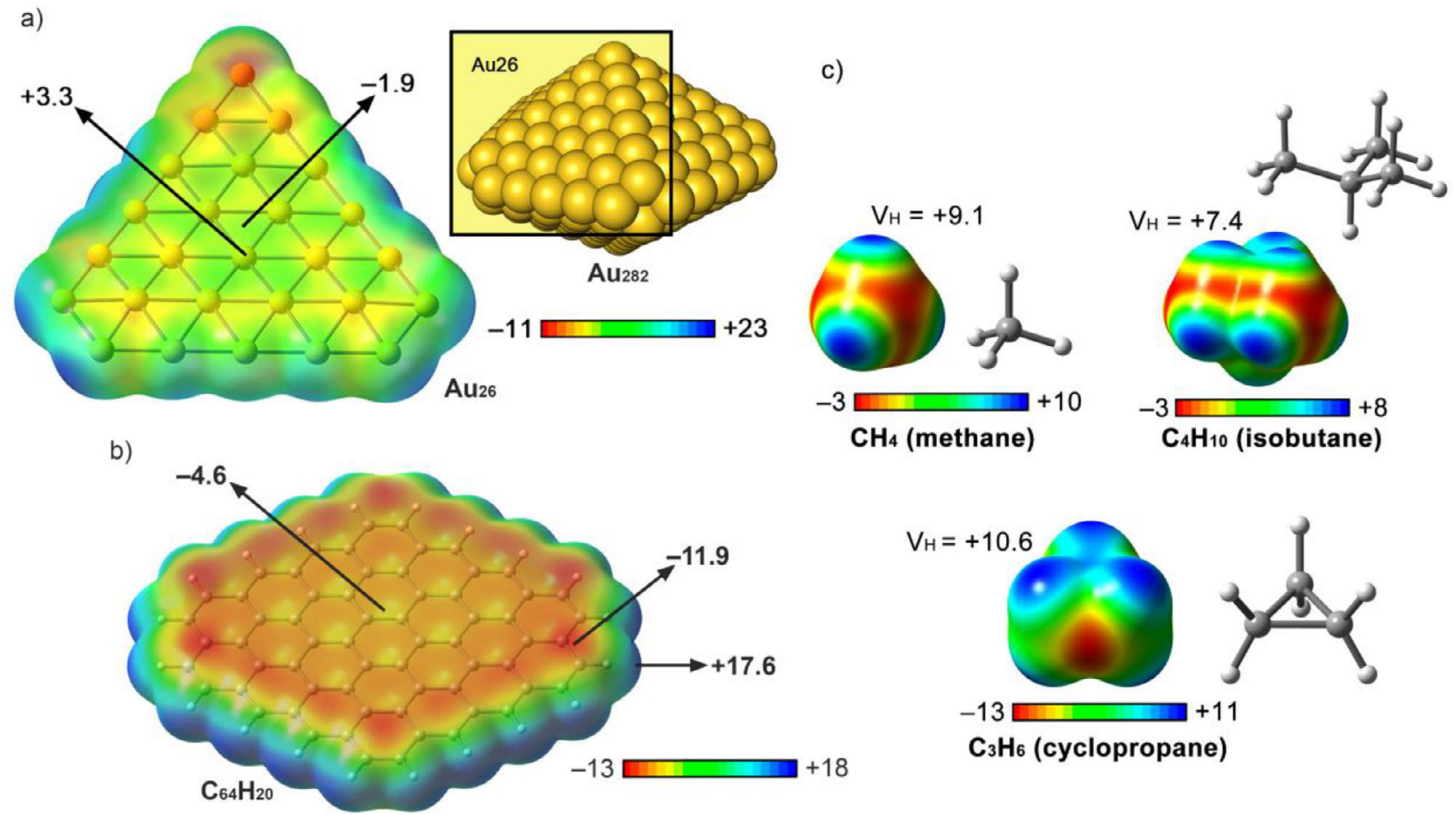
a,b) MEP surfaces of Au_26_ and C_64_H_20_. The energy values at specific points of the surface are given in kcal·mol^−1^ (0.001 a.u.). c) MEP surfaces of methane, isobutane, and cyclopropane. The average electrostatic potential value over the H atoms (VH) is also given in kcal mol^−1^ (0.001 a.u.).

We started by computing the MEP surfaces of the Au_26_ and C_64_H_20_ models (see Figure 2), and the results showed the presence of both electron‐rich and electron‐poor regions in both layered structures. More in detail, in the case of Au_26_ (Figure 2a) we found positive electrostatic potential regions located over the Au atoms, which accounted for the presence of holes (exhibiting a MEP value of + 3.3 kcal mol^−1^), while also negative potential regions located in between the metal atoms confirming the presence of lumps (showing a MEP value of −1.9 kcal mol^−1^). These positive and negative potential regions confer a dual behavior to the Au_26_ layer, being capable of favorably interacting with both electron‐rich (C) and electron‐poor sides (H) of the alkane molecules used herein. On the other hand, in the case of the C_64_H_20_ moiety (Figure 2b), a π‐basic surface was found along the graphene layer, exhibiting negative potential values of −4.6 and −11.9 kcal mol^−1^ at the center and peripheral sites, respectively. In this case, the electron‐poor regions were located at the peripheral H atoms, with a MEP value of +17.6 kcal mol^−1^.

Lastly, in Figure 2c, three MEP surfaces corresponding to methane, cyclopropane, and isobutane are shown as representative examples, exhibiting electropositive sites at the H atoms, as expected (see Table 1 for the rest of the MEP values). As the increase in the number of H atoms yields different electrostatic potentials values depending on their position in the aliphatic chain as well as the molecular symmetry, we measured the average potential value of all H atoms present in the molecule (V_H_) since it provides a more representative picture of the electrostatic properties of the H atoms in the alkane molecules used herein. In general, these average MEP values decrease (become less positive) while increasing the chain length, the size of the ring, or the number of branched groups. For instance, between methane and n‐hexane, we observed an average MEP decrease of around 2 kcal mol^−1^, while between cyclopropane and cyclohexane, this difference was increased to 4.3 kcal mol^−1^. Lastly, in the case of the branched alkanes, this value became negligible (0.2 kcal mol^−1^).

In general, the slight differences observed in the negative MEP values measured at the center of both the Au_26_ and C_64_H_20_ layers point out to a similar contribution of electrostatics. However, the electron‐poor character of the H atoms from the alkyl chains became less positive while increasing the size of the main chain or the number of branched groups due to electron‐donor inductive effects. Therefore, to clarify this point and discuss the role of electrostatics in the supramolecular complexes studied herein, an energy partition analysis was carried out (see EDA study below).

### 3.2 Energetic and Geometrical Study

In Table 2, the results regarding the C─H···Au and C─H···π bonds strength are shown. As noticed, in all the cases, the interaction energy values are negative and of moderately strong to weak nature, encompassed between −21.6 and −3.2 kcal mol^−1^. More in detail, a similar behavior was observed among the two families of complexes, that is, complexes **13** and **27** involving 2,3,4‐trimethylpentane achieved the most favorable interaction energy values of each series (−21.6 and −12.6 kcal mol^−1^, respectively). On the other hand, complexes **1** and **15** involving methane obtained the lowest strength of each series (−5.4 and −3.2 kcal mol^−1^, respectively). In general, the interaction energies obtained from the C–H···Au complexes (**1**–**14**) were more favorable than their C–H··· π analogs (**15**–**28**), thus remarking the importance of this unconventional bond in the formation of stable noncovalent adducts.

**Table 2:** Interaction energies and interaction energy differences (ΔE and ΔΔE, in kcal·mol^−1^), intermolecular C/Au···H distances (d, in Å), distance/sum of the van der Waals radii ratio (d/∑_vdW_) and values of the C/Au···H–C angle (∠, in degrees) for complexes **1**–**28** at the PBE0‐D3/def2‐TZVP level of theory. Values inside parentheses were obtained using the TPSS functional, while values inside brackets were obtained using CAM‐B3LYP for complexes **3**, **8**, **12**, **17**, **22**, and **26**.

Complex	ΔE	ΔΔE	d^a^ ^)^	d/∑_vdW_ ^a^ ^)^	∠^a^ ^)^
**1**	−5.4	‐	3.139	0.86	105.2
**2**	−8.2	−2.8	2.982	0.82	178.0
**3**	−11.4 (−12.8) [−11.2]	−3.2	2.921 (2.872) [2.924]	0.80 (0.79) [0.80]	131.5 (131.2) [131.6]
**4**	−14.2	−2.8	2.905	0.80	128.6
**5**	−17.6	−3.4	2.749	0.75	154.8
**6**	−20.3	−2.7	2.759	0.76	153.9
**7**	−9.7	‐	2.940	0.81	122.1
**8**	−13.1 (−14.9) [−13.0]	−3.4	2.847 (2.769) [2.851]	0.78 (0.76) [0.78]	155.6 (156.2) [160.0]
**9**	−15.1	−2.0	2.747	0.75	160.8
**10**	−15.6	−0.5	2.740	0.75	148.7
**11**	−12.3	‐	2.846	0.78	149.3
**12**	−18.2 (−21.2) [−17.4]	−5.9	2.592 (2.520) [2.610]	0.71 (0.69) [0.72]	147.1 (146.4) [147.9]
**13**	−21.6	−3.4	2.571	0.70	140.2
**14**	−20.7	+0.9	2.586	0.71	134.9
**15**	−3.2	‐	3.026	1.02	103.6
**16**	−4.9	−1.7	2.899	0.98	175.2
**17**	−6.4 (−6.5) [−6.1]	−1.5	2.722 (2.717) [2.723]	0.92 (0.91) [0.92]	155.9 (156.0) [156.0]
**18**	−8.9	−2.5	2.941	0.99	124.9
**19**	−9.9	−1.0	2.755	0.93	138.1
**20**	−11.8	−1.9	2.791	0.94	149.2
**21**	−5.5	‐	2.794	0.94	153.5
**22**	−7.4 (−7.5) [−7.2]	−1.9	2.816 (2.815) [2.776]	0.95 (0.95) [0.93]	137.1 (136.9) [139.7]
**23**	−8.0	−0.6	2.659	0.89	170.2
**24**	−8.6	−0.6	2.697	0.91	166.7
**25**	−6.8	‐	2.701	0.91	177.5
**26**	−10.7 (−10.9) [−10.2]	−3.9	2.701 (2.699) [2.701]	0.91 (0.91) [0.91]	163.0 (162.8) [163.0]
**27**	−12.6	−1.9	2.652	0.89	166.4
**28**	−9.9	+2.7	2.590	0.87	158.3

^a)^ Values given as the shortest C/Au···H distance.

^b)^ The d/∑_vdW_ values were calculated using Álvarez vdW radii values.^[^ ^50^ ^]^

**Figure 3:**
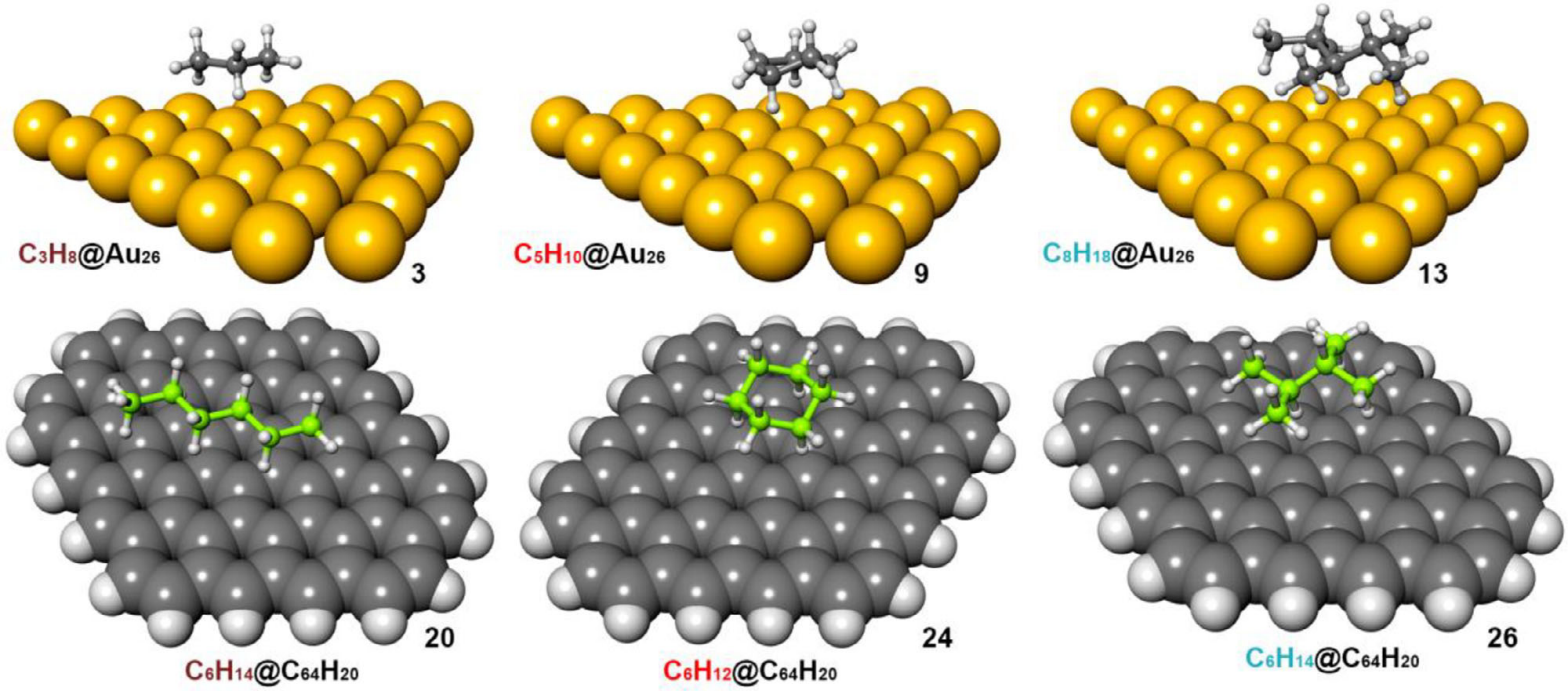
Optimized geometries at the PBE0‐D3/def2‐TZVP level of theory of complexes **3**, **9**, **13**, **20**, **24**, and **26**. The Au vdW radius was escalated at the 75% of its value for clarity.

First, among complexes **1**–**6** and **15**–**20** involving linear alkane chains, a progressive reinforcement of either C─H···Au or C─H···π bonds was observed, as they are known to be additive forces.^[^ ^29^, ^49^ ^]^ This reinforcement is more pronounced in the former, with ΔΔE differences comprised between −3.4 and −2.7 kcal mol^25001^, while in the latter, these energetic differences ranged between −2.5 and −1.0 kcal mol^−1^. Aside from these energetic variations, the geometry of both series of complexes is similar, showing the alkane chain in a parallel arrangement to the Au/C layer (see Figure 3 for complexes **3** and **20** and the Supporting Information for the cartesian coordinates regarding the rest of the supramolecular assemblies).

Second, among complexes **7**–**10** and **21**—**24**, involving cyclic alkane molecules, the interaction energy values ranged between −15.6 and −9.7 kcal·mol^−1^ in the case of Au involving complexes and between −8.6 and −5.5 kcal mol^−1^ in the case of the graphene set. Similar to the tendency observed with the open chain alkane moieties, a reinforcement in the strength of both interactions was observed upon increasing the size of the cyclic molecule, owing to an increase in the number of C─H···Au and C─H···π bonds established. Again, this reinforcement was more pronounced in the case of complexes **7** to **10**, with ΔΔE values comprised between ─3.4 and ─0.5 kcal mol^−1^, while in the case of complexes **21**–**24**, the ΔΔE values ranged between −1.9 and −0.6 kcal mol^−1^. In terms of geometry, the cyclic alkane molecules are disposed pointing their maximum amount of C─H bonds to either the Au or C layer (see Figure 3 for complexes **9** and **24**).

Lastly, for complexes **11**–**14** and **25**–**28** involving branched aliphatic molecules, we also observed for both the Au and graphene layers an increase in the interaction energy strength from complex **11** (−12.3 kcal mol^−1^) to **13** (−21.6 kcal·mol^−1^) and from complex **25** (−6.8 kcal mol^−1^) to **27** (−12.6 kcal mol^−1^). However, we found a decrease in the strength of both supramolecular bonds from complexes **13** and **27** involving 2,3,4‐trimethylpentane to complexes **14** (−20.7 kcal mol^−1^) and **28** (−9.9 kcal mol^−1^) involving 2,3,4,5‐tetramethylhexane. This decrease in the interaction energy values is likely due to the level of steric hindrance exhibited by this alkane, which prevented some methyl groups from properly orienting their C─H bonds to the Au/C layer (see Figure 3 for the geometries regarding complexes **13** and **26** as two representative examples).

In Table 2, the data regarding distance and angle parameters of complexes **1**–**28** are also included. Concretely, we found that the H···Au/C distances lay within values slightly below the sum of the H + Au and H + C van der Waals radii, as denoted by the d/∑vdW ratios obtained. Only in the case of complex **15**, this coefficient was slightly above 1. Additionally, we measured the C/Au···H–C angle for the entire set of complexes, resulting in values comprised between 103.6 and 178.0 degrees, thus indicating a non‐preferred directional behavior of both supramolecular bonds. Lastly, we have optimized several selected complexes (**3**, **8**, **12**, **17**, **2** **2,** and **26**) using the TPSS and CAM‐B3LYP functionals (see values in parentheses and in brackets in Table 2). As observed, both the energetics and intermolecular distances lie within the same range compared to the hybrid PBE0 functional, thus demonstrating their reliability when treating this type of supramolecular bonds.

### 3.3 QTAIM and NCIplot Analyses

**Figure 4:**
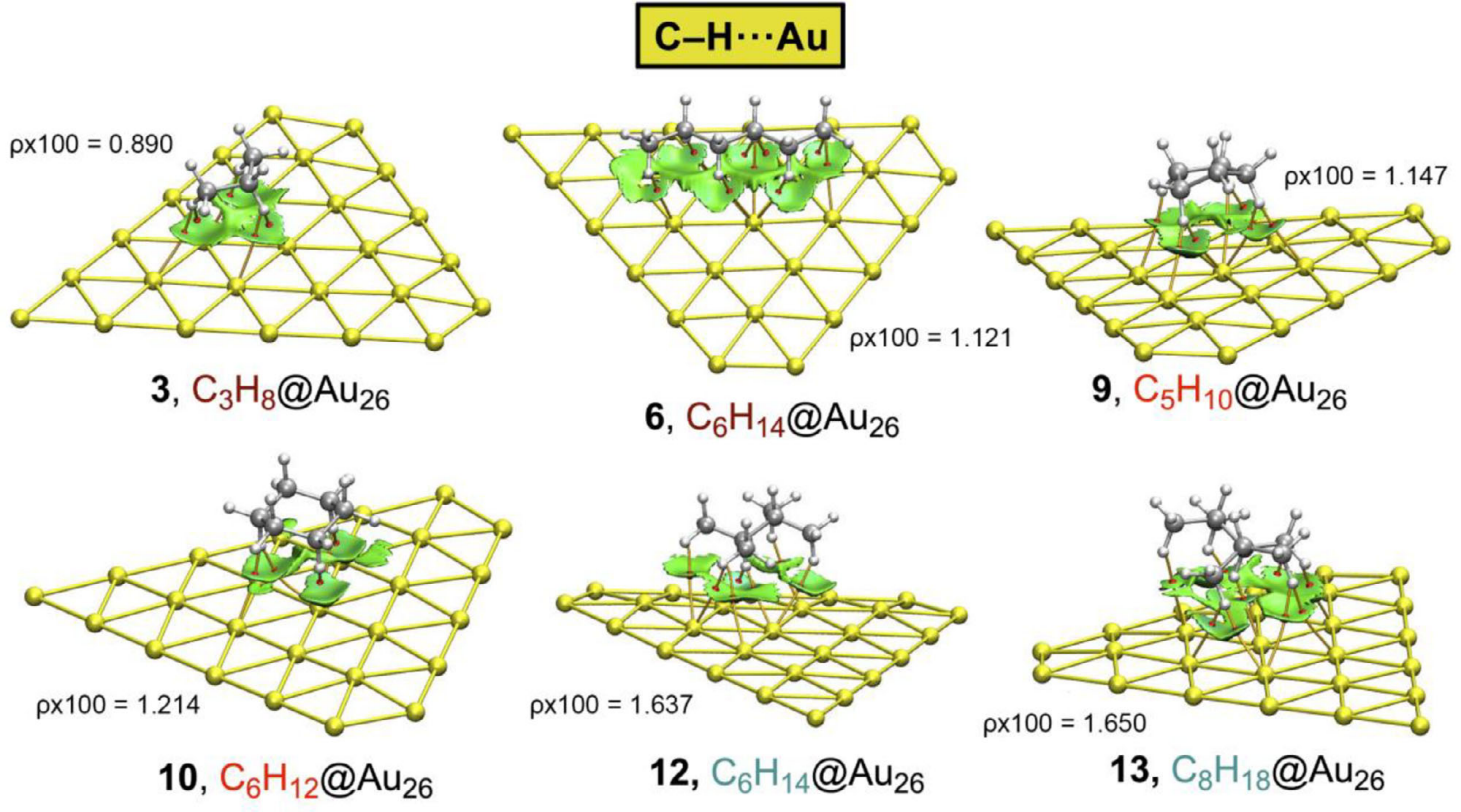
NCIplot analysis and QTAIM distribution of intermolecular bond critical points (bcps in red spheres) and bond paths in complexes **3**, **6**, **9**, **10**, **12**, and **13** involving Au_26_. The values of the density at the bcp involving the shortest intermolecular distance in each complex are also shown in a.u. NCIplot surfaces only include intermolecular contacts between the alkane molecule and the layer. NCIplot colour range−0.04 a.u. ≤ (signλ_2_)ρ ≤ +0.04 a.u. Isosurface value RDG = 0.5 and ρ cutoff 0.05 a.u.

**Figure 5:**
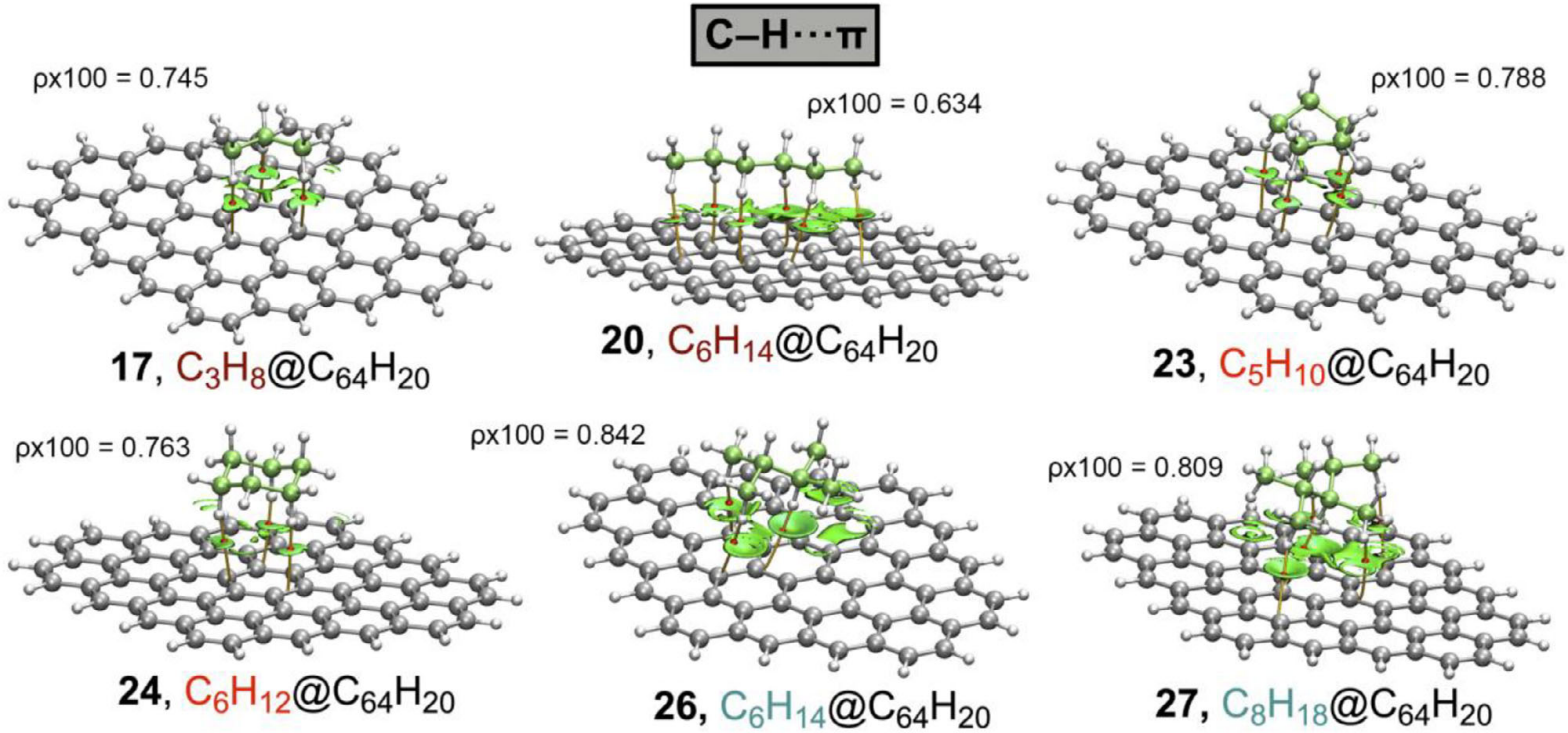
NCIplot analysis and QTAIM distribution of intermolecular bond critical points (bcps in red spheres) and bond paths in complexes **17**, **20**, **23**, **24**, **26**, and **27** involving C_64_H_20_. The values of the density at the bcp involving the shortest intermolecular distance in each complex are also shown in a.u. NCIplot surfaces only include intermolecular contacts between the alkane molecule and the layer. NCIplot colour range − 0.04 au ≤ (signλ_2_)ρ ≤ +0.04 au. Isosurface value RDG = 0.5 and ρ cutoff 0.05 a.u.

**Figure 6:**
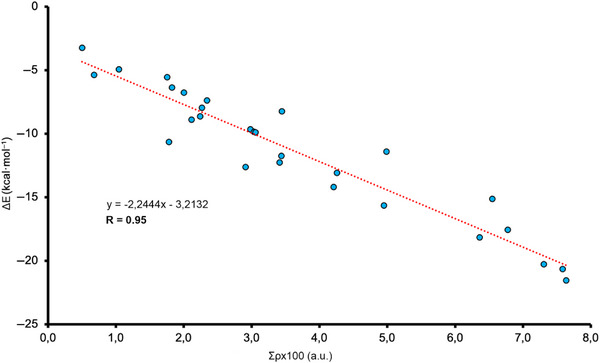
Graphical representation of the interaction energy values (ΔE, in kcal·mol^−1^) versus the sum of the density values at the intermolecular bcps (Σρx100, in a.u.) for complexes **1**–**28**.

In Figures 4, 5, 6 and Table 3, the results regarding the QTAIM and NCIplot analyses are shown for a set of representative complexes (**3**, **6**, **9**, **10**, **12**, and **13** involving Au_26_ and **17**, **20**, **23**, **24**, **26**, and **27** involving C_64_H_20_), see also Figures S1 and S2 in SI for the rest of the complexes. As noted, for the first set of complexes involving Au_26_, several bond critical points (bcps) and bond paths characterize the C─H···Au bonds, since they connect the H atoms from the alkane moiety to the Au atoms from the metal layer. The same picture is observed in the case of those complexes involving C_64_H_20_, which exhibited bcps and bond paths connecting the alkane C–H bonds to the C atoms of the graphene model.

**Table 3:** Values of the density at the bond critical points (ρ×100, in a.u.) that characterize C─H···Au and C─H···π bonds present in complexes **3**, **6**, **9**, **10**, **12,** and **13** involving Au_26_ and **17**, **20**, **23**, **24**, **26,** and **27**, involving C_64_H_20_. In addition, the values of the Laplacian of ρ (∇^2^ρ×100), the potential (*V* × 100), kinetic (*G*×100) energy densities, as well as the −*G/V* ratios are also indicated in a.u.

Complex	ρ × 100^a)^	∇^2^ρ × 100	*V* × 100	*G *× 100	−*G/V*
**3**	0.890	2.403	−0.474	0.537	1.13
**6**	1.121	2.927	−0.617	0.675	1.09
**9**	1.147	2.949	−0.628	0.682	1.09
**10**	1.214	3.127	−0.677	0.729	1.08
**12**	1.637	4.185	−0.975	1.011	1.04
**13**	1.650	4.474	−1.023	1.071	1.05
**17**	0.745	2.170	−0.372	0.457	1.23
**20**	0.634	1.865	−0.309	0.388	1.25
**23**	0.788	2.221	−0.390	0.473	1.21
**24**	0.763	2.151	−0.375	0.457	1.22
**26**	0.842	2.693	−0.469	0.571	1.22
**27**	0.809	2.364	−0.406	0.498	1.23

^a)^ Only the bcps involving the shortest H···Au/C distance were considered.

If the layer is kept the same when comparing different complexes, it can be observed that the distribution of bcps and bond paths varies depending on the size and shape of the alkane moiety, as expected. However, the number of bcps and bond paths (as well as the size of the NCIplot isosurface) also varies when keeping the same alkane moiety, being those complexes involving Au being more bcp populated and with larger NCI surfaces. This is likely due to the larger size of Au compared to C, which allowed the establishment of a larger number of interactions at a given intermolecular distance. This result agrees with the larger interaction energy values obtained for complexes **1**–**14** involving Au_26_ (see above), as the Au layer provides a larger number of interaction sites for the alkane molecule to interact.

Regarding the NCIplot analysis, in all the cases (see also Figures S1 and S2 in SI), we found a greenish isosurface located between the alkane moiety and the Au/C layer, indicating the presence of a weak but favorable interaction between both partners. All the C─H interactions contributed nearly equally to the stabilization of the supramolecular assemblies, as the greenish color remains the same within all the isosurfaces. Lastly, in Figure 6, we have represented the interaction energy values obtained (ΔE, in kcal·mol^−1^) versus the total values of the density at the intermolecular bcps for each complex (Σρx100, in a.u.), obtaining a very nice Pearson's R correlation coefficient (R = 0.95).

This result indicates that the strength of both supramolecular bonds can be predicted using their respective topological parameters. This is of remarkable importance, since in all these complexes studied, only H···Au/C bcps were found with no ancillary interactions present in the system, indicating that this dataset of molecules can be used to train accurate structural‐energetic predictive models. As a final remark, in Table 3 we have also included the value of the Laplacian at the intermolecular bcps for each complex, exhibiting positive values in all the cases, as is common in closed‐shell calculations, as well as the potential (V) and kinetic (G) densities. Lastly, the −G/V ratio remained close to 1 in all the cases (ranging between 1 and 1.25), further confirming the noncovalent nature of the interactions studied herein. The rest of the complexes are presented in Table S1.

### 3.4 EDA Study

**Figure 7:**
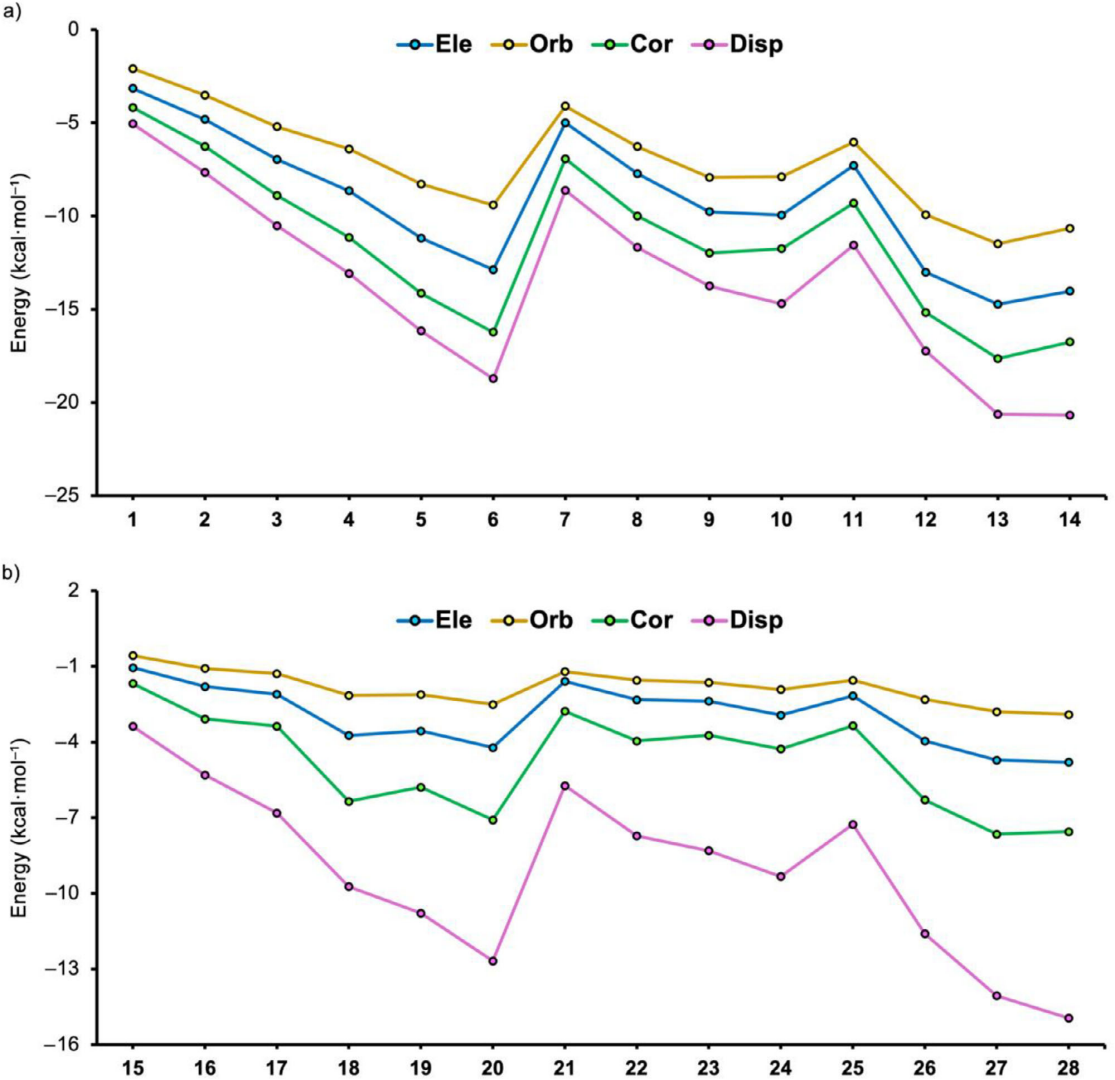
Graphical representation of the Ele, Orb, Cor, and Disp terms for complexes **1**–**14** involving Au_26_ and **15**–**28** involving C_64_H_20_.

As the final stage of our work, we conducted an EDA study on the supramolecular complexes studied herein (see Table 4 and Figure 7). The energy partition scheme used unveiled the contribution of electrostatics (Ele), exchange‐repulsion (Ex‐rep), orbital (Orb), dispersion (Disp), and electron correlation (Cor) terms (only the attractive terms were plotted in Figure 7). As noted in Figure 7a, for complexes **1**–**14**, we obtained a similar energy descriptor profile, where the dispersion and electron correlation terms are the most prominent contributors, followed by electrostatics and orbital terms. This behavior can be clearly observed from complexes **1**–**6** involving open alkyl chains, where an almost linear increase (the values became more negative) among all four favorable energy descriptors was observed, in agreement with the reinforcement of the interaction energy values discussed above (see Table 2).

**Table 4:** Values of the exchange‐respulsion (*E*_ex‐rep_), electrostatics (*E*_ele_), orbital (*E*_orb_), electron correlation (*E*_cor_), and dispersion (E_disp_) energy terms in kcal mol^−1^ complexes **1**–**14** involving Au_26_ and **15**–**28** involving C_64_H_20_.

Complex	*E*_ex‐rep_	*E*_ele_	*E*_orb_	*E*_cor_	*E*_Disp_
**1 (15)**	9.1 (3.5)	−3.2 (−1.0)	−2.1 (−0.6)	−4.2 (−1.7)	−5.1 (−3.4)
**2 (16)**	14.1 (6.4)	−4.8 (−1.8)	−3.5 (−1.1)	−6.3 (−3.1)	−7.7 (−5.3)
**3 (17)**	20.2 (7.3)	−7.0 (−2.1)	−5.2 (−1.3)	−8.9 (−3.4)	−10.5 (−6.8)
**4 (18)**	25.1 (13.2)	−8.7 (−3.7)	−6.4 (−2.1)	−11.2 (−6.4)	−13.1 (−9.7)
**5 (19)**	32.2 (12.5)	−11.2 (−3.6)	−8.3 (−2.1)	−14.4 (−5.8)	−16.2 (−10.8)
**6 (20)**	36.9 (14.8)	−12.9 (−4.2)	−9.4 (−2.5)	−16.2 (−7.1)	−18.7 (−12.7)
**7 (21)**	15.1 (5.8)	−5.0 (−1.6)	−4.1 (−1.2)	−6.9 (−2.8)	−8.6 (−5.7)
**8 (22)**	22.7 (8.3)	−7.7 (−2.3)	−6.3 (−1.5)	−10.0 (−4.0)	−11.7 (−7.7)
**9 (23)**	28.4 (8.2)	−9.8 (−2.4)	−7.9 (−1.6)	−12.0 (−3.7)	−13.8 (−8.3)
**10 (24)**	28.7 (9.9)	−9.9 (−2.9)	−7.9 (−1.9)	−11.8 (−4.3)	−14.7 (−9.3)
**11 (25)**	22.0 (7.7)	−7.3 (−2.2)	−6.0 (−1.5)	−9.3 (−3.4)	−11.6 (−7.3)
**12 (26)**	37.2 (13.6)	−13.0 (−4.0)	−9.9 (−2.3)	−15.2 (−6.3)	−17.2 (−11.6)
**13 (27)**	42.7 (16.6)	−14.7 (−4.7)	−11.5 (−2.8)	−17.7 (−7.6)	−20.6 (−14.1)
**14 (28)**	40.6 (16.7)	−14.0 (−4.8)	−10.7 (−2.9)	−16.8 (−7.6)	−20.7 (−14.9)

For complexes **7** to **10** involving cyclic alkanes, the picture is quite similar, being the dispersion and correlation terms, the most predominant contributors, although they ranged between lower magnitude values compared to complexes **1** to **6**, in agreement with the interaction energies obtained. Due to this, a less steep profile was obtained compared to the previous set. In fact, the energy descriptor values of complexes **9** and **10** are quite similar. Furthermore, among complexes **11** to **14** involving branched cyclic alkanes, we observed the same tendency as for linear and cyclic alkanes in terms of energy descriptor hierarchy, with a pronounced increase in all four energy component values from complex **11** to **13**. On the other hand, complex **14** showed a very similar dispersion term contribution compared to complex **13** and a less negative electron correlation, electrostatics and orbital terms values, leading to a less favorable interaction energy value, as shown in Table 2.

In the case of complexes **15**–**28** involving C_64_H_20_ (see Figure 7b), the same trend was observed compared to complexes **1** to **14** in terms of the energy descriptor predominance. However, these complexes exhibited a clear dispersion‐based nature, in agreement with previous computational studies,^47^ while correlation, electrostatics, and orbital terms exhibited values of similar magnitude. In addition, the variation of the energy descriptor values from one complex to another is much more abrupt in this set of complexes compared to the previous one, where a smoother energy transition was observed among the complexes that shared the same type of alkyl chain.

## 4 Conclusion

Herein, we have theoretically compared the structural and energetic features of C─H···Au and C─H···π bonds involving alkanes of different length, shape, and steric hindrance, and i) an Au_26_ surface and ii) a graphene layer at the PBE0‐D3/def2‐TZVP level of theory. Results show similarities in the physical nature and geometrical features of both supramolecular bonds, such as their dispersion‐based nature, their additive character, as well as their poor directionality. Additionally, the hierarchical order of the rest of the energy components (electrostatics, orbital, and electron correlation) is similar in both interactions, although they differ in magnitude, playing a much more noticeable role in the case of Au_26_ complexes, which ultimately resulted in the energetic differences observed in the interaction energy values. Another interesting finding was derived from the QTAIM and NCIplot analyses related to the very good correlation coefficient obtained when representing in the same graphic the values of the interaction energy versus the total values of the density at the intermolecular bcps for each complex, indicating common traits between both interactions with respect to their wavefunction topological parameters, which can be used as a predictive tool of their strength. We believe that this conceptual study will be of interest to both supramolecular and materials science scientists, owing to the implications of these noncovalent interactions in molecular recognition, catalysis, and solid‐state chemistry.

## Supporting Information

The Supporting Information contains Figures S1 and S2, Table S1, and the cartesian coordinates of complexes **1**–**28**.

## Conflict of Interests

The authors declare no conflict of interest.

## Supporting information



Supporting Information

## Data Availability

The data that support the findings of this study are available in the supplementary material of this article.
