# Supplementary material for: Are Noncovalent C─H⋯ Au Bonds Comparable to C─H⋯ π Bonds? A Theoretical Perspective
Source: Chem Asian J. 2025 Jul 24;20(19):e00736. doi: 10.1002/asia.202500736 (PMC12498187; doi:10.1002/asia.202500736)
Supplement: Supplementary file 1 — Supporting Information [file ASIA-20-e00736-s001.docx]

Supporting Information

**Are Noncovalent C–H···Au Bonds Comparable to C–H···π Bonds? A Theoretical Perspective**

Sergi Burguera,^[a]^ and Antonio Bauzá*^[a]^

^[a]^Departament de Química, Universitat de les Illes Balears, Ctra. de Valldemossa km 7.5, 07122 Palma de Mallorca (Baleares), Spain

E-mail: antonio.bauza@uib.es

**Figure S1 page 2**

**Figure S2 page 3**

**Table S1 page 4**

**Cartesian coordinates of complexes 1 to 28 page 5**


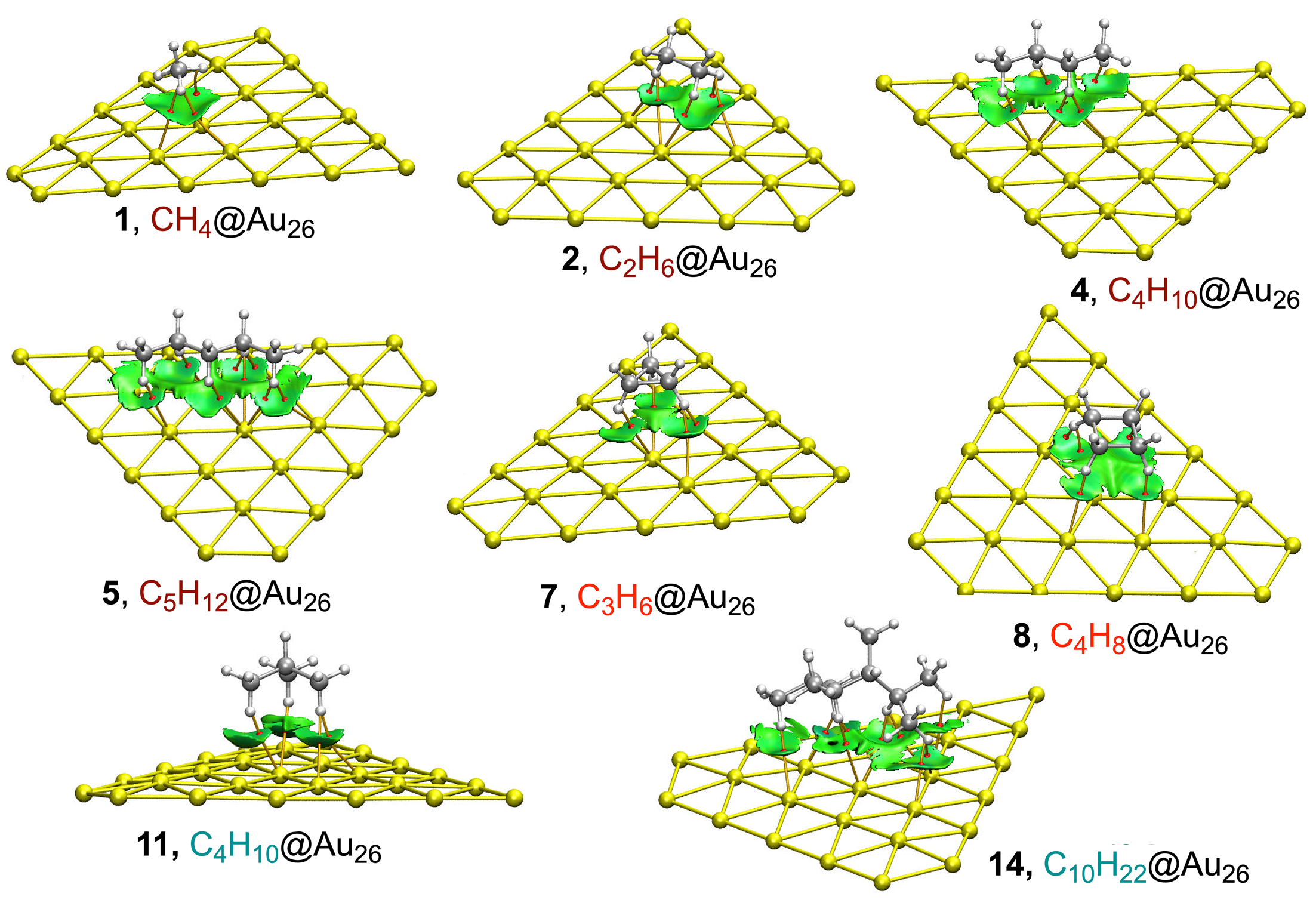


**Figure S1.** NCIplot analysis and QTAIM distribution of intermolecular bond critical points (bcps in red spheres) and bond paths in complexes **1**, **2**, **4**, **5**, **7**, **8**, **11**, and **14** involving Au_26_. NCIplot surfaces only include intermolecular contacts between the alkane molecule and the layer. NCIplot colour range −0.04 au ≤ (signλ_2_)ρ ≤ +0.04 au. Isosurface value RDG = 0.5 and ρ cutoff 0.05 au.


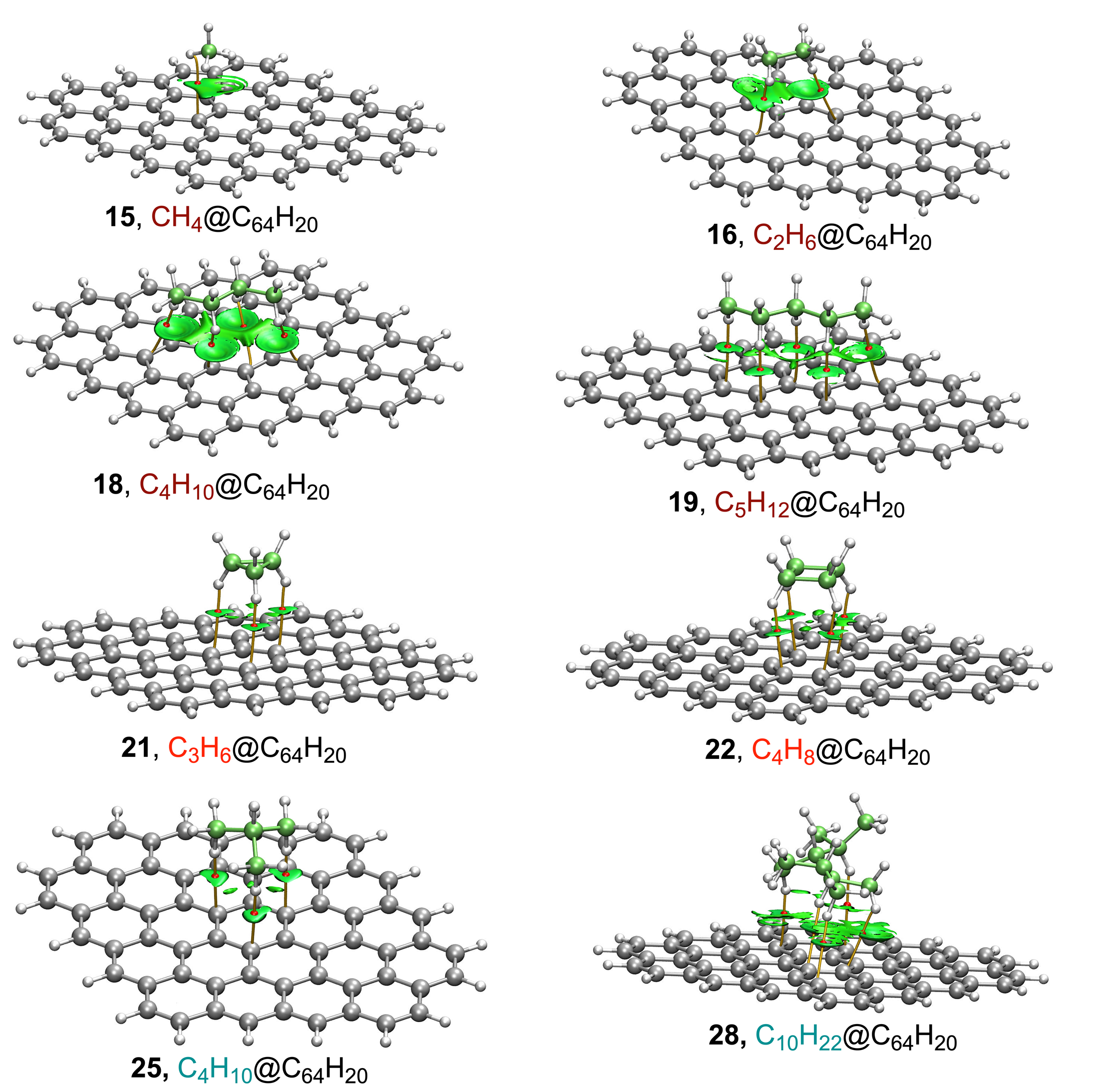


**Figure S2.** NCIplot analysis and QTAIM distribution of intermolecular bond critical points (bcps in red spheres) and bond paths in complexes **15**, **16**, **18**, **19**, **21**, **22**, **25**, and **28** involving C_64_H_20_. NCIplot surfaces only include intermolecular contacts between the alkane molecule and the layer. NCIplot colour range −0.04 au ≤ (signλ_2_)ρ ≤ +0.04 au. Isosurface value RDG = 0.5 and ρ cutoff 0.05 au.

**Table S1.** Values of the density at the bond critical points (ρx100, in a.u.) that characterize C–H···Au and C–H···π bonds present in complexes **3**, **6**, **9**, **10**, **12** and **13** involving Au_26_ and **17**, **20**, **23**, **24**, **26** and **27** involving C_64_H_20_. In addition, the values of the laplacian of ρ (∇^2^ρx100), the potential (Vx100), kinetic (Gx100) energy densities as well as the –G/V ratio are also indicated in a.u.

| **Complex** | **ρx100^[a]^** | **∇^2^ρx100** | **Vx100** | **Gx100** | **−G/V** |
| --- | --- | --- | --- | --- | --- |
| **1** | 0.675 | 2.284 | –0.389 | 0.480 | 1.23 |
| **2** | 0.747 | 1.870 | –0.354 | 0.411 | 1.16 |
| **4** | 0.846 | 2.285 | –0.445 | 0.508 | 1.14 |
| **5** | 1.140 | 2.976 | –0.630 | 0.687 | 1.09 |
| **7** | 0.835 | 2.489 | –0.475 | 0.549 | 1.16 |
| **8** | 0.939 | 2.436 | –0.498 | 0.553 | 1.11 |
| **11** | 0.976 | 2.556 | –0.518 | 0.579 | 1.12 |
| **14** | 1.593 | 4.469 | –1.008 | 1.062 | 1.05 |
| **15** | 0.500 | 1.767 | –0.249 | 0.345 | 1.39 |
| **16** | 0.543 | 1.807 | –0.281 | 0.366 | 1.31 |
| **18** | 0.512 | 1.748 | –0.264 | 0.351 | 1.33 |
| **19** | 0.684 | 2.048 | –0.342 | 0.427 | 1.25 |
| **21** | 0.591 | 1.792 | –0.290 | 0.369 | 1.27 |
| **22** | 0.601 | 1.836 | –0.296 | 0.377 | 1.28 |
| **25** | 0.709 | 2.013 | –0.342 | 0.423 | 1.24 |
| **28** | 0.936 | 2.738 | –0.481 | 0.583 | 1.21 |

[a] Only the bcps exhibiting the largest density values were considered.

**Cartesian coordinates of complexes 1 to 28**

**1**

Au 0.1870624 9.5921279 0.0000000

Au 0.1387433 7.0670366 1.4910781

Au 0.1387433 7.0670366 -1.4910781

Au 0.1153996 4.5881908 2.9443772

Au 0.1153996 4.5881908 -2.9443772

Au 0.0022797 4.5997189 0.0000000

Au 0.0655694 2.0852000 4.4232456

Au 0.0655694 2.0852000 -4.4232456

Au 0.0027682 2.0955269 -1.4724210

Au 0.0027682 2.0955269 1.4724210

Au -0.0482816 -0.4001087 5.9196464

Au -0.0482816 -0.4001087 -5.9196464

Au -0.0718635 -0.4092256 -2.9689204

Au -0.0469406 -0.4153710 0.0000000

Au -0.0718635 -0.4092256 2.9689204

Au -0.1110387 -2.8998671 7.4022516

Au -0.1110387 -2.8998671 -7.4022516

Au -0.2394359 -2.8589084 -4.4410673

Au -0.1896858 -2.8672319 -1.4771707

Au -0.1896858 -2.8672319 1.4771707

Au -0.2394359 -2.8589084 4.4410673

Au 0.1153996 -5.3038203 -5.8473867

Au 0.1050302 -5.3177094 -2.9377369

Au 0.0923888 -5.3346417 0.0000000

Au 0.1050302 -5.3177094 2.9377369

Au 0.1153996 -5.3038203 5.8473867

C -3.3240390 0.2654643 1.6252237

H -2.9631545 -0.7602102 1.7071611

H -2.8871966 0.8674179 2.4232776

H -4.4105084 0.2787586 1.7121567

H -3.0254981 0.6764276 0.6609276

**2**

Au 0.1870624 9.5921279 0.0000000

Au 0.1387433 7.0670366 1.4910781

Au 0.1387433 7.0670366 -1.4910781

Au 0.1153996 4.5881908 2.9443772

Au 0.1153996 4.5881908 -2.9443772

Au 0.0022797 4.5997189 0.0000000

Au 0.0655694 2.0852000 4.4232456

Au 0.0655694 2.0852000 -4.4232456

Au 0.0027682 2.0955269 -1.4724210

Au 0.0027682 2.0955269 1.4724210

Au -0.0482816 -0.4001087 5.9196464

Au -0.0482816 -0.4001087 -5.9196464

Au -0.0718635 -0.4092256 -2.9689204

Au -0.0469406 -0.4153710 0.0000000

Au -0.0718635 -0.4092256 2.9689204

Au -0.1110387 -2.8998671 7.4022516

Au -0.1110387 -2.8998671 -7.4022516

Au -0.2394359 -2.8589084 -4.4410673

Au -0.1896858 -2.8672319 -1.4771707

Au -0.1896858 -2.8672319 1.4771707

Au -0.2394359 -2.8589084 4.4410673

Au 0.1153996 -5.3038203 -5.8473867

Au 0.1050302 -5.3177094 -2.9377369

Au 0.0923888 -5.3346417 0.0000000

Au 0.1050302 -5.3177094 2.9377369

Au 0.1153996 -5.3038203 5.8473867

C -3.3567255 0.2831043 1.4740577

H -2.7876212 -0.6409526 1.3333776

H -2.8019552 0.9020149 2.1855640

H -4.3050465 0.0119346 1.9446283

C -3.5707609 1.0092586 0.1588636

H -4.1097326 0.3866667 -0.5590711

H -4.1418959 1.9301961 0.2979657

H -2.6169482 1.2846420 -0.3007860

**3**

Au 0.1870624 9.5921279 0.0000000

Au 0.1387433 7.0670366 1.4910781

Au 0.1387433 7.0670366 -1.4910781

Au 0.1153996 4.5881908 2.9443772

Au 0.1153996 4.5881908 -2.9443772

Au 0.0022797 4.5997189 0.0000000

Au 0.0655694 2.0852000 4.4232456

Au 0.0655694 2.0852000 -4.4232456

Au 0.0027682 2.0955269 -1.4724210

Au 0.0027682 2.0955269 1.4724210

Au -0.0482816 -0.4001087 5.9196464

Au -0.0482816 -0.4001087 -5.9196464

Au -0.0718635 -0.4092256 -2.9689204

Au -0.0469406 -0.4153710 0.0000000

Au -0.0718635 -0.4092256 2.9689204

Au -0.1110387 -2.8998671 7.4022516

Au -0.1110387 -2.8998671 -7.4022516

Au -0.2394359 -2.8589084 -4.4410673

Au -0.1896858 -2.8672319 -1.4771707

Au -0.1896858 -2.8672319 1.4771707

Au -0.2394359 -2.8589084 4.4410673

Au 0.1153996 -5.3038203 -5.8473867

Au 0.1050302 -5.3177094 -2.9377369

Au 0.0923888 -5.3346417 0.0000000

Au 0.1050302 -5.3177094 2.9377369

Au 0.1153996 -5.3038203 5.8473867

C -3.4689745 0.4314418 0.9581852

H -3.4441920 -0.6551141 0.8474580

H -2.6027337 0.7190332 1.5656874

H -4.3640858 0.6943041 1.5294481

C -3.4456148 1.1372321 -0.3857711

H -4.3131702 0.8310385 -0.9800342

H -2.5669470 0.8066202 -0.9547269

C -3.4207405 2.6497617 -0.2550328

H -3.3533204 3.1421858 -1.2280531

H -4.3168207 3.0201236 0.2510228

H -2.5585370 2.9761710 0.3383315

**3 (TPSS)**

Au 0.1870624 9.5921279 0.0000000

Au 0.1387433 7.0670366 1.4910781

Au 0.1387433 7.0670366 -1.4910781

Au 0.1153996 4.5881908 2.9443772

Au 0.1153996 4.5881908 -2.9443772

Au 0.0022797 4.5997189 0.0000000

Au 0.0655694 2.0852000 4.4232456

Au 0.0655694 2.0852000 -4.4232456

Au 0.0027682 2.0955269 -1.4724210

Au 0.0027682 2.0955269 1.4724210

Au -0.0482816 -0.4001087 5.9196464

Au -0.0482816 -0.4001087 -5.9196464

Au -0.0718635 -0.4092256 -2.9689204

Au -0.0469406 -0.4153710 0.0000000

Au -0.0718635 -0.4092256 2.9689204

Au -0.1110387 -2.8998671 7.4022516

Au -0.1110387 -2.8998671 -7.4022516

Au -0.2394359 -2.8589084 -4.4410673

Au -0.1896858 -2.8672319 -1.4771707

Au -0.1896858 -2.8672319 1.4771707

Au -0.2394359 -2.8589084 4.4410673

Au 0.1153996 -5.3038203 -5.8473867

Au 0.1050302 -5.3177094 -2.9377369

Au 0.0923888 -5.3346417 0.0000000

Au 0.1050302 -5.3177094 2.9377369

Au 0.1153996 -5.3038203 5.8473867

C -3.4115350 0.4045849 0.9587935

H -3.3749739 -0.6840655 0.8448582

H -2.5434700 0.7022870 1.5653717

H -4.3113984 0.6616553 1.5306600

C -3.3908079 1.1173222 -0.3958372

H -4.2590721 0.8091433 -0.9930885

H -2.5049672 0.7908643 -0.9632477

C -3.3672063 2.6420386 -0.2600963

H -3.2929267 3.1358405 -1.2349009

H -4.2669912 3.0119014 0.2456576

H -2.5022936 2.9629765 0.3391283

**3 (CAM_B3LYP)**

Au 0.1870624 9.5921279 0.0000000

Au 0.1387433 7.0670366 1.4910781

Au 0.1387433 7.0670366 -1.4910781

Au 0.1153996 4.5881908 2.9443772

Au 0.1153996 4.5881908 -2.9443772

Au 0.0022797 4.5997189 0.0000000

Au 0.0655694 2.0852000 4.4232456

Au 0.0655694 2.0852000 -4.4232456

Au 0.0027682 2.0955269 -1.4724210

Au 0.0027682 2.0955269 1.4724210

Au -0.0482816 -0.4001087 5.9196464

Au -0.0482816 -0.4001087 -5.9196464

Au -0.0718635 -0.4092256 -2.9689204

Au -0.0469406 -0.4153710 0.0000000

Au -0.0718635 -0.4092256 2.9689204

Au -0.1110387 -2.8998671 7.4022516

Au -0.1110387 -2.8998671 -7.4022516

Au -0.2394359 -2.8589084 -4.4410673

Au -0.1896858 -2.8672319 -1.4771707

Au -0.1896858 -2.8672319 1.4771707

Au -0.2394359 -2.8589084 4.4410673

Au 0.1153996 -5.3038203 -5.8473867

Au 0.1050302 -5.3177094 -2.9377369

Au 0.0923888 -5.3346417 0.0000000

Au 0.1050302 -5.3177094 2.9377369

Au 0.1153996 -5.3038203 5.8473867

C -3.4686036 0.4308512 0.9590911

H -3.4439637 -0.6535075 0.8480125

H -2.6056323 0.7179930 1.5647561

H -4.3628630 0.6935441 1.5274043

C -3.4455286 1.1365008 -0.3868898

H -4.3124295 0.8320167 -0.9781086

H -2.5705674 0.8070351 -0.9537795

C -3.4202935 2.6506235 -0.2548917

H -3.3531954 3.1414605 -1.2262673

H -4.3155454 3.0186652 0.2496035

H -2.5613816 2.9759255 0.3369943

**4**

Au 0.1870624 9.5921279 0.0000000

Au 0.1387433 7.0670366 1.4910781

Au 0.1387433 7.0670366 -1.4910781

Au 0.1153996 4.5881908 2.9443772

Au 0.1153996 4.5881908 -2.9443772

Au 0.0022797 4.5997189 0.0000000

Au 0.0655694 2.0852000 4.4232456

Au 0.0655694 2.0852000 -4.4232456

Au 0.0027682 2.0955269 -1.4724210

Au 0.0027682 2.0955269 1.4724210

Au -0.0482816 -0.4001087 5.9196464

Au -0.0482816 -0.4001087 -5.9196464

Au -0.0718635 -0.4092256 -2.9689204

Au -0.0469406 -0.4153710 0.0000000

Au -0.0718635 -0.4092256 2.9689204

Au -0.1110387 -2.8998671 7.4022516

Au -0.1110387 -2.8998671 -7.4022516

Au -0.2394359 -2.8589084 -4.4410673

Au -0.1896858 -2.8672319 -1.4771707

Au -0.1896858 -2.8672319 1.4771707

Au -0.2394359 -2.8589084 4.4410673

Au 0.1153996 -5.3038203 -5.8473867

Au 0.1050302 -5.3177094 -2.9377369

Au 0.0923888 -5.3346417 0.0000000

Au 0.1050302 -5.3177094 2.9377369

Au 0.1153996 -5.3038203 5.8473867

C -3.4646362 0.4184272 0.9567981

H -3.4261156 -0.6671114 0.8429946

H -2.5955504 0.7163071 1.5550387

H -4.3565984 0.6655014 1.5391304

C -3.4697147 1.1264086 -0.3854574

H -4.3521544 0.8265997 -0.9627198

H -2.6044442 0.7993024 -0.9786227

C -3.4356473 2.6401120 -0.2652397

H -4.2744903 2.9779080 0.3546132

H -2.5280128 2.9314633 0.2826100

C -3.4648637 3.3477367 -1.6081576

H -3.3627475 4.4294986 -1.4979986

H -2.6444719 3.0055482 -2.2480311

H -4.3983444 3.1461572 -2.1413471

**5**

Au 0.1870624 9.5921279 0.0000000

Au 0.1387433 7.0670366 1.4910781

Au 0.1387433 7.0670366 -1.4910781

Au 0.1153996 4.5881908 2.9443772

Au 0.1153996 4.5881908 -2.9443772

Au 0.0022797 4.5997189 0.0000000

Au 0.0655694 2.0852000 4.4232456

Au 0.0655694 2.0852000 -4.4232456

Au 0.0027682 2.0955269 -1.4724210

Au 0.0027682 2.0955269 1.4724210

Au -0.0482816 -0.4001087 5.9196464

Au -0.0482816 -0.4001087 -5.9196464

Au -0.0718635 -0.4092256 -2.9689204

Au -0.0469406 -0.4153710 0.0000000

Au -0.0718635 -0.4092256 2.9689204

Au -0.1110387 -2.8998671 7.4022516

Au -0.1110387 -2.8998671 -7.4022516

Au -0.2394359 -2.8589084 -4.4410673

Au -0.1896858 -2.8672319 -1.4771707

Au -0.1896858 -2.8672319 1.4771707

Au -0.2394359 -2.8589084 4.4410673

Au 0.1153996 -5.3038203 -5.8473867

Au 0.1050302 -5.3177094 -2.9377369

Au 0.0923888 -5.3346417 0.0000000

Au 0.1050302 -5.3177094 2.9377369

Au 0.1153996 -5.3038203 5.8473867

C -3.4450894 0.1796037 1.0241058

H -2.5040026 0.4274945 1.5358929

H -4.2414277 0.5192040 1.6962398

C -3.5132097 0.9561824 -0.2796464

H -4.4463489 0.7130110 -0.8042431

H -2.7037016 0.6165022 -0.9424925

C -3.4074762 2.4601506 -0.1001099

H -4.2030921 2.8077699 0.5686660

H -2.4656866 2.6904770 0.4189707

C -3.4600247 3.2261238 -1.4099720

H -3.3135771 4.2976691 -1.2572797

H -2.6780083 2.8838208 -2.0961685

H -4.4198898 3.0822214 -1.9141277

C -3.5358284 -1.3234226 0.8313953

H -3.4021053 -1.8601284 1.7738842

H -4.5026193 -1.6129000 0.4102782

H -2.7626358 -1.6764958 0.1396539

**6**

Au 0.1870624 9.5921279 0.0000000

Au 0.1387433 7.0670366 1.4910781

Au 0.1387433 7.0670366 -1.4910781

Au 0.1153996 4.5881908 2.9443772

Au 0.1153996 4.5881908 -2.9443772

Au 0.0022797 4.5997189 0.0000000

Au 0.0655694 2.0852000 4.4232456

Au 0.0655694 2.0852000 -4.4232456

Au 0.0027682 2.0955269 -1.4724210

Au 0.0027682 2.0955269 1.4724210

Au -0.0482816 -0.4001087 5.9196464

Au -0.0482816 -0.4001087 -5.9196464

Au -0.0718635 -0.4092256 -2.9689204

Au -0.0469406 -0.4153710 0.0000000

Au -0.0718635 -0.4092256 2.9689204

Au -0.1110387 -2.8998671 7.4022516

Au -0.1110387 -2.8998671 -7.4022516

Au -0.2394359 -2.8589084 -4.4410673

Au -0.1896858 -2.8672319 -1.4771707

Au -0.1896858 -2.8672319 1.4771707

Au -0.2394359 -2.8589084 4.4410673

Au 0.1153996 -5.3038203 -5.8473867

Au 0.1050302 -5.3177094 -2.9377369

Au 0.0923888 -5.3346417 0.0000000

Au 0.1050302 -5.3177094 2.9377369

Au 0.1153996 -5.3038203 5.8473867

C -3.5092216 0.2097874 1.0261719

H -2.6017968 0.4625419 1.5954070

H -4.3488711 0.5494057 1.6457828

C -3.5065141 0.9752860 -0.2854305

H -4.4140753 0.7327342 -0.8526681

H -2.6676107 0.6297475 -0.9072287

C -3.4054635 2.4801912 -0.1100639

H -4.2114413 2.8278199 0.5462681

H -2.4718932 2.7147218 0.4215280

C -3.4448092 3.2411524 -1.4233052

H -3.2868239 4.3116770 -1.2751303

H -2.6649903 2.8874557 -2.1062435

H -4.4048324 3.1052649 -1.9294231

C -3.5829266 -1.2978810 0.8566351

H -4.4532271 -1.5535978 0.2412109

H -2.7078508 -1.6365932 0.2842594

C -3.6508622 -2.0428584 2.1780577

H -3.6368216 -3.1255963 2.0329911

H -2.8002914 -1.7872375 2.8180533

H -4.5611836 -1.7876440 2.7281084

**7**

Au 0.1870624 9.5921279 0.0000000

Au 0.1387433 7.0670366 1.4910781

Au 0.1387433 7.0670366 -1.4910781

Au 0.1153996 4.5881908 2.9443772

Au 0.1153996 4.5881908 -2.9443772

Au 0.0022797 4.5997189 0.0000000

Au 0.0655694 2.0852000 4.4232456

Au 0.0655694 2.0852000 -4.4232456

Au 0.0027682 2.0955269 -1.4724210

Au 0.0027682 2.0955269 1.4724210

Au -0.0482816 -0.4001087 5.9196464

Au -0.0482816 -0.4001087 -5.9196464

Au -0.0718635 -0.4092256 -2.9689204

Au -0.0469406 -0.4153710 0.0000000

Au -0.0718635 -0.4092256 2.9689204

Au -0.1110387 -2.8998671 7.4022516

Au -0.1110387 -2.8998671 -7.4022516

Au -0.2394359 -2.8589084 -4.4410673

Au -0.1896858 -2.8672319 -1.4771707

Au -0.1896858 -2.8672319 1.4771707

Au -0.2394359 -2.8589084 4.4410673

Au 0.1153996 -5.3038203 -5.8473867

Au 0.1050302 -5.3177094 -2.9377369

Au 0.0923888 -5.3346417 0.0000000

Au 0.1050302 -5.3177094 2.9377369

Au 0.1153996 -5.3038203 5.8473867

C -3.6384808 -0.8156797 -0.3889537

C -3.5804155 -0.7703013 1.1060060

C -3.6129886 0.5027178 0.3199035

H -2.7464376 -1.1220561 -0.9259979

H -4.5641978 -1.1279256 -0.8562264

H -2.6481623 -1.0459264 1.5878646

H -4.4650724 -1.0529828 1.6631646

H -4.5217198 1.0912303 0.3368882

H -2.7022000 1.0890675 0.2668603

**8**

Au 0.1870624 9.5921279 0.0000000

Au 0.1387433 7.0670366 1.4910781

Au 0.1387433 7.0670366 -1.4910781

Au 0.1153996 4.5881908 2.9443772

Au 0.1153996 4.5881908 -2.9443772

Au 0.0022797 4.5997189 0.0000000

Au 0.0655694 2.0852000 4.4232456

Au 0.0655694 2.0852000 -4.4232456

Au 0.0027682 2.0955269 -1.4724210

Au 0.0027682 2.0955269 1.4724210

Au -0.0482816 -0.4001087 5.9196464

Au -0.0482816 -0.4001087 -5.9196464

Au -0.0718635 -0.4092256 -2.9689204

Au -0.0469406 -0.4153710 0.0000000

Au -0.0718635 -0.4092256 2.9689204

Au -0.1110387 -2.8998671 7.4022516

Au -0.1110387 -2.8998671 -7.4022516

Au -0.2394359 -2.8589084 -4.4410673

Au -0.1896858 -2.8672319 -1.4771707

Au -0.1896858 -2.8672319 1.4771707

Au -0.2394359 -2.8589084 4.4410673

Au 0.1153996 -5.3038203 -5.8473867

Au 0.1050302 -5.3177094 -2.9377369

Au 0.0923888 -5.3346417 0.0000000

Au 0.1050302 -5.3177094 2.9377369

Au 0.1153996 -5.3038203 5.8473867

C -3.5778969 -1.0917400 -0.5730351

C -3.5823166 -1.0763157 0.9708151

C -3.5210858 0.4671403 0.9554063

C -3.5366371 0.4525466 -0.5885352

H -2.6985170 -1.5657772 -1.0185967

H -4.4618844 -1.5356253 -1.0333843

H -2.7157879 -1.5544499 1.4359742

H -4.4793896 -1.4948651 1.4295386

H -2.6082225 0.8811433 1.3935187

H -4.3707325 0.9685976 1.4209661

H -2.6436492 0.8766104 -1.0569729

H -4.4074299 0.9288279 -1.0409822

**8 (TPSS)**

Au 0.1870624 9.5921279 0.0000000

Au 0.1387433 7.0670366 1.4910781

Au 0.1387433 7.0670366 -1.4910781

Au 0.1153996 4.5881908 2.9443772

Au 0.1153996 4.5881908 -2.9443772

Au 0.0022797 4.5997189 0.0000000

Au 0.0655694 2.0852000 4.4232456

Au 0.0655694 2.0852000 -4.4232456

Au 0.0027682 2.0955269 -1.4724210

Au 0.0027682 2.0955269 1.4724210

Au -0.0482816 -0.4001087 5.9196464

Au -0.0482816 -0.4001087 -5.9196464

Au -0.0718635 -0.4092256 -2.9689204

Au -0.0469406 -0.4153710 0.0000000

Au -0.0718635 -0.4092256 2.9689204

Au -0.1110387 -2.8998671 7.4022516

Au -0.1110387 -2.8998671 -7.4022516

Au -0.2394359 -2.8589084 -4.4410673

Au -0.1896858 -2.8672319 -1.4771707

Au -0.1896858 -2.8672319 1.4771707

Au -0.2394359 -2.8589084 4.4410673

Au 0.1153996 -5.3038203 -5.8473867

Au 0.1050302 -5.3177094 -2.9377369

Au 0.0923888 -5.3346417 0.0000000

Au 0.1050302 -5.3177094 2.9377369

Au 0.1153996 -5.3038203 5.8473867

C -3.5079683 -1.0972490 -0.5869162

C -3.5046874 -1.0814129 0.9684953

C -3.4303960 0.4737315 0.9521641

C -3.4687030 0.4595516 -0.6032784

H -2.6205111 -1.5660073 -1.0328734

H -4.3916959 -1.5474407 -1.0480437

H -2.6331592 -1.5657376 1.4283582

H -4.4040733 -1.4939418 1.4345181

H -2.4990893 0.8745970 1.3727432

H -4.2666266 0.9870112 1.4350406

H -2.5777218 0.8827865 -1.0842947

H -4.3488623 0.9362531 -1.0437552

**8 (CAM-B3LYP)**

Au 0.1870624 9.5921279 0.0000000

Au 0.1387433 7.0670366 1.4910781

Au 0.1387433 7.0670366 -1.4910781

Au 0.1153996 4.5881908 2.9443772

Au 0.1153996 4.5881908 -2.9443772

Au 0.0022797 4.5997189 0.0000000

Au 0.0655694 2.0852000 4.4232456

Au 0.0655694 2.0852000 -4.4232456

Au 0.0027682 2.0955269 -1.4724210

Au 0.0027682 2.0955269 1.4724210

Au -0.0482816 -0.4001087 5.9196464

Au -0.0482816 -0.4001087 -5.9196464

Au -0.0718635 -0.4092256 -2.9689204

Au -0.0469406 -0.4153710 0.0000000

Au -0.0718635 -0.4092256 2.9689204

Au -0.1110387 -2.8998671 7.4022516

Au -0.1110387 -2.8998671 -7.4022516

Au -0.2394359 -2.8589084 -4.4410673

Au -0.1896858 -2.8672319 -1.4771707

Au -0.1896858 -2.8672319 1.4771707

Au -0.2394359 -2.8589084 4.4410673

Au 0.1153996 -5.3038203 -5.8473867

Au 0.1050302 -5.3177094 -2.9377369

Au 0.0923888 -5.3346417 0.0000000

Au 0.1050302 -5.3177094 2.9377369

Au 0.1153996 -5.3038203 5.8473867

C -3.5738175 -1.0456780 -0.6120631

C -3.7326657 -1.0420198 0.9245443

C -3.5002231 0.4855340 0.9320551

C -3.6048884 0.4989124 -0.6088093

H -2.6151303 -1.4473621 -0.9391191

H -4.3551359 -1.5460754 -1.1816976

H -3.0106912 -1.6356968 1.4851840

H -4.7328644 -1.3239052 1.2505897

H -2.5079276 0.7565813 1.2922669

H -4.2278827 1.0875234 1.4736294

H -2.7936496 0.9951377 -1.1425790

H -4.5479671 0.9080377 -0.9684954

**9**

Au 0.1870624 9.5921279 0.0000000

Au 0.1387433 7.0670366 1.4910781

Au 0.1387433 7.0670366 -1.4910781

Au 0.1153996 4.5881908 2.9443772

Au 0.1153996 4.5881908 -2.9443772

Au 0.0022797 4.5997189 0.0000000

Au 0.0655694 2.0852000 4.4232456

Au 0.0655694 2.0852000 -4.4232456

Au 0.0027682 2.0955269 -1.4724210

Au 0.0027682 2.0955269 1.4724210

Au -0.0482816 -0.4001087 5.9196464

Au -0.0482816 -0.4001087 -5.9196464

Au -0.0718635 -0.4092256 -2.9689204

Au -0.0469406 -0.4153710 0.0000000

Au -0.0718635 -0.4092256 2.9689204

Au -0.1110387 -2.8998671 7.4022516

Au -0.1110387 -2.8998671 -7.4022516

Au -0.2394359 -2.8589084 -4.4410673

Au -0.1896858 -2.8672319 -1.4771707

Au -0.1896858 -2.8672319 1.4771707

Au -0.2394359 -2.8589084 4.4410673

Au 0.1153996 -5.3038203 -5.8473867

Au 0.1050302 -5.3177094 -2.9377369

Au 0.0923888 -5.3346417 0.0000000

Au 0.1050302 -5.3177094 2.9377369

Au 0.1153996 -5.3038203 5.8473867

C -3.5802869 -1.2600917 0.6851131

C -4.1148768 -1.2148776 -0.7440409

C -3.4668377 0.0388139 -1.3171398

C -3.5342351 1.0484880 -0.1723139

C -3.4625918 0.2107645 1.1217078

H -4.1973949 -1.8571968 1.3594685

H -2.5865959 -1.7255918 0.6788612

H -5.2045801 -1.1006583 -0.7375114

H -3.8823467 -2.1169287 -1.3151114

H -2.4175547 -0.1811680 -1.5577921

H -3.9333076 0.3978071 -2.2367185

H -2.7351479 1.7937492 -0.2309257

H -4.4789413 1.5970819 -0.2154867

H -2.5253936 0.3843585 1.6616021

H -4.2636454 0.4881322 1.8097228

**10**

Au 0.1870624 9.5921279 0.0000000

Au 0.1387433 7.0670366 1.4910781

Au 0.1387433 7.0670366 -1.4910781

Au 0.1153996 4.5881908 2.9443772

Au 0.1153996 4.5881908 -2.9443772

Au 0.0022797 4.5997189 0.0000000

Au 0.0655694 2.0852000 4.4232456

Au 0.0655694 2.0852000 -4.4232456

Au 0.0027682 2.0955269 -1.4724210

Au 0.0027682 2.0955269 1.4724210

Au -0.0482816 -0.4001087 5.9196464

Au -0.0482816 -0.4001087 -5.9196464

Au -0.0718635 -0.4092256 -2.9689204

Au -0.0469406 -0.4153710 0.0000000

Au -0.0718635 -0.4092256 2.9689204

Au -0.1110387 -2.8998671 7.4022516

Au -0.1110387 -2.8998671 -7.4022516

Au -0.2394359 -2.8589084 -4.4410673

Au -0.1896858 -2.8672319 -1.4771707

Au -0.1896858 -2.8672319 1.4771707

Au -0.2394359 -2.8589084 4.4410673

Au 0.1153996 -5.3038203 -5.8473867

Au 0.1050302 -5.3177094 -2.9377369

Au 0.0923888 -5.3346417 0.0000000

Au 0.1050302 -5.3177094 2.9377369

Au 0.1153996 -5.3038203 5.8473867

C -3.9634638 -1.6247703 0.1469691

C -3.5729032 -0.9924178 -1.1817753

C -4.0639559 0.4438444 -1.2972552

C -3.6012796 1.2859647 -0.1158306

C -4.0040502 0.6630905 1.2138263

C -3.5092106 -0.7719342 1.3233621

H -3.7133254 0.8854315 -2.2354274

H -2.4764655 -0.9955029 -1.2649990

H -3.9391680 -1.5952542 -2.0182419

H -5.0540555 -1.7417374 0.1853798

H -3.5374737 -2.6305686 0.2248714

H -2.5064968 1.3695769 -0.1478610

H -3.9875170 2.3063833 -0.1958566

H -3.6126922 1.2596452 2.0441028

H -5.0978413 0.6771336 1.3042434

H -2.4095540 -0.7618870 1.3437743

H -3.8224483 -1.2207025 2.2708061

H -5.1609216 0.4494578 -1.3373082

**11**

Au 0.1870624 9.5921279 0.0000000

Au 0.1387433 7.0670366 1.4910781

Au 0.1387433 7.0670366 -1.4910781

Au 0.1153996 4.5881908 2.9443772

Au 0.1153996 4.5881908 -2.9443772

Au 0.0022797 4.5997189 0.0000000

Au 0.0655694 2.0852000 4.4232456

Au 0.0655694 2.0852000 -4.4232456

Au 0.0027682 2.0955269 -1.4724210

Au 0.0027682 2.0955269 1.4724210

Au -0.0482816 -0.4001087 5.9196464

Au -0.0482816 -0.4001087 -5.9196464

Au -0.0718635 -0.4092256 -2.9689204

Au -0.0469406 -0.4153710 0.0000000

Au -0.0718635 -0.4092256 2.9689204

Au -0.1110387 -2.8998671 7.4022516

Au -0.1110387 -2.8998671 -7.4022516

Au -0.2394359 -2.8589084 -4.4410673

Au -0.1896858 -2.8672319 -1.4771707

Au -0.1896858 -2.8672319 1.4771707

Au -0.2394359 -2.8589084 4.4410673

Au 0.1153996 -5.3038203 -5.8473867

Au 0.1050302 -5.3177094 -2.9377369

Au 0.0923888 -5.3346417 0.0000000

Au 0.1050302 -5.3177094 2.9377369

Au 0.1153996 -5.3038203 5.8473867

C -3.6504853 -0.7673233 -1.2843690

H -4.0022423 -1.8003793 -1.3454613

H -3.9963227 -0.2352069 -2.1743365

H -2.5550332 -0.7940296 -1.3255199

C -4.1167664 -0.0873277 -0.0052728

H -5.2147568 -0.0834278 -0.0043320

C -3.6408676 1.3570442 0.0460012

H -3.9827485 1.8625465 0.9528470

H -2.5457674 1.3972163 0.0412830

H -3.9925696 1.9287511 -0.8166204

C -3.6490096 -0.8570711 1.2212807

H -3.9987735 -0.3937750 2.1474531

H -3.9956758 -1.8934677 1.2052687

H -2.5535758 -0.8808831 1.2628863

**12**

Au 0.1870624 9.5921279 0.0000000

Au 0.1387433 7.0670366 1.4910781

Au 0.1387433 7.0670366 -1.4910781

Au 0.1153996 4.5881908 2.9443772

Au 0.1153996 4.5881908 -2.9443772

Au 0.0022797 4.5997189 0.0000000

Au 0.0655694 2.0852000 4.4232456

Au 0.0655694 2.0852000 -4.4232456

Au 0.0027682 2.0955269 -1.4724210

Au 0.0027682 2.0955269 1.4724210

Au -0.0482816 -0.4001087 5.9196464

Au -0.0482816 -0.4001087 -5.9196464

Au -0.0718635 -0.4092256 -2.9689204

Au -0.0469406 -0.4153710 0.0000000

Au -0.0718635 -0.4092256 2.9689204

Au -0.1110387 -2.8998671 7.4022516

Au -0.1110387 -2.8998671 -7.4022516

Au -0.2394359 -2.8589084 -4.4410673

Au -0.1896858 -2.8672319 -1.4771707

Au -0.1896858 -2.8672319 1.4771707

Au -0.2394359 -2.8589084 4.4410673

Au 0.1153996 -5.3038203 -5.8473867

Au 0.1050302 -5.3177094 -2.9377369

Au 0.0923888 -5.3346417 0.0000000

Au 0.1050302 -5.3177094 2.9377369

Au 0.1153996 -5.3038203 5.8473867

C -3.4743476 0.4085874 1.4182052

H -2.9363045 0.7527669 2.3064049

H -3.0742822 0.9615318 0.5665174

H -4.5271427 0.6842549 1.5399775

C -3.3193637 -1.0965706 1.2549446

H -2.2521595 -1.2906011 1.0442078

C -4.0845414 -1.6607871 0.0497293

H -5.1572933 -1.5082298 0.2413409

C -3.6510810 -1.8035825 2.5607512

H -3.3977335 -2.8645471 2.5352233

H -3.0895731 -1.3611818 3.3886837

H -4.7172867 -1.7124246 2.7934410

C -3.8403706 -3.1553903 -0.1234685

H -4.2030226 -3.7417758 0.7216086

H -4.3333097 -3.5292863 -1.0239175

H -2.7674002 -3.3587893 -0.2308640

C -3.7244954 -0.9364949 -1.2419884

H -3.9928706 0.1202324 -1.2214831

H -2.6456600 -1.0024637 -1.4294679

H -4.2285560 -1.3917829 -2.0977892

**12 (TPSS)**

Au 0.1870624 9.5921279 0.0000000

Au 0.1387433 7.0670366 1.4910781

Au 0.1387433 7.0670366 -1.4910781

Au 0.1153996 4.5881908 2.9443772

Au 0.1153996 4.5881908 -2.9443772

Au 0.0022797 4.5997189 0.0000000

Au 0.0655694 2.0852000 4.4232456

Au 0.0655694 2.0852000 -4.4232456

Au 0.0027682 2.0955269 -1.4724210

Au 0.0027682 2.0955269 1.4724210

Au -0.0482816 -0.4001087 5.9196464

Au -0.0482816 -0.4001087 -5.9196464

Au -0.0718635 -0.4092256 -2.9689204

Au -0.0469406 -0.4153710 0.0000000

Au -0.0718635 -0.4092256 2.9689204

Au -0.1110387 -2.8998671 7.4022516

Au -0.1110387 -2.8998671 -7.4022516

Au -0.2394359 -2.8589084 -4.4410673

Au -0.1896858 -2.8672319 -1.4771707

Au -0.1896858 -2.8672319 1.4771707

Au -0.2394359 -2.8589084 4.4410673

Au 0.1153996 -5.3038203 -5.8473867

Au 0.1050302 -5.3177094 -2.9377369

Au 0.0923888 -5.3346417 0.0000000

Au 0.1050302 -5.3177094 2.9377369

Au 0.1153996 -5.3038203 5.8473867

C -3.3915912 0.4295117 1.4208650

H -2.8464242 0.7712934 2.3091833

H -2.9860721 0.9749654 0.5631769

H -4.4453993 0.7114432 1.5429539

C -3.2427664 -1.0887658 1.2605073

H -2.1700196 -1.2851592 1.0431618

C -4.0092049 -1.6606540 0.0448067

H -5.0858394 -1.5086826 0.2265248

C -3.5644437 -1.8003270 2.5803002

H -3.3168401 -2.8651997 2.5462589

H -2.9824728 -1.3604073 3.3996421

H -4.6285137 -1.7005531 2.8302568

C -3.7552418 -3.1665539 -0.1227843

H -4.1195908 -3.7511097 0.7263109

H -4.2404551 -3.5459808 -1.0283998

H -2.6754946 -3.3599402 -0.2217026

C -3.6292494 -0.9331694 -1.2539219

H -3.8964700 0.1266904 -1.2371313

H -2.5428335 -1.0001654 -1.4193800

H -4.1191365 -1.3934383 -2.1187399

**12 (CAM-B3LYP)**

Au 0.1870624 9.5921279 0.0000000

Au 0.1387433 7.0670366 1.4910781

Au 0.1387433 7.0670366 -1.4910781

Au 0.1153996 4.5881908 2.9443772

Au 0.1153996 4.5881908 -2.9443772

Au 0.0022797 4.5997189 0.0000000

Au 0.0655694 2.0852000 4.4232456

Au 0.0655694 2.0852000 -4.4232456

Au 0.0027682 2.0955269 -1.4724210

Au 0.0027682 2.0955269 1.4724210

Au -0.0482816 -0.4001087 5.9196464

Au -0.0482816 -0.4001087 -5.9196464

Au -0.0718635 -0.4092256 -2.9689204

Au -0.0469406 -0.4153710 0.0000000

Au -0.0718635 -0.4092256 2.9689204

Au -0.1110387 -2.8998671 7.4022516

Au -0.1110387 -2.8998671 -7.4022516

Au -0.2394359 -2.8589084 -4.4410673

Au -0.1896858 -2.8672319 -1.4771707

Au -0.1896858 -2.8672319 1.4771707

Au -0.2394359 -2.8589084 4.4410673

Au 0.1153996 -5.3038203 -5.8473867

Au 0.1050302 -5.3177094 -2.9377369

Au 0.0923888 -5.3346417 0.0000000

Au 0.1050302 -5.3177094 2.9377369

Au 0.1153996 -5.3038203 5.8473867

C -3.4961278 0.4266957 1.4170857

H -2.9550324 0.7690953 2.3009774

H -3.1012142 0.9791611 0.5660198

H -4.5467829 0.7003774 1.5426793

C -3.3429414 -1.0804798 1.2503543

H -2.2857438 -1.2751118 1.0304814

C -4.1271118 -1.6411717 0.0541468

H -5.1937062 -1.4805374 0.2559504

C -3.6647936 -1.7868982 2.5615815

H -3.4100389 -2.8450228 2.5355262

H -3.0992031 -1.3440515 3.3829848

H -4.7278093 -1.6975058 2.7995228

C -3.8966307 -3.1402206 -0.1201615

H -4.2627999 -3.7204598 0.7248831

H -4.3983854 -3.5074377 -1.0161053

H -2.8298140 -3.3550838 -0.2314369

C -3.7758425 -0.9218392 -1.2456027

H -4.0355639 0.1348045 -1.2222635

H -2.7041424 -0.9978918 -1.4479834

H -4.2976883 -1.3725683 -2.0905294

**13**

Au 0.1870624 9.5921279 0.0000000

Au 0.1387433 7.0670366 1.4910781

Au 0.1387433 7.0670366 -1.4910781

Au 0.1153996 4.5881908 2.9443772

Au 0.1153996 4.5881908 -2.9443772

Au 0.0022797 4.5997189 0.0000000

Au 0.0655694 2.0852000 4.4232456

Au 0.0655694 2.0852000 -4.4232456

Au 0.0027682 2.0955269 -1.4724210

Au 0.0027682 2.0955269 1.4724210

Au -0.0482816 -0.4001087 5.9196464

Au -0.0482816 -0.4001087 -5.9196464

Au -0.0718635 -0.4092256 -2.9689204

Au -0.0469406 -0.4153710 0.0000000

Au -0.0718635 -0.4092256 2.9689204

Au -0.1110387 -2.8998671 7.4022516

Au -0.1110387 -2.8998671 -7.4022516

Au -0.2394359 -2.8589084 -4.4410673

Au -0.1896858 -2.8672319 -1.4771707

Au -0.1896858 -2.8672319 1.4771707

Au -0.2394359 -2.8589084 4.4410673

Au 0.1153996 -5.3038203 -5.8473867

Au 0.1050302 -5.3177094 -2.9377369

Au 0.0923888 -5.3346417 0.0000000

Au 0.1050302 -5.3177094 2.9377369

Au 0.1153996 -5.3038203 5.8473867

C -3.6784198 -3.5004072 -0.4260630

H -4.2278496 -4.4147978 -0.1887353

H -3.7008583 -3.3760658 -1.5086061

H -2.6360658 -3.6676989 -0.1287716

C -4.2647845 -2.3136980 0.3312781

H -5.2862752 -2.1470307 -0.0420330

C -3.4889848 -1.0046553 0.0749619

H -2.4627698 -1.1734418 0.4481370

C -4.3615765 -2.6881573 1.8056428

H -4.8905783 -1.9409665 2.3978509

H -4.8959897 -3.6343358 1.9197332

H -3.3625255 -2.8221028 2.2369781

C -4.0657413 0.1954501 0.8748605

H -5.1320555 -0.0115254 1.0422225

C -3.3804576 -0.7306455 -1.4199531

H -2.8580638 -1.5379529 -1.9347464

H -4.3672509 -0.6262897 -1.8826152

H -2.8179791 0.1824248 -1.6236102

C -3.3897401 0.3576362 2.2328687

H -3.9075606 1.0963551 2.8503142

H -3.3153802 -0.5686585 2.7993581

H -2.3685773 0.7307128 2.0840946

C -3.9750922 1.5401287 0.1587299

H -2.9312401 1.8074168 -0.0421518

H -4.5156898 1.5569338 -0.7881181

H -4.3910550 2.3264874 0.7938507

**14**

Au 0.1870624 9.5921279 0.0000000

Au 0.1387433 7.0670366 1.4910781

Au 0.1387433 7.0670366 -1.4910781

Au 0.1153996 4.5881908 2.9443772

Au 0.1153996 4.5881908 -2.9443772

Au 0.0022797 4.5997189 0.0000000

Au 0.0655694 2.0852000 4.4232456

Au 0.0655694 2.0852000 -4.4232456

Au 0.0027682 2.0955269 -1.4724210

Au 0.0027682 2.0955269 1.4724210

Au -0.0482816 -0.4001087 5.9196464

Au -0.0482816 -0.4001087 -5.9196464

Au -0.0718635 -0.4092256 -2.9689204

Au -0.0469406 -0.4153710 0.0000000

Au -0.0718635 -0.4092256 2.9689204

Au -0.1110387 -2.8998671 7.4022516

Au -0.1110387 -2.8998671 -7.4022516

Au -0.2394359 -2.8589084 -4.4410673

Au -0.1896858 -2.8672319 -1.4771707

Au -0.1896858 -2.8672319 1.4771707

Au -0.2394359 -2.8589084 4.4410673

Au 0.1153996 -5.3038203 -5.8473867

Au 0.1050302 -5.3177094 -2.9377369

Au 0.0923888 -5.3346417 0.0000000

Au 0.1050302 -5.3177094 2.9377369

Au 0.1153996 -5.3038203 5.8473867

C -4.0082506 2.6859204 1.1965401

H -3.1266940 3.1561419 1.6434223

H -4.2727073 3.2642432 0.3087446

H -4.8292695 2.7768312 1.9146674

C -3.7110320 1.2305429 0.8687989

H -2.8598308 1.2251073 0.1766549

C -4.8790713 0.4469328 0.2196137

H -5.4062163 -0.0188426 1.0619598

C -3.2756822 0.4984228 2.1306821

H -2.9917929 -0.5377577 1.9263642

H -2.4222191 0.9951853 2.6020227

H -4.0866426 0.4822281 2.8664875

C -4.3635205 -0.7317598 -0.6495553

H -3.3587524 -0.9911725 -0.2808997

C -5.9380981 1.2927394 -0.4800836

H -6.4116804 1.9810147 0.2220853

H -5.5443986 1.8885837 -1.3050673

H -6.7270431 0.6528454 -0.8839665

C -5.2498942 -1.9552115 -0.4389833

H -6.2593174 -1.7821929 -0.8266137

H -4.8526732 -2.8443506 -0.9295750

H -5.3388576 -2.1827409 0.6258504

C -4.1989750 -0.4042732 -2.1457031

H -5.1876575 -0.1382107 -2.5416812

C -3.6952662 -1.6085651 -2.9362820

H -4.4279326 -2.4144729 -2.9906407

H -3.4428023 -1.3220145 -3.9608960

H -2.7837492 -2.0141666 -2.4835441

C -3.2628561 0.7692953 -2.3932877

H -3.5882061 1.6986266 -1.9265225

H -2.2681813 0.5415229 -1.9877392

H -3.1370852 0.9544754 -3.4629355

**15**

C -2.4145829 7.0642840 -0.2504966

C -2.4530977 5.6439310 -0.2256318

C -1.2223392 4.9367242 -0.1521745

C 0.0051579 5.6455466 -0.1056570

C 0.0064359 7.0558788 -0.1321203

C -1.2328199 7.7393022 -0.2055109

C 1.2313787 4.9367003 -0.0326416

C 2.4634174 5.6439573 0.0142562

C 2.4274908 7.0643013 -0.0141219

C 1.2469491 7.7393159 -0.0844082

C -1.2250196 3.5264963 -0.1258101

C 0.0025253 2.8166639 -0.0527674

C 1.2314804 3.5265307 -0.0062618

C 2.4515956 2.8211523 0.0663837

C 3.6860306 3.5370925 0.1132960

C 3.6617124 4.9280612 0.0861554

C -3.6526957 4.9280558 -0.2707400

C -3.6795473 3.5370934 -0.2457796

C -2.4464427 2.8211739 -0.1720357

C -2.4498365 1.4106976 -0.1457027

C -1.2259887 0.7035814 -0.0728565

C 0.0013792 1.4137069 -0.0264984

C 1.2271683 0.7035278 0.0464582

C 2.4524715 1.4106924 0.0928548

C -1.2271728 -0.7036200 -0.0464880

C -0.0012606 -1.4136531 0.0264774

C 1.2258367 -0.7035899 0.0728311

C 2.4498735 -1.4107692 0.1457164

C 3.6736847 -0.6996933 0.1920056

C 3.6749612 0.6996338 0.1658077

C -3.6736920 0.6996292 -0.1919993

C -3.6749836 -0.6996413 -0.1658134

C -2.4524202 -1.4107359 -0.0928764

C 4.9116874 1.4276636 0.2126344

C 4.8957776 2.7989336 0.1862468

C -4.8906596 2.7989485 -0.2910803

C -4.9090654 1.4276964 -0.2661132

C -2.4516102 -2.8211800 -0.0664046

C -1.2314557 -3.5265000 0.0062456

C -0.0026153 -2.8167790 0.0527610

C 1.2250240 -3.5265342 0.1258389

C 2.4464325 -2.8211842 0.1720618

C -1.2313555 -4.9367802 0.0326490

C -0.0051586 -5.6455808 0.1056720

C 1.2223599 -4.9367626 0.1522028

C -0.0064357 -7.0559236 0.1321307

C 1.2328105 -7.7393529 0.2055147

C 2.4146113 -7.0642730 0.2504995

C 2.4531249 -5.6439350 0.2256563

C -2.4634369 -5.6439279 -0.0142611

C -2.4275073 -7.0642964 0.0141225

C -1.2469529 -7.7393530 0.0844100

C 3.6526818 -4.9280304 0.2707483

C 3.6795768 -3.5371182 0.2458092

C -3.6860301 -3.5371134 -0.1133101

C -3.6616902 -4.9280324 -0.0861588

C 4.8906285 -2.7989372 0.2910856

C 4.9090855 -1.4276937 0.2661231

C -4.9116949 -1.4276895 -0.2126351

C -4.8957579 -2.7989156 -0.1862484

C 6.1331076 -0.6735473 0.3118940

C 6.1343742 0.6735801 0.2866366

C -6.1331436 0.6736397 -0.3118964

C -6.1343532 -0.6735793 -0.2866332

H 7.0701237 1.2215798 0.3223169

H 7.0679168 -1.2213858 0.3681048

H 5.8282842 -3.3432307 0.3472588

H 4.5896895 -5.4732687 0.3268734

H 3.3518905 -7.6070488 0.3065010

H 1.2261674 -8.8240195 0.2255898

H -1.2423000 -8.8240309 0.1050292

H -3.3658067 -7.6070639 -0.0214469

H -4.5997092 -5.4732888 -0.1218510

H -5.8344072 -3.3432558 -0.2220383

H -7.0701429 -1.2214123 -0.3223188

H -7.0678864 1.2215548 -0.3681105

H -5.8282879 3.3433562 -0.3472726

H -4.5897087 5.4733075 -0.3268962

H -3.3519273 7.6070100 -0.3065168

H -1.2261066 8.8240017 -0.2255927

H 1.2422388 8.8240145 -0.1050374

H 3.3658424 7.6070379 0.0214517

H 4.5997176 5.4733289 0.1218596

H 5.8343926 3.3433628 0.2220440

C -0.4149299 1.0947000 3.3832981

H -0.4055247 1.1722397 4.4698884

H 0.1076164 1.9470898 2.9510051

H 0.0828939 0.1747721 3.0773751

H -1.4440467 1.0835242 3.0254387

**16**

C -2.4145829 7.0642840 -0.2504966

C -2.4530977 5.6439310 -0.2256318

C -1.2223392 4.9367242 -0.1521745

C 0.0051579 5.6455466 -0.1056570

C 0.0064359 7.0558788 -0.1321203

C -1.2328199 7.7393022 -0.2055109

C 1.2313787 4.9367003 -0.0326416

C 2.4634174 5.6439573 0.0142562

C 2.4274908 7.0643013 -0.0141219

C 1.2469491 7.7393159 -0.0844082

C -1.2250196 3.5264963 -0.1258101

C 0.0025253 2.8166639 -0.0527674

C 1.2314804 3.5265307 -0.0062618

C 2.4515956 2.8211523 0.0663837

C 3.6860306 3.5370925 0.1132960

C 3.6617124 4.9280612 0.0861554

C -3.6526957 4.9280558 -0.2707400

C -3.6795473 3.5370934 -0.2457796

C -2.4464427 2.8211739 -0.1720357

C -2.4498365 1.4106976 -0.1457027

C -1.2259887 0.7035814 -0.0728565

C 0.0013792 1.4137069 -0.0264984

C 1.2271683 0.7035278 0.0464582

C 2.4524715 1.4106924 0.0928548

C -1.2271728 -0.7036200 -0.0464880

C -0.0012606 -1.4136531 0.0264774

C 1.2258367 -0.7035899 0.0728311

C 2.4498735 -1.4107692 0.1457164

C 3.6736847 -0.6996933 0.1920056

C 3.6749612 0.6996338 0.1658077

C -3.6736920 0.6996292 -0.1919993

C -3.6749836 -0.6996413 -0.1658134

C -2.4524202 -1.4107359 -0.0928764

C 4.9116874 1.4276636 0.2126344

C 4.8957776 2.7989336 0.1862468

C -4.8906596 2.7989485 -0.2910803

C -4.9090654 1.4276964 -0.2661132

C -2.4516102 -2.8211800 -0.0664046

C -1.2314557 -3.5265000 0.0062456

C -0.0026153 -2.8167790 0.0527610

C 1.2250240 -3.5265342 0.1258389

C 2.4464325 -2.8211842 0.1720618

C -1.2313555 -4.9367802 0.0326490

C -0.0051586 -5.6455808 0.1056720

C 1.2223599 -4.9367626 0.1522028

C -0.0064357 -7.0559236 0.1321307

C 1.2328105 -7.7393529 0.2055147

C 2.4146113 -7.0642730 0.2504995

C 2.4531249 -5.6439350 0.2256563

C -2.4634369 -5.6439279 -0.0142611

C -2.4275073 -7.0642964 0.0141225

C -1.2469529 -7.7393530 0.0844100

C 3.6526818 -4.9280304 0.2707483

C 3.6795768 -3.5371182 0.2458092

C -3.6860301 -3.5371134 -0.1133101

C -3.6616902 -4.9280324 -0.0861588

C 4.8906285 -2.7989372 0.2910856

C 4.9090855 -1.4276937 0.2661231

C -4.9116949 -1.4276895 -0.2126351

C -4.8957579 -2.7989156 -0.1862484

C 6.1331076 -0.6735473 0.3118940

C 6.1343742 0.6735801 0.2866366

C -6.1331436 0.6736397 -0.3118964

C -6.1343532 -0.6735793 -0.2866332

H 7.0701237 1.2215798 0.3223169

H 7.0679168 -1.2213858 0.3681048

H 5.8282842 -3.3432307 0.3472588

H 4.5896895 -5.4732687 0.3268734

H 3.3518905 -7.6070488 0.3065010

H 1.2261674 -8.8240195 0.2255898

H -1.2423000 -8.8240309 0.1050292

H -3.3658067 -7.6070639 -0.0214469

H -4.5997092 -5.4732888 -0.1218510

H -5.8344072 -3.3432558 -0.2220383

H -7.0701429 -1.2214123 -0.3223188

H -7.0678864 1.2215548 -0.3681105

H -5.8282879 3.3433562 -0.3472726

H -4.5897087 5.4733075 -0.3268962

H -3.3519273 7.6070100 -0.3065168

H -1.2261066 8.8240017 -0.2255927

H 1.2422388 8.8240145 -0.1050374

H 3.3658424 7.6070379 0.0214517

H 4.5997176 5.4733289 0.1218596

H 5.8343926 3.3433628 0.2220440

C -1.6954600 1.1554163 3.3516202

H -2.1148584 1.4729370 4.3095020

C -0.4795284 0.2665030 3.5451525

H -0.0588538 -0.0455999 2.5870923

H 0.3102513 0.7835705 4.0954773

H -2.4842462 0.6373018 2.8010553

H -1.4440443 2.0562910 2.7865857

H -0.7316480 -0.6395224 4.1016524

**17**

C -2.4145829 7.0642840 -0.2504966

C -2.4530977 5.6439310 -0.2256318

C -1.2223392 4.9367242 -0.1521745

C 0.0051579 5.6455466 -0.1056570

C 0.0064359 7.0558788 -0.1321203

C -1.2328199 7.7393022 -0.2055109

C 1.2313787 4.9367003 -0.0326416

C 2.4634174 5.6439573 0.0142562

C 2.4274908 7.0643013 -0.0141219

C 1.2469491 7.7393159 -0.0844082

C -1.2250196 3.5264963 -0.1258101

C 0.0025253 2.8166639 -0.0527674

C 1.2314804 3.5265307 -0.0062618

C 2.4515956 2.8211523 0.0663837

C 3.6860306 3.5370925 0.1132960

C 3.6617124 4.9280612 0.0861554

C -3.6526957 4.9280558 -0.2707400

C -3.6795473 3.5370934 -0.2457796

C -2.4464427 2.8211739 -0.1720357

C -2.4498365 1.4106976 -0.1457027

C -1.2259887 0.7035814 -0.0728565

C 0.0013792 1.4137069 -0.0264984

C 1.2271683 0.7035278 0.0464582

C 2.4524715 1.4106924 0.0928548

C -1.2271728 -0.7036200 -0.0464880

C -0.0012606 -1.4136531 0.0264774

C 1.2258367 -0.7035899 0.0728311

C 2.4498735 -1.4107692 0.1457164

C 3.6736847 -0.6996933 0.1920056

C 3.6749612 0.6996338 0.1658077

C -3.6736920 0.6996292 -0.1919993

C -3.6749836 -0.6996413 -0.1658134

C -2.4524202 -1.4107359 -0.0928764

C 4.9116874 1.4276636 0.2126344

C 4.8957776 2.7989336 0.1862468

C -4.8906596 2.7989485 -0.2910803

C -4.9090654 1.4276964 -0.2661132

C -2.4516102 -2.8211800 -0.0664046

C -1.2314557 -3.5265000 0.0062456

C -0.0026153 -2.8167790 0.0527610

C 1.2250240 -3.5265342 0.1258389

C 2.4464325 -2.8211842 0.1720618

C -1.2313555 -4.9367802 0.0326490

C -0.0051586 -5.6455808 0.1056720

C 1.2223599 -4.9367626 0.1522028

C -0.0064357 -7.0559236 0.1321307

C 1.2328105 -7.7393529 0.2055147

C 2.4146113 -7.0642730 0.2504995

C 2.4531249 -5.6439350 0.2256563

C -2.4634369 -5.6439279 -0.0142611

C -2.4275073 -7.0642964 0.0141225

C -1.2469529 -7.7393530 0.0844100

C 3.6526818 -4.9280304 0.2707483

C 3.6795768 -3.5371182 0.2458092

C -3.6860301 -3.5371134 -0.1133101

C -3.6616902 -4.9280324 -0.0861588

C 4.8906285 -2.7989372 0.2910856

C 4.9090855 -1.4276937 0.2661231

C -4.9116949 -1.4276895 -0.2126351

C -4.8957579 -2.7989156 -0.1862484

C 6.1331076 -0.6735473 0.3118940

C 6.1343742 0.6735801 0.2866366

C -6.1331436 0.6736397 -0.3118964

C -6.1343532 -0.6735793 -0.2866332

H 7.0701237 1.2215798 0.3223169

H 7.0679168 -1.2213858 0.3681048

H 5.8282842 -3.3432307 0.3472588

H 4.5896895 -5.4732687 0.3268734

H 3.3518905 -7.6070488 0.3065010

H 1.2261674 -8.8240195 0.2255898

H -1.2423000 -8.8240309 0.1050292

H -3.3658067 -7.6070639 -0.0214469

H -4.5997092 -5.4732888 -0.1218510

H -5.8344072 -3.3432558 -0.2220383

H -7.0701429 -1.2214123 -0.3223188

H -7.0678864 1.2215548 -0.3681105

H -5.8282879 3.3433562 -0.3472726

H -4.5897087 5.4733075 -0.3268962

H -3.3519273 7.6070100 -0.3065168

H -1.2261066 8.8240017 -0.2255927

H 1.2422388 8.8240145 -0.1050374

H 3.3658424 7.6070379 0.0214517

H 4.5997176 5.4733289 0.1218596

H 5.8343926 3.3433628 0.2220440

C -0.9977173 0.9092391 3.6110540

H -1.5679048 0.7717425 4.5343009

C 0.2504261 0.0418091 3.6024895

H 0.8331013 0.2539686 2.7015242

C -0.0656007 -1.4435902 3.6605945

H -0.7096729 -1.7381770 2.8278253

H 0.8409309 -2.0516435 3.6078715

H -0.5887282 -1.7002045 4.5864320

H 0.8930131 0.3143508 4.4465647

H -1.6564401 0.6532051 2.7769236

H -0.7516935 1.9705728 3.5241594

**17 (TPSS)**

C -2.4145829 7.0642840 -0.2504966

C -2.4530977 5.6439310 -0.2256318

C -1.2223392 4.9367242 -0.1521745

C 0.0051579 5.6455466 -0.1056570

C 0.0064359 7.0558788 -0.1321203

C -1.2328199 7.7393022 -0.2055109

C 1.2313787 4.9367003 -0.0326416

C 2.4634174 5.6439573 0.0142562

C 2.4274908 7.0643013 -0.0141219

C 1.2469491 7.7393159 -0.0844082

C -1.2250196 3.5264963 -0.1258101

C 0.0025253 2.8166639 -0.0527674

C 1.2314804 3.5265307 -0.0062618

C 2.4515956 2.8211523 0.0663837

C 3.6860306 3.5370925 0.1132960

C 3.6617124 4.9280612 0.0861554

C -3.6526957 4.9280558 -0.2707400

C -3.6795473 3.5370934 -0.2457796

C -2.4464427 2.8211739 -0.1720357

C -2.4498365 1.4106976 -0.1457027

C -1.2259887 0.7035814 -0.0728565

C 0.0013792 1.4137069 -0.0264984

C 1.2271683 0.7035278 0.0464582

C 2.4524715 1.4106924 0.0928548

C -1.2271728 -0.7036200 -0.0464880

C -0.0012606 -1.4136531 0.0264774

C 1.2258367 -0.7035899 0.0728311

C 2.4498735 -1.4107692 0.1457164

C 3.6736847 -0.6996933 0.1920056

C 3.6749612 0.6996338 0.1658077

C -3.6736920 0.6996292 -0.1919993

C -3.6749836 -0.6996413 -0.1658134

C -2.4524202 -1.4107359 -0.0928764

C 4.9116874 1.4276636 0.2126344

C 4.8957776 2.7989336 0.1862468

C -4.8906596 2.7989485 -0.2910803

C -4.9090654 1.4276964 -0.2661132

C -2.4516102 -2.8211800 -0.0664046

C -1.2314557 -3.5265000 0.0062456

C -0.0026153 -2.8167790 0.0527610

C 1.2250240 -3.5265342 0.1258389

C 2.4464325 -2.8211842 0.1720618

C -1.2313555 -4.9367802 0.0326490

C -0.0051586 -5.6455808 0.1056720

C 1.2223599 -4.9367626 0.1522028

C -0.0064357 -7.0559236 0.1321307

C 1.2328105 -7.7393529 0.2055147

C 2.4146113 -7.0642730 0.2504995

C 2.4531249 -5.6439350 0.2256563

C -2.4634369 -5.6439279 -0.0142611

C -2.4275073 -7.0642964 0.0141225

C -1.2469529 -7.7393530 0.0844100

C 3.6526818 -4.9280304 0.2707483

C 3.6795768 -3.5371182 0.2458092

C -3.6860301 -3.5371134 -0.1133101

C -3.6616902 -4.9280324 -0.0861588

C 4.8906285 -2.7989372 0.2910856

C 4.9090855 -1.4276937 0.2661231

C -4.9116949 -1.4276895 -0.2126351

C -4.8957579 -2.7989156 -0.1862484

C 6.1331076 -0.6735473 0.3118940

C 6.1343742 0.6735801 0.2866366

C -6.1331436 0.6736397 -0.3118964

C -6.1343532 -0.6735793 -0.2866332

H 7.0701237 1.2215798 0.3223169

H 7.0679168 -1.2213858 0.3681048

H 5.8282842 -3.3432307 0.3472588

H 4.5896895 -5.4732687 0.3268734

H 3.3518905 -7.6070488 0.3065010

H 1.2261674 -8.8240195 0.2255898

H -1.2423000 -8.8240309 0.1050292

H -3.3658067 -7.6070639 -0.0214469

H -4.5997092 -5.4732888 -0.1218510

H -5.8344072 -3.3432558 -0.2220383

H -7.0701429 -1.2214123 -0.3223188

H -7.0678864 1.2215548 -0.3681105

H -5.8282879 3.3433562 -0.3472726

H -4.5897087 5.4733075 -0.3268962

H -3.3519273 7.6070100 -0.3065168

H -1.2261066 8.8240017 -0.2255927

H 1.2422388 8.8240145 -0.1050374

H 3.3658424 7.6070379 0.0214517

H 4.5997176 5.4733289 0.1218596

H 5.8343926 3.3433628 0.2220440

C -1.0041688 0.9169975 3.6115024

H -1.5734196 0.7758722 4.5379924

C 0.2567243 0.0444494 3.6027990

H 0.8381020 0.2561201 2.6984375

C -0.0652979 -1.4532433 3.6610357

H -0.7116849 -1.7444988 2.8260923

H 0.8430629 -2.0632495 3.6071579

H -0.5895527 -1.7064727 4.5903807

H 0.8990690 0.3164097 4.4506825

H -1.6621626 0.6569202 2.7753912

H -0.7583059 1.9810118 3.5230471

**17 (CAM-B3LYP)**

C -2.4145829 7.0642840 -0.2504966

C -2.4530977 5.6439310 -0.2256318

C -1.2223392 4.9367242 -0.1521745

C 0.0051579 5.6455466 -0.1056570

C 0.0064359 7.0558788 -0.1321203

C -1.2328199 7.7393022 -0.2055109

C 1.2313787 4.9367003 -0.0326416

C 2.4634174 5.6439573 0.0142562

C 2.4274908 7.0643013 -0.0141219

C 1.2469491 7.7393159 -0.0844082

C -1.2250196 3.5264963 -0.1258101

C 0.0025253 2.8166639 -0.0527674

C 1.2314804 3.5265307 -0.0062618

C 2.4515956 2.8211523 0.0663837

C 3.6860306 3.5370925 0.1132960

C 3.6617124 4.9280612 0.0861554

C -3.6526957 4.9280558 -0.2707400

C -3.6795473 3.5370934 -0.2457796

C -2.4464427 2.8211739 -0.1720357

C -2.4498365 1.4106976 -0.1457027

C -1.2259887 0.7035814 -0.0728565

C 0.0013792 1.4137069 -0.0264984

C 1.2271683 0.7035278 0.0464582

C 2.4524715 1.4106924 0.0928548

C -1.2271728 -0.7036200 -0.0464880

C -0.0012606 -1.4136531 0.0264774

C 1.2258367 -0.7035899 0.0728311

C 2.4498735 -1.4107692 0.1457164

C 3.6736847 -0.6996933 0.1920056

C 3.6749612 0.6996338 0.1658077

C -3.6736920 0.6996292 -0.1919993

C -3.6749836 -0.6996413 -0.1658134

C -2.4524202 -1.4107359 -0.0928764

C 4.9116874 1.4276636 0.2126344

C 4.8957776 2.7989336 0.1862468

C -4.8906596 2.7989485 -0.2910803

C -4.9090654 1.4276964 -0.2661132

C -2.4516102 -2.8211800 -0.0664046

C -1.2314557 -3.5265000 0.0062456

C -0.0026153 -2.8167790 0.0527610

C 1.2250240 -3.5265342 0.1258389

C 2.4464325 -2.8211842 0.1720618

C -1.2313555 -4.9367802 0.0326490

C -0.0051586 -5.6455808 0.1056720

C 1.2223599 -4.9367626 0.1522028

C -0.0064357 -7.0559236 0.1321307

C 1.2328105 -7.7393529 0.2055147

C 2.4146113 -7.0642730 0.2504995

C 2.4531249 -5.6439350 0.2256563

C -2.4634369 -5.6439279 -0.0142611

C -2.4275073 -7.0642964 0.0141225

C -1.2469529 -7.7393530 0.0844100

C 3.6526818 -4.9280304 0.2707483

C 3.6795768 -3.5371182 0.2458092

C -3.6860301 -3.5371134 -0.1133101

C -3.6616902 -4.9280324 -0.0861588

C 4.8906285 -2.7989372 0.2910856

C 4.9090855 -1.4276937 0.2661231

C -4.9116949 -1.4276895 -0.2126351

C -4.8957579 -2.7989156 -0.1862484

C 6.1331076 -0.6735473 0.3118940

C 6.1343742 0.6735801 0.2866366

C -6.1331436 0.6736397 -0.3118964

C -6.1343532 -0.6735793 -0.2866332

H 7.0701237 1.2215798 0.3223169

H 7.0679168 -1.2213858 0.3681048

H 5.8282842 -3.3432307 0.3472588

H 4.5896895 -5.4732687 0.3268734

H 3.3518905 -7.6070488 0.3065010

H 1.2261674 -8.8240195 0.2255898

H -1.2423000 -8.8240309 0.1050292

H -3.3658067 -7.6070639 -0.0214469

H -4.5997092 -5.4732888 -0.1218510

H -5.8344072 -3.3432558 -0.2220383

H -7.0701429 -1.2214123 -0.3223188

H -7.0678864 1.2215548 -0.3681105

H -5.8282879 3.3433562 -0.3472726

H -4.5897087 5.4733075 -0.3268962

H -3.3519273 7.6070100 -0.3065168

H -1.2261066 8.8240017 -0.2255927

H 1.2422388 8.8240145 -0.1050374

H 3.3658424 7.6070379 0.0214517

H 4.5997176 5.4733289 0.1218596

H 5.8343926 3.3433628 0.2220440

C -0.9986502 0.9098041 3.6107063

H -1.5669232 0.7715937 4.5327478

C 0.2512766 0.0422361 3.6019284

H 0.8318490 0.2534999 2.7027923

C -0.0661057 -1.4445498 3.6601610

H -0.7087250 -1.7379745 2.8285704

H 0.8391591 -2.0509436 3.6081079

H -0.5878282 -1.6994580 4.5849438

H 0.8915976 0.3136167 4.4451700

H -1.6554132 0.6539314 2.7777342

H -0.7525703 1.9690210 3.5244035

**18**

C -2.4145829 7.0642840 -0.2504966

C -2.4530977 5.6439310 -0.2256318

C -1.2223392 4.9367242 -0.1521745

C 0.0051579 5.6455466 -0.1056570

C 0.0064359 7.0558788 -0.1321203

C -1.2328199 7.7393022 -0.2055109

C 1.2313787 4.9367003 -0.0326416

C 2.4634174 5.6439573 0.0142562

C 2.4274908 7.0643013 -0.0141219

C 1.2469491 7.7393159 -0.0844082

C -1.2250196 3.5264963 -0.1258101

C 0.0025253 2.8166639 -0.0527674

C 1.2314804 3.5265307 -0.0062618

C 2.4515956 2.8211523 0.0663837

C 3.6860306 3.5370925 0.1132960

C 3.6617124 4.9280612 0.0861554

C -3.6526957 4.9280558 -0.2707400

C -3.6795473 3.5370934 -0.2457796

C -2.4464427 2.8211739 -0.1720357

C -2.4498365 1.4106976 -0.1457027

C -1.2259887 0.7035814 -0.0728565

C 0.0013792 1.4137069 -0.0264984

C 1.2271683 0.7035278 0.0464582

C 2.4524715 1.4106924 0.0928548

C -1.2271728 -0.7036200 -0.0464880

C -0.0012606 -1.4136531 0.0264774

C 1.2258367 -0.7035899 0.0728311

C 2.4498735 -1.4107692 0.1457164

C 3.6736847 -0.6996933 0.1920056

C 3.6749612 0.6996338 0.1658077

C -3.6736920 0.6996292 -0.1919993

C -3.6749836 -0.6996413 -0.1658134

C -2.4524202 -1.4107359 -0.0928764

C 4.9116874 1.4276636 0.2126344

C 4.8957776 2.7989336 0.1862468

C -4.8906596 2.7989485 -0.2910803

C -4.9090654 1.4276964 -0.2661132

C -2.4516102 -2.8211800 -0.0664046

C -1.2314557 -3.5265000 0.0062456

C -0.0026153 -2.8167790 0.0527610

C 1.2250240 -3.5265342 0.1258389

C 2.4464325 -2.8211842 0.1720618

C -1.2313555 -4.9367802 0.0326490

C -0.0051586 -5.6455808 0.1056720

C 1.2223599 -4.9367626 0.1522028

C -0.0064357 -7.0559236 0.1321307

C 1.2328105 -7.7393529 0.2055147

C 2.4146113 -7.0642730 0.2504995

C 2.4531249 -5.6439350 0.2256563

C -2.4634369 -5.6439279 -0.0142611

C -2.4275073 -7.0642964 0.0141225

C -1.2469529 -7.7393530 0.0844100

C 3.6526818 -4.9280304 0.2707483

C 3.6795768 -3.5371182 0.2458092

C -3.6860301 -3.5371134 -0.1133101

C -3.6616902 -4.9280324 -0.0861588

C 4.8906285 -2.7989372 0.2910856

C 4.9090855 -1.4276937 0.2661231

C -4.9116949 -1.4276895 -0.2126351

C -4.8957579 -2.7989156 -0.1862484

C 6.1331076 -0.6735473 0.3118940

C 6.1343742 0.6735801 0.2866366

C -6.1331436 0.6736397 -0.3118964

C -6.1343532 -0.6735793 -0.2866332

H 7.0701237 1.2215798 0.3223169

H 7.0679168 -1.2213858 0.3681048

H 5.8282842 -3.3432307 0.3472588

H 4.5896895 -5.4732687 0.3268734

H 3.3518905 -7.6070488 0.3065010

H 1.2261674 -8.8240195 0.2255898

H -1.2423000 -8.8240309 0.1050292

H -3.3658067 -7.6070639 -0.0214469

H -4.5997092 -5.4732888 -0.1218510

H -5.8344072 -3.3432558 -0.2220383

H -7.0701429 -1.2214123 -0.3223188

H -7.0678864 1.2215548 -0.3681105

H -5.8282879 3.3433562 -0.3472726

H -4.5897087 5.4733075 -0.3268962

H -3.3519273 7.6070100 -0.3065168

H -1.2261066 8.8240017 -0.2255927

H 1.2422388 8.8240145 -0.1050374

H 3.3658424 7.6070379 0.0214517

H 4.5997176 5.4733289 0.1218596

H 5.8343926 3.3433628 0.2220440

C -2.0267592 0.4394476 3.4079412

H -2.6349297 0.1380718 4.2692360

C -0.6900840 -0.2836552 3.4589496

H -0.0899154 0.0177442 2.5931686

C -0.8333460 -1.7955561 3.4796898

H -1.4059670 -2.1455937 2.6166219

H 0.1382807 -2.2946580 3.4549496

H -1.3592745 -2.1295349 4.3787107

H -0.1305590 0.0493204 4.3413999

H -2.5773910 0.1071998 2.5203117

C -1.8825577 1.9511577 3.3825794

H -2.8515149 2.4490484 3.2987116

H -1.3955847 2.3164445 4.2912855

H -1.2729483 2.2708498 2.5334577

**19**

C -2.4145829 7.0642840 -0.2504966

C -2.4530977 5.6439310 -0.2256318

C -1.2223392 4.9367242 -0.1521745

C 0.0051579 5.6455466 -0.1056570

C 0.0064359 7.0558788 -0.1321203

C -1.2328199 7.7393022 -0.2055109

C 1.2313787 4.9367003 -0.0326416

C 2.4634174 5.6439573 0.0142562

C 2.4274908 7.0643013 -0.0141219

C 1.2469491 7.7393159 -0.0844082

C -1.2250196 3.5264963 -0.1258101

C 0.0025253 2.8166639 -0.0527674

C 1.2314804 3.5265307 -0.0062618

C 2.4515956 2.8211523 0.0663837

C 3.6860306 3.5370925 0.1132960

C 3.6617124 4.9280612 0.0861554

C -3.6526957 4.9280558 -0.2707400

C -3.6795473 3.5370934 -0.2457796

C -2.4464427 2.8211739 -0.1720357

C -2.4498365 1.4106976 -0.1457027

C -1.2259887 0.7035814 -0.0728565

C 0.0013792 1.4137069 -0.0264984

C 1.2271683 0.7035278 0.0464582

C 2.4524715 1.4106924 0.0928548

C -1.2271728 -0.7036200 -0.0464880

C -0.0012606 -1.4136531 0.0264774

C 1.2258367 -0.7035899 0.0728311

C 2.4498735 -1.4107692 0.1457164

C 3.6736847 -0.6996933 0.1920056

C 3.6749612 0.6996338 0.1658077

C -3.6736920 0.6996292 -0.1919993

C -3.6749836 -0.6996413 -0.1658134

C -2.4524202 -1.4107359 -0.0928764

C 4.9116874 1.4276636 0.2126344

C 4.8957776 2.7989336 0.1862468

C -4.8906596 2.7989485 -0.2910803

C -4.9090654 1.4276964 -0.2661132

C -2.4516102 -2.8211800 -0.0664046

C -1.2314557 -3.5265000 0.0062456

C -0.0026153 -2.8167790 0.0527610

C 1.2250240 -3.5265342 0.1258389

C 2.4464325 -2.8211842 0.1720618

C -1.2313555 -4.9367802 0.0326490

C -0.0051586 -5.6455808 0.1056720

C 1.2223599 -4.9367626 0.1522028

C -0.0064357 -7.0559236 0.1321307

C 1.2328105 -7.7393529 0.2055147

C 2.4146113 -7.0642730 0.2504995

C 2.4531249 -5.6439350 0.2256563

C -2.4634369 -5.6439279 -0.0142611

C -2.4275073 -7.0642964 0.0141225

C -1.2469529 -7.7393530 0.0844100

C 3.6526818 -4.9280304 0.2707483

C 3.6795768 -3.5371182 0.2458092

C -3.6860301 -3.5371134 -0.1133101

C -3.6616902 -4.9280324 -0.0861588

C 4.8906285 -2.7989372 0.2910856

C 4.9090855 -1.4276937 0.2661231

C -4.9116949 -1.4276895 -0.2126351

C -4.8957579 -2.7989156 -0.1862484

C 6.1331076 -0.6735473 0.3118940

C 6.1343742 0.6735801 0.2866366

C -6.1331436 0.6736397 -0.3118964

C -6.1343532 -0.6735793 -0.2866332

H 7.0701237 1.2215798 0.3223169

H 7.0679168 -1.2213858 0.3681048

H 5.8282842 -3.3432307 0.3472588

H 4.5896895 -5.4732687 0.3268734

H 3.3518905 -7.6070488 0.3065010

H 1.2261674 -8.8240195 0.2255898

H -1.2423000 -8.8240309 0.1050292

H -3.3658067 -7.6070639 -0.0214469

H -4.5997092 -5.4732888 -0.1218510

H -5.8344072 -3.3432558 -0.2220383

H -7.0701429 -1.2214123 -0.3223188

H -7.0678864 1.2215548 -0.3681105

H -5.8282879 3.3433562 -0.3472726

H -4.5897087 5.4733075 -0.3268962

H -3.3519273 7.6070100 -0.3065168

H -1.2261066 8.8240017 -0.2255927

H 1.2422388 8.8240145 -0.1050374

H 3.3658424 7.6070379 0.0214517

H 4.5997176 5.4733289 0.1218596

H 5.8343926 3.3433628 0.2220440

C -1.1535772 0.9446041 3.5627232

H -1.8444564 0.7266115 4.3840225

C 0.0510657 0.0238570 3.6643579

H 0.7488322 0.2563639 2.8512722

C -0.3010775 -1.4547140 3.6134998

H -0.8563826 -1.6612597 2.6911970

H -0.9827086 -1.6992580 4.4378031

H 0.5948408 0.2378096 4.5933028

H -1.6989522 0.7147752 2.6410252

C -0.7755075 2.4167468 3.5810248

H -1.6473800 3.0617028 3.4595980

H -0.2891705 2.6875539 4.5213599

H -0.0781820 2.6536544 2.7724627

C 0.9221122 -2.3524870 3.6744446

H 0.6553524 -3.4098890 3.5931881

H 1.6135887 -2.1239165 2.8579271

H 1.4667791 -2.2108998 4.6128768

**20**

C -2.4145829 7.0642840 -0.2504966

C -2.4530977 5.6439310 -0.2256318

C -1.2223392 4.9367242 -0.1521745

C 0.0051579 5.6455466 -0.1056570

C 0.0064359 7.0558788 -0.1321203

C -1.2328199 7.7393022 -0.2055109

C 1.2313787 4.9367003 -0.0326416

C 2.4634174 5.6439573 0.0142562

C 2.4274908 7.0643013 -0.0141219

C 1.2469491 7.7393159 -0.0844082

C -1.2250196 3.5264963 -0.1258101

C 0.0025253 2.8166639 -0.0527674

C 1.2314804 3.5265307 -0.0062618

C 2.4515956 2.8211523 0.0663837

C 3.6860306 3.5370925 0.1132960

C 3.6617124 4.9280612 0.0861554

C -3.6526957 4.9280558 -0.2707400

C -3.6795473 3.5370934 -0.2457796

C -2.4464427 2.8211739 -0.1720357

C -2.4498365 1.4106976 -0.1457027

C -1.2259887 0.7035814 -0.0728565

C 0.0013792 1.4137069 -0.0264984

C 1.2271683 0.7035278 0.0464582

C 2.4524715 1.4106924 0.0928548

C -1.2271728 -0.7036200 -0.0464880

C -0.0012606 -1.4136531 0.0264774

C 1.2258367 -0.7035899 0.0728311

C 2.4498735 -1.4107692 0.1457164

C 3.6736847 -0.6996933 0.1920056

C 3.6749612 0.6996338 0.1658077

C -3.6736920 0.6996292 -0.1919993

C -3.6749836 -0.6996413 -0.1658134

C -2.4524202 -1.4107359 -0.0928764

C 4.9116874 1.4276636 0.2126344

C 4.8957776 2.7989336 0.1862468

C -4.8906596 2.7989485 -0.2910803

C -4.9090654 1.4276964 -0.2661132

C -2.4516102 -2.8211800 -0.0664046

C -1.2314557 -3.5265000 0.0062456

C -0.0026153 -2.8167790 0.0527610

C 1.2250240 -3.5265342 0.1258389

C 2.4464325 -2.8211842 0.1720618

C -1.2313555 -4.9367802 0.0326490

C -0.0051586 -5.6455808 0.1056720

C 1.2223599 -4.9367626 0.1522028

C -0.0064357 -7.0559236 0.1321307

C 1.2328105 -7.7393529 0.2055147

C 2.4146113 -7.0642730 0.2504995

C 2.4531249 -5.6439350 0.2256563

C -2.4634369 -5.6439279 -0.0142611

C -2.4275073 -7.0642964 0.0141225

C -1.2469529 -7.7393530 0.0844100

C 3.6526818 -4.9280304 0.2707483

C 3.6795768 -3.5371182 0.2458092

C -3.6860301 -3.5371134 -0.1133101

C -3.6616902 -4.9280324 -0.0861588

C 4.8906285 -2.7989372 0.2910856

C 4.9090855 -1.4276937 0.2661231

C -4.9116949 -1.4276895 -0.2126351

C -4.8957579 -2.7989156 -0.1862484

C 6.1331076 -0.6735473 0.3118940

C 6.1343742 0.6735801 0.2866366

C -6.1331436 0.6736397 -0.3118964

C -6.1343532 -0.6735793 -0.2866332

H 7.0701237 1.2215798 0.3223169

H 7.0679168 -1.2213858 0.3681048

H 5.8282842 -3.3432307 0.3472588

H 4.5896895 -5.4732687 0.3268734

H 3.3518905 -7.6070488 0.3065010

H 1.2261674 -8.8240195 0.2255898

H -1.2423000 -8.8240309 0.1050292

H -3.3658067 -7.6070639 -0.0214469

H -4.5997092 -5.4732888 -0.1218510

H -5.8344072 -3.3432558 -0.2220383

H -7.0701429 -1.2214123 -0.3223188

H -7.0678864 1.2215548 -0.3681105

H -5.8282879 3.3433562 -0.3472726

H -4.5897087 5.4733075 -0.3268962

H -3.3519273 7.6070100 -0.3065168

H -1.2261066 8.8240017 -0.2255927

H 1.2422388 8.8240145 -0.1050374

H 3.3658424 7.6070379 0.0214517

H 4.5997176 5.4733289 0.1218596

H 5.8343926 3.3433628 0.2220440

C -1.4764058 1.0045647 3.5761634

H -2.1281461 0.8514508 4.4455855

C -0.3630836 -0.0296092 3.5897713

H 0.2981789 0.1402662 2.7317439

C -0.8593942 -1.4660963 3.5560387

H -1.4551657 -1.6175293 2.6495478

H -1.5422177 -1.6343824 4.3968737

H 0.2595652 0.1187189 4.4812535

H -2.1087985 0.8453732 2.6946268

C -0.9668929 2.4374969 3.5741906

H -0.3408976 2.5984239 4.4597263

H -0.3084753 2.5813905 2.7107979

C 0.2697481 -2.4815671 3.6009269

H -0.1019995 -3.5049651 3.5079919

H 0.9796541 -2.3133985 2.7870404

H 0.8255936 -2.4107017 4.5401978

C -2.0815595 3.4691684 3.5399223

H -2.7301460 3.3777725 4.4160839

H -1.6868151 4.4879872 3.5206802

H -2.7069946 3.3412203 2.6525702

**21**

C -2.4145829 7.0642840 -0.2504966

C -2.4530977 5.6439310 -0.2256318

C -1.2223392 4.9367242 -0.1521745

C 0.0051579 5.6455466 -0.1056570

C 0.0064359 7.0558788 -0.1321203

C -1.2328199 7.7393022 -0.2055109

C 1.2313787 4.9367003 -0.0326416

C 2.4634174 5.6439573 0.0142562

C 2.4274908 7.0643013 -0.0141219

C 1.2469491 7.7393159 -0.0844082

C -1.2250196 3.5264963 -0.1258101

C 0.0025253 2.8166639 -0.0527674

C 1.2314804 3.5265307 -0.0062618

C 2.4515956 2.8211523 0.0663837

C 3.6860306 3.5370925 0.1132960

C 3.6617124 4.9280612 0.0861554

C -3.6526957 4.9280558 -0.2707400

C -3.6795473 3.5370934 -0.2457796

C -2.4464427 2.8211739 -0.1720357

C -2.4498365 1.4106976 -0.1457027

C -1.2259887 0.7035814 -0.0728565

C 0.0013792 1.4137069 -0.0264984

C 1.2271683 0.7035278 0.0464582

C 2.4524715 1.4106924 0.0928548

C -1.2271728 -0.7036200 -0.0464880

C -0.0012606 -1.4136531 0.0264774

C 1.2258367 -0.7035899 0.0728311

C 2.4498735 -1.4107692 0.1457164

C 3.6736847 -0.6996933 0.1920056

C 3.6749612 0.6996338 0.1658077

C -3.6736920 0.6996292 -0.1919993

C -3.6749836 -0.6996413 -0.1658134

C -2.4524202 -1.4107359 -0.0928764

C 4.9116874 1.4276636 0.2126344

C 4.8957776 2.7989336 0.1862468

C -4.8906596 2.7989485 -0.2910803

C -4.9090654 1.4276964 -0.2661132

C -2.4516102 -2.8211800 -0.0664046

C -1.2314557 -3.5265000 0.0062456

C -0.0026153 -2.8167790 0.0527610

C 1.2250240 -3.5265342 0.1258389

C 2.4464325 -2.8211842 0.1720618

C -1.2313555 -4.9367802 0.0326490

C -0.0051586 -5.6455808 0.1056720

C 1.2223599 -4.9367626 0.1522028

C -0.0064357 -7.0559236 0.1321307

C 1.2328105 -7.7393529 0.2055147

C 2.4146113 -7.0642730 0.2504995

C 2.4531249 -5.6439350 0.2256563

C -2.4634369 -5.6439279 -0.0142611

C -2.4275073 -7.0642964 0.0141225

C -1.2469529 -7.7393530 0.0844100

C 3.6526818 -4.9280304 0.2707483

C 3.6795768 -3.5371182 0.2458092

C -3.6860301 -3.5371134 -0.1133101

C -3.6616902 -4.9280324 -0.0861588

C 4.8906285 -2.7989372 0.2910856

C 4.9090855 -1.4276937 0.2661231

C -4.9116949 -1.4276895 -0.2126351

C -4.8957579 -2.7989156 -0.1862484

C 6.1331076 -0.6735473 0.3118940

C 6.1343742 0.6735801 0.2866366

C -6.1331436 0.6736397 -0.3118964

C -6.1343532 -0.6735793 -0.2866332

H 7.0701237 1.2215798 0.3223169

H 7.0679168 -1.2213858 0.3681048

H 5.8282842 -3.3432307 0.3472588

H 4.5896895 -5.4732687 0.3268734

H 3.3518905 -7.6070488 0.3065010

H 1.2261674 -8.8240195 0.2255898

H -1.2423000 -8.8240309 0.1050292

H -3.3658067 -7.6070639 -0.0214469

H -4.5997092 -5.4732888 -0.1218510

H -5.8344072 -3.3432558 -0.2220383

H -7.0701429 -1.2214123 -0.3223188

H -7.0678864 1.2215548 -0.3681105

H -5.8282879 3.3433562 -0.3472726

H -4.5897087 5.4733075 -0.3268962

H -3.3519273 7.6070100 -0.3065168

H -1.2261066 8.8240017 -0.2255927

H 1.2422388 8.8240145 -0.1050374

H 3.3658424 7.6070379 0.0214517

H 4.5997176 5.4733289 0.1218596

H 5.8343926 3.3433628 0.2220440

C -0.7665166 -0.6859931 3.6860656

C 0.7300755 -0.6871334 3.7420416

C -0.0159987 0.6100612 3.6815045

H -1.2412754 -0.9995322 2.7648032

H -1.3098746 -0.9573304 4.5827921

H 1.2726665 -1.0035406 2.8601431

H 1.2036474 -0.9577192 4.6777366

H -0.0478413 1.2208658 4.5752009

H 0.0175113 1.1729555 2.7573520

**22**

C -2.4145829 7.0642840 -0.2504966

C -2.4530977 5.6439310 -0.2256318

C -1.2223392 4.9367242 -0.1521745

C 0.0051579 5.6455466 -0.1056570

C 0.0064359 7.0558788 -0.1321203

C -1.2328199 7.7393022 -0.2055109

C 1.2313787 4.9367003 -0.0326416

C 2.4634174 5.6439573 0.0142562

C 2.4274908 7.0643013 -0.0141219

C 1.2469491 7.7393159 -0.0844082

C -1.2250196 3.5264963 -0.1258101

C 0.0025253 2.8166639 -0.0527674

C 1.2314804 3.5265307 -0.0062618

C 2.4515956 2.8211523 0.0663837

C 3.6860306 3.5370925 0.1132960

C 3.6617124 4.9280612 0.0861554

C -3.6526957 4.9280558 -0.2707400

C -3.6795473 3.5370934 -0.2457796

C -2.4464427 2.8211739 -0.1720357

C -2.4498365 1.4106976 -0.1457027

C -1.2259887 0.7035814 -0.0728565

C 0.0013792 1.4137069 -0.0264984

C 1.2271683 0.7035278 0.0464582

C 2.4524715 1.4106924 0.0928548

C -1.2271728 -0.7036200 -0.0464880

C -0.0012606 -1.4136531 0.0264774

C 1.2258367 -0.7035899 0.0728311

C 2.4498735 -1.4107692 0.1457164

C 3.6736847 -0.6996933 0.1920056

C 3.6749612 0.6996338 0.1658077

C -3.6736920 0.6996292 -0.1919993

C -3.6749836 -0.6996413 -0.1658134

C -2.4524202 -1.4107359 -0.0928764

C 4.9116874 1.4276636 0.2126344

C 4.8957776 2.7989336 0.1862468

C -4.8906596 2.7989485 -0.2910803

C -4.9090654 1.4276964 -0.2661132

C -2.4516102 -2.8211800 -0.0664046

C -1.2314557 -3.5265000 0.0062456

C -0.0026153 -2.8167790 0.0527610

C 1.2250240 -3.5265342 0.1258389

C 2.4464325 -2.8211842 0.1720618

C -1.2313555 -4.9367802 0.0326490

C -0.0051586 -5.6455808 0.1056720

C 1.2223599 -4.9367626 0.1522028

C -0.0064357 -7.0559236 0.1321307

C 1.2328105 -7.7393529 0.2055147

C 2.4146113 -7.0642730 0.2504995

C 2.4531249 -5.6439350 0.2256563

C -2.4634369 -5.6439279 -0.0142611

C -2.4275073 -7.0642964 0.0141225

C -1.2469529 -7.7393530 0.0844100

C 3.6526818 -4.9280304 0.2707483

C 3.6795768 -3.5371182 0.2458092

C -3.6860301 -3.5371134 -0.1133101

C -3.6616902 -4.9280324 -0.0861588

C 4.8906285 -2.7989372 0.2910856

C 4.9090855 -1.4276937 0.2661231

C -4.9116949 -1.4276895 -0.2126351

C -4.8957579 -2.7989156 -0.1862484

C 6.1331076 -0.6735473 0.3118940

C 6.1343742 0.6735801 0.2866366

C -6.1331436 0.6736397 -0.3118964

C -6.1343532 -0.6735793 -0.2866332

H 7.0701237 1.2215798 0.3223169

H 7.0679168 -1.2213858 0.3681048

H 5.8282842 -3.3432307 0.3472588

H 4.5896895 -5.4732687 0.3268734

H 3.3518905 -7.6070488 0.3065010

H 1.2261674 -8.8240195 0.2255898

H -1.2423000 -8.8240309 0.1050292

H -3.3658067 -7.6070639 -0.0214469

H -4.5997092 -5.4732888 -0.1218510

H -5.8344072 -3.3432558 -0.2220383

H -7.0701429 -1.2214123 -0.3223188

H -7.0678864 1.2215548 -0.3681105

H -5.8282879 3.3433562 -0.3472726

H -4.5897087 5.4733075 -0.3268962

H -3.3519273 7.6070100 -0.3065168

H -1.2261066 8.8240017 -0.2255927

H 1.2422388 8.8240145 -0.1050374

H 3.3658424 7.6070379 0.0214517

H 4.5997176 5.4733289 0.1218596

H 5.8343926 3.3433628 0.2220440

C -0.8590543 -0.5296056 3.6486458

C 0.6850244 -0.5262001 3.7186116

C 0.6837109 1.0188710 3.6729534

C -0.8606200 1.0157358 3.6104574

H -1.2709006 -1.0045286 2.7572034

H -1.3536542 -0.9646730 4.5187073

H 1.1791704 -1.0047564 2.8722084

H 1.0992769 -0.9535298 4.6333168

H 1.1718527 1.4461792 2.7959225

H 1.1029521 1.5012454 4.5575234

H -1.2795166 1.4470072 2.7001004

H -1.3505506 1.4907581 4.4621118

**22 (TPSS)**

C -2.4145829 7.0642840 -0.2504966

C -2.4530977 5.6439310 -0.2256318

C -1.2223392 4.9367242 -0.1521745

C 0.0051579 5.6455466 -0.1056570

C 0.0064359 7.0558788 -0.1321203

C -1.2328199 7.7393022 -0.2055109

C 1.2313787 4.9367003 -0.0326416

C 2.4634174 5.6439573 0.0142562

C 2.4274908 7.0643013 -0.0141219

C 1.2469491 7.7393159 -0.0844082

C -1.2250196 3.5264963 -0.1258101

C 0.0025253 2.8166639 -0.0527674

C 1.2314804 3.5265307 -0.0062618

C 2.4515956 2.8211523 0.0663837

C 3.6860306 3.5370925 0.1132960

C 3.6617124 4.9280612 0.0861554

C -3.6526957 4.9280558 -0.2707400

C -3.6795473 3.5370934 -0.2457796

C -2.4464427 2.8211739 -0.1720357

C -2.4498365 1.4106976 -0.1457027

C -1.2259887 0.7035814 -0.0728565

C 0.0013792 1.4137069 -0.0264984

C 1.2271683 0.7035278 0.0464582

C 2.4524715 1.4106924 0.0928548

C -1.2271728 -0.7036200 -0.0464880

C -0.0012606 -1.4136531 0.0264774

C 1.2258367 -0.7035899 0.0728311

C 2.4498735 -1.4107692 0.1457164

C 3.6736847 -0.6996933 0.1920056

C 3.6749612 0.6996338 0.1658077

C -3.6736920 0.6996292 -0.1919993

C -3.6749836 -0.6996413 -0.1658134

C -2.4524202 -1.4107359 -0.0928764

C 4.9116874 1.4276636 0.2126344

C 4.8957776 2.7989336 0.1862468

C -4.8906596 2.7989485 -0.2910803

C -4.9090654 1.4276964 -0.2661132

C -2.4516102 -2.8211800 -0.0664046

C -1.2314557 -3.5265000 0.0062456

C -0.0026153 -2.8167790 0.0527610

C 1.2250240 -3.5265342 0.1258389

C 2.4464325 -2.8211842 0.1720618

C -1.2313555 -4.9367802 0.0326490

C -0.0051586 -5.6455808 0.1056720

C 1.2223599 -4.9367626 0.1522028

C -0.0064357 -7.0559236 0.1321307

C 1.2328105 -7.7393529 0.2055147

C 2.4146113 -7.0642730 0.2504995

C 2.4531249 -5.6439350 0.2256563

C -2.4634369 -5.6439279 -0.0142611

C -2.4275073 -7.0642964 0.0141225

C -1.2469529 -7.7393530 0.0844100

C 3.6526818 -4.9280304 0.2707483

C 3.6795768 -3.5371182 0.2458092

C -3.6860301 -3.5371134 -0.1133101

C -3.6616902 -4.9280324 -0.0861588

C 4.8906285 -2.7989372 0.2910856

C 4.9090855 -1.4276937 0.2661231

C -4.9116949 -1.4276895 -0.2126351

C -4.8957579 -2.7989156 -0.1862484

C 6.1331076 -0.6735473 0.3118940

C 6.1343742 0.6735801 0.2866366

C -6.1331436 0.6736397 -0.3118964

C -6.1343532 -0.6735793 -0.2866332

H 7.0701237 1.2215798 0.3223169

H 7.0679168 -1.2213858 0.3681048

H 5.8282842 -3.3432307 0.3472588

H 4.5896895 -5.4732687 0.3268734

H 3.3518905 -7.6070488 0.3065010

H 1.2261674 -8.8240195 0.2255898

H -1.2423000 -8.8240309 0.1050292

H -3.3658067 -7.6070639 -0.0214469

H -4.5997092 -5.4732888 -0.1218510

H -5.8344072 -3.3432558 -0.2220383

H -7.0701429 -1.2214123 -0.3223188

H -7.0678864 1.2215548 -0.3681105

H -5.8282879 3.3433562 -0.3472726

H -4.5897087 5.4733075 -0.3268962

H -3.3519273 7.6070100 -0.3065168

H -1.2261066 8.8240017 -0.2255927

H 1.2422388 8.8240145 -0.1050374

H 3.3658424 7.6070379 0.0214517

H 4.5997176 5.4733289 0.1218596

H 5.8343926 3.3433628 0.2220440

C -0.8652328 -0.5357541 3.6483306

C 0.6914562 -0.5323299 3.7192831

C 0.6900141 1.0254196 3.6728465

C -0.8668760 1.0222111 3.6095790

H -1.2765701 -1.0112004 2.7543274

H -1.3603756 -0.9705410 4.5217060

H 1.1857526 -1.0114972 2.8704454

H 1.1053869 -0.9592654 4.6376350

H 1.1780617 1.4529406 2.7931632

H 1.1091373 1.5080869 4.5607319

H -1.2855256 1.4539459 2.6966464

H -1.3570984 1.4970917 4.4646246

**22 (CAM-B3LYP)**

C -2.4145829 7.0642840 -0.2504966

C -2.4530977 5.6439310 -0.2256318

C -1.2223392 4.9367242 -0.1521745

C 0.0051579 5.6455466 -0.1056570

C 0.0064359 7.0558788 -0.1321203

C -1.2328199 7.7393022 -0.2055109

C 1.2313787 4.9367003 -0.0326416

C 2.4634174 5.6439573 0.0142562

C 2.4274908 7.0643013 -0.0141219

C 1.2469491 7.7393159 -0.0844082

C -1.2250196 3.5264963 -0.1258101

C 0.0025253 2.8166639 -0.0527674

C 1.2314804 3.5265307 -0.0062618

C 2.4515956 2.8211523 0.0663837

C 3.6860306 3.5370925 0.1132960

C 3.6617124 4.9280612 0.0861554

C -3.6526957 4.9280558 -0.2707400

C -3.6795473 3.5370934 -0.2457796

C -2.4464427 2.8211739 -0.1720357

C -2.4498365 1.4106976 -0.1457027

C -1.2259887 0.7035814 -0.0728565

C 0.0013792 1.4137069 -0.0264984

C 1.2271683 0.7035278 0.0464582

C 2.4524715 1.4106924 0.0928548

C -1.2271728 -0.7036200 -0.0464880

C -0.0012606 -1.4136531 0.0264774

C 1.2258367 -0.7035899 0.0728311

C 2.4498735 -1.4107692 0.1457164

C 3.6736847 -0.6996933 0.1920056

C 3.6749612 0.6996338 0.1658077

C -3.6736920 0.6996292 -0.1919993

C -3.6749836 -0.6996413 -0.1658134

C -2.4524202 -1.4107359 -0.0928764

C 4.9116874 1.4276636 0.2126344

C 4.8957776 2.7989336 0.1862468

C -4.8906596 2.7989485 -0.2910803

C -4.9090654 1.4276964 -0.2661132

C -2.4516102 -2.8211800 -0.0664046

C -1.2314557 -3.5265000 0.0062456

C -0.0026153 -2.8167790 0.0527610

C 1.2250240 -3.5265342 0.1258389

C 2.4464325 -2.8211842 0.1720618

C -1.2313555 -4.9367802 0.0326490

C -0.0051586 -5.6455808 0.1056720

C 1.2223599 -4.9367626 0.1522028

C -0.0064357 -7.0559236 0.1321307

C 1.2328105 -7.7393529 0.2055147

C 2.4146113 -7.0642730 0.2504995

C 2.4531249 -5.6439350 0.2256563

C -2.4634369 -5.6439279 -0.0142611

C -2.4275073 -7.0642964 0.0141225

C -1.2469529 -7.7393530 0.0844100

C 3.6526818 -4.9280304 0.2707483

C 3.6795768 -3.5371182 0.2458092

C -3.6860301 -3.5371134 -0.1133101

C -3.6616902 -4.9280324 -0.0861588

C 4.8906285 -2.7989372 0.2910856

C 4.9090855 -1.4276937 0.2661231

C -4.9116949 -1.4276895 -0.2126351

C -4.8957579 -2.7989156 -0.1862484

C 6.1331076 -0.6735473 0.3118940

C 6.1343742 0.6735801 0.2866366

C -6.1331436 0.6736397 -0.3118964

C -6.1343532 -0.6735793 -0.2866332

H 7.0701237 1.2215798 0.3223169

H 7.0679168 -1.2213858 0.3681048

H 5.8282842 -3.3432307 0.3472588

H 4.5896895 -5.4732687 0.3268734

H 3.3518905 -7.6070488 0.3065010

H 1.2261674 -8.8240195 0.2255898

H -1.2423000 -8.8240309 0.1050292

H -3.3658067 -7.6070639 -0.0214469

H -4.5997092 -5.4732888 -0.1218510

H -5.8344072 -3.3432558 -0.2220383

H -7.0701429 -1.2214123 -0.3223188

H -7.0678864 1.2215548 -0.3681105

H -5.8282879 3.3433562 -0.3472726

H -4.5897087 5.4733075 -0.3268962

H -3.3519273 7.6070100 -0.3065168

H -1.2261066 8.8240017 -0.2255927

H 1.2422388 8.8240145 -0.1050374

H 3.3658424 7.6070379 0.0214517

H 4.5997176 5.4733289 0.1218596

H 5.8343926 3.3433628 0.2220440

C -0.8590701 -0.5240734 3.6048659

C 0.6858646 -0.5183156 3.6849071

C 0.6830536 1.0277538 3.6289090

C -0.8624525 1.0224996 3.5627502

H -1.2632424 -1.0000397 2.7136594

H -1.3566804 -0.9561025 4.4717545

H 1.1856589 -1.0011876 2.8476159

H 1.0915895 -0.9361770 4.6049895

H 1.1711067 1.4479704 2.7517403

H 1.0981366 1.5146003 4.5100725

H -1.2795674 1.4505368 2.6532400

H -1.3519422 1.4961704 4.4123326

**23**

C -2.4145829 7.0642840 -0.2504966

C -2.4530977 5.6439310 -0.2256318

C -1.2223392 4.9367242 -0.1521745

C 0.0051579 5.6455466 -0.1056570

C 0.0064359 7.0558788 -0.1321203

C -1.2328199 7.7393022 -0.2055109

C 1.2313787 4.9367003 -0.0326416

C 2.4634174 5.6439573 0.0142562

C 2.4274908 7.0643013 -0.0141219

C 1.2469491 7.7393159 -0.0844082

C -1.2250196 3.5264963 -0.1258101

C 0.0025253 2.8166639 -0.0527674

C 1.2314804 3.5265307 -0.0062618

C 2.4515956 2.8211523 0.0663837

C 3.6860306 3.5370925 0.1132960

C 3.6617124 4.9280612 0.0861554

C -3.6526957 4.9280558 -0.2707400

C -3.6795473 3.5370934 -0.2457796

C -2.4464427 2.8211739 -0.1720357

C -2.4498365 1.4106976 -0.1457027

C -1.2259887 0.7035814 -0.0728565

C 0.0013792 1.4137069 -0.0264984

C 1.2271683 0.7035278 0.0464582

C 2.4524715 1.4106924 0.0928548

C -1.2271728 -0.7036200 -0.0464880

C -0.0012606 -1.4136531 0.0264774

C 1.2258367 -0.7035899 0.0728311

C 2.4498735 -1.4107692 0.1457164

C 3.6736847 -0.6996933 0.1920056

C 3.6749612 0.6996338 0.1658077

C -3.6736920 0.6996292 -0.1919993

C -3.6749836 -0.6996413 -0.1658134

C -2.4524202 -1.4107359 -0.0928764

C 4.9116874 1.4276636 0.2126344

C 4.8957776 2.7989336 0.1862468

C -4.8906596 2.7989485 -0.2910803

C -4.9090654 1.4276964 -0.2661132

C -2.4516102 -2.8211800 -0.0664046

C -1.2314557 -3.5265000 0.0062456

C -0.0026153 -2.8167790 0.0527610

C 1.2250240 -3.5265342 0.1258389

C 2.4464325 -2.8211842 0.1720618

C -1.2313555 -4.9367802 0.0326490

C -0.0051586 -5.6455808 0.1056720

C 1.2223599 -4.9367626 0.1522028

C -0.0064357 -7.0559236 0.1321307

C 1.2328105 -7.7393529 0.2055147

C 2.4146113 -7.0642730 0.2504995

C 2.4531249 -5.6439350 0.2256563

C -2.4634369 -5.6439279 -0.0142611

C -2.4275073 -7.0642964 0.0141225

C -1.2469529 -7.7393530 0.0844100

C 3.6526818 -4.9280304 0.2707483

C 3.6795768 -3.5371182 0.2458092

C -3.6860301 -3.5371134 -0.1133101

C -3.6616902 -4.9280324 -0.0861588

C 4.8906285 -2.7989372 0.2910856

C 4.9090855 -1.4276937 0.2661231

C -4.9116949 -1.4276895 -0.2126351

C -4.8957579 -2.7989156 -0.1862484

C 6.1331076 -0.6735473 0.3118940

C 6.1343742 0.6735801 0.2866366

C -6.1331436 0.6736397 -0.3118964

C -6.1343532 -0.6735793 -0.2866332

H 7.0701237 1.2215798 0.3223169

H 7.0679168 -1.2213858 0.3681048

H 5.8282842 -3.3432307 0.3472588

H 4.5896895 -5.4732687 0.3268734

H 3.3518905 -7.6070488 0.3065010

H 1.2261674 -8.8240195 0.2255898

H -1.2423000 -8.8240309 0.1050292

H -3.3658067 -7.6070639 -0.0214469

H -4.5997092 -5.4732888 -0.1218510

H -5.8344072 -3.3432558 -0.2220383

H -7.0701429 -1.2214123 -0.3223188

H -7.0678864 1.2215548 -0.3681105

H -5.8282879 3.3433562 -0.3472726

H -4.5897087 5.4733075 -0.3268962

H -3.3519273 7.6070100 -0.3065168

H -1.2261066 8.8240017 -0.2255927

H 1.2422388 8.8240145 -0.1050374

H 3.3658424 7.6070379 0.0214517

H 4.5997176 5.4733289 0.1218596

H 5.8343926 3.3433628 0.2220440

C -0.8933781 -0.8133097 3.9227497

C 0.6251900 -0.7721767 3.7652853

C 0.9944435 0.6081539 4.2952659

C -0.1021088 1.5136714 3.7309085

C -1.3640392 0.6275940 3.6434221

H -1.3675196 -1.5456656 3.2648608

H -1.1442259 -1.0964135 4.9497488

H 0.8944962 -0.8426180 2.7074550

H 1.1327834 -1.5884077 4.2845968

H 0.9545950 0.6029104 5.3902962

H 1.9983553 0.9274799 4.0059474

H -0.2582754 2.4091113 4.3362733

H 0.1840978 1.8554660 2.7332164

H -2.1261875 0.9389028 4.3609147

H -1.8206060 0.7059368 2.6549770

**24**

C -2.4145829 7.0642840 -0.2504966

C -2.4530977 5.6439310 -0.2256318

C -1.2223392 4.9367242 -0.1521745

C 0.0051579 5.6455466 -0.1056570

C 0.0064359 7.0558788 -0.1321203

C -1.2328199 7.7393022 -0.2055109

C 1.2313787 4.9367003 -0.0326416

C 2.4634174 5.6439573 0.0142562

C 2.4274908 7.0643013 -0.0141219

C 1.2469491 7.7393159 -0.0844082

C -1.2250196 3.5264963 -0.1258101

C 0.0025253 2.8166639 -0.0527674

C 1.2314804 3.5265307 -0.0062618

C 2.4515956 2.8211523 0.0663837

C 3.6860306 3.5370925 0.1132960

C 3.6617124 4.9280612 0.0861554

C -3.6526957 4.9280558 -0.2707400

C -3.6795473 3.5370934 -0.2457796

C -2.4464427 2.8211739 -0.1720357

C -2.4498365 1.4106976 -0.1457027

C -1.2259887 0.7035814 -0.0728565

C 0.0013792 1.4137069 -0.0264984

C 1.2271683 0.7035278 0.0464582

C 2.4524715 1.4106924 0.0928548

C -1.2271728 -0.7036200 -0.0464880

C -0.0012606 -1.4136531 0.0264774

C 1.2258367 -0.7035899 0.0728311

C 2.4498735 -1.4107692 0.1457164

C 3.6736847 -0.6996933 0.1920056

C 3.6749612 0.6996338 0.1658077

C -3.6736920 0.6996292 -0.1919993

C -3.6749836 -0.6996413 -0.1658134

C -2.4524202 -1.4107359 -0.0928764

C 4.9116874 1.4276636 0.2126344

C 4.8957776 2.7989336 0.1862468

C -4.8906596 2.7989485 -0.2910803

C -4.9090654 1.4276964 -0.2661132

C -2.4516102 -2.8211800 -0.0664046

C -1.2314557 -3.5265000 0.0062456

C -0.0026153 -2.8167790 0.0527610

C 1.2250240 -3.5265342 0.1258389

C 2.4464325 -2.8211842 0.1720618

C -1.2313555 -4.9367802 0.0326490

C -0.0051586 -5.6455808 0.1056720

C 1.2223599 -4.9367626 0.1522028

C -0.0064357 -7.0559236 0.1321307

C 1.2328105 -7.7393529 0.2055147

C 2.4146113 -7.0642730 0.2504995

C 2.4531249 -5.6439350 0.2256563

C -2.4634369 -5.6439279 -0.0142611

C -2.4275073 -7.0642964 0.0141225

C -1.2469529 -7.7393530 0.0844100

C 3.6526818 -4.9280304 0.2707483

C 3.6795768 -3.5371182 0.2458092

C -3.6860301 -3.5371134 -0.1133101

C -3.6616902 -4.9280324 -0.0861588

C 4.8906285 -2.7989372 0.2910856

C 4.9090855 -1.4276937 0.2661231

C -4.9116949 -1.4276895 -0.2126351

C -4.8957579 -2.7989156 -0.1862484

C 6.1331076 -0.6735473 0.3118940

C 6.1343742 0.6735801 0.2866366

C -6.1331436 0.6736397 -0.3118964

C -6.1343532 -0.6735793 -0.2866332

H 7.0701237 1.2215798 0.3223169

H 7.0679168 -1.2213858 0.3681048

H 5.8282842 -3.3432307 0.3472588

H 4.5896895 -5.4732687 0.3268734

H 3.3518905 -7.6070488 0.3065010

H 1.2261674 -8.8240195 0.2255898

H -1.2423000 -8.8240309 0.1050292

H -3.3658067 -7.6070639 -0.0214469

H -4.5997092 -5.4732888 -0.1218510

H -5.8344072 -3.3432558 -0.2220383

H -7.0701429 -1.2214123 -0.3223188

H -7.0678864 1.2215548 -0.3681105

H -5.8282879 3.3433562 -0.3472726

H -4.5897087 5.4733075 -0.3268962

H -3.3519273 7.6070100 -0.3065168

H -1.2261066 8.8240017 -0.2255927

H 1.2422388 8.8240145 -0.1050374

H 3.3658424 7.6070379 0.0214517

H 4.5997176 5.4733289 0.1218596

H 5.8343926 3.3433628 0.2220440

C -0.8330890 -1.0127083 3.7194535

C 0.5940459 -0.9336510 4.2464696

C 1.2804502 0.3515378 3.8012577

C 0.4719400 1.5828128 4.1916751

C -0.9565658 1.5014604 3.6682049

C -1.6435309 0.2161759 4.1126795

H 2.2891095 0.4101018 4.2216716

H 0.5755258 -0.9713330 5.3433761

H 1.1688337 -1.8035934 3.9128775

H -0.8057506 -1.0867170 2.6269414

H -1.3222925 -1.9232839 4.0790736

H 0.4502451 1.6668201 5.2860065

H 0.9596879 2.4887052 3.8177254

H -1.5319577 2.3732202 3.9951562

H -0.9380731 1.5358563 2.5733181

H -1.7671120 0.2322902 5.2032599

H -2.6490751 0.1581501 3.6841295

H 1.3994723 0.3332861 2.7124688

**25**

C -2.4145829 7.0642840 -0.2504966

C -2.4530977 5.6439310 -0.2256318

C -1.2223392 4.9367242 -0.1521745

C 0.0051579 5.6455466 -0.1056570

C 0.0064359 7.0558788 -0.1321203

C -1.2328199 7.7393022 -0.2055109

C 1.2313787 4.9367003 -0.0326416

C 2.4634174 5.6439573 0.0142562

C 2.4274908 7.0643013 -0.0141219

C 1.2469491 7.7393159 -0.0844082

C -1.2250196 3.5264963 -0.1258101

C 0.0025253 2.8166639 -0.0527674

C 1.2314804 3.5265307 -0.0062618

C 2.4515956 2.8211523 0.0663837

C 3.6860306 3.5370925 0.1132960

C 3.6617124 4.9280612 0.0861554

C -3.6526957 4.9280558 -0.2707400

C -3.6795473 3.5370934 -0.2457796

C -2.4464427 2.8211739 -0.1720357

C -2.4498365 1.4106976 -0.1457027

C -1.2259887 0.7035814 -0.0728565

C 0.0013792 1.4137069 -0.0264984

C 1.2271683 0.7035278 0.0464582

C 2.4524715 1.4106924 0.0928548

C -1.2271728 -0.7036200 -0.0464880

C -0.0012606 -1.4136531 0.0264774

C 1.2258367 -0.7035899 0.0728311

C 2.4498735 -1.4107692 0.1457164

C 3.6736847 -0.6996933 0.1920056

C 3.6749612 0.6996338 0.1658077

C -3.6736920 0.6996292 -0.1919993

C -3.6749836 -0.6996413 -0.1658134

C -2.4524202 -1.4107359 -0.0928764

C 4.9116874 1.4276636 0.2126344

C 4.8957776 2.7989336 0.1862468

C -4.8906596 2.7989485 -0.2910803

C -4.9090654 1.4276964 -0.2661132

C -2.4516102 -2.8211800 -0.0664046

C -1.2314557 -3.5265000 0.0062456

C -0.0026153 -2.8167790 0.0527610

C 1.2250240 -3.5265342 0.1258389

C 2.4464325 -2.8211842 0.1720618

C -1.2313555 -4.9367802 0.0326490

C -0.0051586 -5.6455808 0.1056720

C 1.2223599 -4.9367626 0.1522028

C -0.0064357 -7.0559236 0.1321307

C 1.2328105 -7.7393529 0.2055147

C 2.4146113 -7.0642730 0.2504995

C 2.4531249 -5.6439350 0.2256563

C -2.4634369 -5.6439279 -0.0142611

C -2.4275073 -7.0642964 0.0141225

C -1.2469529 -7.7393530 0.0844100

C 3.6526818 -4.9280304 0.2707483

C 3.6795768 -3.5371182 0.2458092

C -3.6860301 -3.5371134 -0.1133101

C -3.6616902 -4.9280324 -0.0861588

C 4.8906285 -2.7989372 0.2910856

C 4.9090855 -1.4276937 0.2661231

C -4.9116949 -1.4276895 -0.2126351

C -4.8957579 -2.7989156 -0.1862484

C 6.1331076 -0.6735473 0.3118940

C 6.1343742 0.6735801 0.2866366

C -6.1331436 0.6736397 -0.3118964

C -6.1343532 -0.6735793 -0.2866332

H 7.0701237 1.2215798 0.3223169

H 7.0679168 -1.2213858 0.3681048

H 5.8282842 -3.3432307 0.3472588

H 4.5896895 -5.4732687 0.3268734

H 3.3518905 -7.6070488 0.3065010

H 1.2261674 -8.8240195 0.2255898

H -1.2423000 -8.8240309 0.1050292

H -3.3658067 -7.6070639 -0.0214469

H -4.5997092 -5.4732888 -0.1218510

H -5.8344072 -3.3432558 -0.2220383

H -7.0701429 -1.2214123 -0.3223188

H -7.0678864 1.2215548 -0.3681105

H -5.8282879 3.3433562 -0.3472726

H -4.5897087 5.4733075 -0.3268962

H -3.3519273 7.6070100 -0.3065168

H -1.2261066 8.8240017 -0.2255927

H 1.2422388 8.8240145 -0.1050374

H 3.3658424 7.6070379 0.0214517

H 4.5997176 5.4733289 0.1218596

H 5.8343926 3.3433628 0.2220440

C -0.2461541 1.5173650 3.8262152

H -1.1971485 1.9620102 4.1316087

H 0.5604823 2.1321457 4.2343366

H -0.1882779 1.5774797 2.7353171

C -0.1331491 0.0715781 4.2900144

H -0.1950041 0.0614164 5.3860868

C 1.2090844 -0.5245899 3.8889358

H 1.3064711 -1.5579161 4.2324098

H 1.3182552 -0.5271562 2.8007883

H 2.0419140 0.0495942 4.3036496

C -1.2824128 -0.7659152 3.7465224

H -1.2209663 -1.8009563 4.0925919

H -2.2513525 -0.3631441 4.0533121

H -1.2623248 -0.7818815 2.6535168

**26**

C -2.4145829 7.0642840 -0.2504966

C -2.4530977 5.6439310 -0.2256318

C -1.2223392 4.9367242 -0.1521745

C 0.0051579 5.6455466 -0.1056570

C 0.0064359 7.0558788 -0.1321203

C -1.2328199 7.7393022 -0.2055109

C 1.2313787 4.9367003 -0.0326416

C 2.4634174 5.6439573 0.0142562

C 2.4274908 7.0643013 -0.0141219

C 1.2469491 7.7393159 -0.0844082

C -1.2250196 3.5264963 -0.1258101

C 0.0025253 2.8166639 -0.0527674

C 1.2314804 3.5265307 -0.0062618

C 2.4515956 2.8211523 0.0663837

C 3.6860306 3.5370925 0.1132960

C 3.6617124 4.9280612 0.0861554

C -3.6526957 4.9280558 -0.2707400

C -3.6795473 3.5370934 -0.2457796

C -2.4464427 2.8211739 -0.1720357

C -2.4498365 1.4106976 -0.1457027

C -1.2259887 0.7035814 -0.0728565

C 0.0013792 1.4137069 -0.0264984

C 1.2271683 0.7035278 0.0464582

C 2.4524715 1.4106924 0.0928548

C -1.2271728 -0.7036200 -0.0464880

C -0.0012606 -1.4136531 0.0264774

C 1.2258367 -0.7035899 0.0728311

C 2.4498735 -1.4107692 0.1457164

C 3.6736847 -0.6996933 0.1920056

C 3.6749612 0.6996338 0.1658077

C -3.6736920 0.6996292 -0.1919993

C -3.6749836 -0.6996413 -0.1658134

C -2.4524202 -1.4107359 -0.0928764

C 4.9116874 1.4276636 0.2126344

C 4.8957776 2.7989336 0.1862468

C -4.8906596 2.7989485 -0.2910803

C -4.9090654 1.4276964 -0.2661132

C -2.4516102 -2.8211800 -0.0664046

C -1.2314557 -3.5265000 0.0062456

C -0.0026153 -2.8167790 0.0527610

C 1.2250240 -3.5265342 0.1258389

C 2.4464325 -2.8211842 0.1720618

C -1.2313555 -4.9367802 0.0326490

C -0.0051586 -5.6455808 0.1056720

C 1.2223599 -4.9367626 0.1522028

C -0.0064357 -7.0559236 0.1321307

C 1.2328105 -7.7393529 0.2055147

C 2.4146113 -7.0642730 0.2504995

C 2.4531249 -5.6439350 0.2256563

C -2.4634369 -5.6439279 -0.0142611

C -2.4275073 -7.0642964 0.0141225

C -1.2469529 -7.7393530 0.0844100

C 3.6526818 -4.9280304 0.2707483

C 3.6795768 -3.5371182 0.2458092

C -3.6860301 -3.5371134 -0.1133101

C -3.6616902 -4.9280324 -0.0861588

C 4.8906285 -2.7989372 0.2910856

C 4.9090855 -1.4276937 0.2661231

C -4.9116949 -1.4276895 -0.2126351

C -4.8957579 -2.7989156 -0.1862484

C 6.1331076 -0.6735473 0.3118940

C 6.1343742 0.6735801 0.2866366

C -6.1331436 0.6736397 -0.3118964

C -6.1343532 -0.6735793 -0.2866332

H 7.0701237 1.2215798 0.3223169

H 7.0679168 -1.2213858 0.3681048

H 5.8282842 -3.3432307 0.3472588

H 4.5896895 -5.4732687 0.3268734

H 3.3518905 -7.6070488 0.3065010

H 1.2261674 -8.8240195 0.2255898

H -1.2423000 -8.8240309 0.1050292

H -3.3658067 -7.6070639 -0.0214469

H -4.5997092 -5.4732888 -0.1218510

H -5.8344072 -3.3432558 -0.2220383

H -7.0701429 -1.2214123 -0.3223188

H -7.0678864 1.2215548 -0.3681105

H -5.8282879 3.3433562 -0.3472726

H -4.5897087 5.4733075 -0.3268962

H -3.3519273 7.6070100 -0.3065168

H -1.2261066 8.8240017 -0.2255927

H 1.2422388 8.8240145 -0.1050374

H 3.3658424 7.6070379 0.0214517

H 4.5997176 5.4733289 0.1218596

H 5.8343926 3.3433628 0.2220440

C -0.8962207 2.2657440 3.7713343

H -1.6851231 2.8571219 4.2428400

H 0.0605649 2.6648190 4.1106092

H -0.9632015 2.4303409 2.6909222

C -1.0627765 0.7851574 4.0992994

H -0.9282422 0.6564211 5.1834428

C -0.0039959 -0.0798833 3.3974648

H -0.1527572 0.0666847 2.3230717

C -2.4801383 0.3501331 3.7398840

H -2.7034591 -0.6651610 4.0699366

H -3.2158997 1.0152257 4.1989328

H -2.6316476 0.3909532 2.6562750

C -0.1735608 -1.5668291 3.6862935

H -1.1229720 -1.9559561 3.3150613

H 0.6217617 -2.1430911 3.2064093

H -0.1239888 -1.7642715 4.7625034

C 1.4211631 0.3480844 3.7258909

H 1.6359155 1.3662147 3.3977820

H 1.6072642 0.2932487 4.8037069

H 2.1404227 -0.3095859 3.2309353

**26 (TPSS)**

C -2.4145829 7.0642840 -0.2504966

C -2.4530977 5.6439310 -0.2256318

C -1.2223392 4.9367242 -0.1521745

C 0.0051579 5.6455466 -0.1056570

C 0.0064359 7.0558788 -0.1321203

C -1.2328199 7.7393022 -0.2055109

C 1.2313787 4.9367003 -0.0326416

C 2.4634174 5.6439573 0.0142562

C 2.4274908 7.0643013 -0.0141219

C 1.2469491 7.7393159 -0.0844082

C -1.2250196 3.5264963 -0.1258101

C 0.0025253 2.8166639 -0.0527674

C 1.2314804 3.5265307 -0.0062618

C 2.4515956 2.8211523 0.0663837

C 3.6860306 3.5370925 0.1132960

C 3.6617124 4.9280612 0.0861554

C -3.6526957 4.9280558 -0.2707400

C -3.6795473 3.5370934 -0.2457796

C -2.4464427 2.8211739 -0.1720357

C -2.4498365 1.4106976 -0.1457027

C -1.2259887 0.7035814 -0.0728565

C 0.0013792 1.4137069 -0.0264984

C 1.2271683 0.7035278 0.0464582

C 2.4524715 1.4106924 0.0928548

C -1.2271728 -0.7036200 -0.0464880

C -0.0012606 -1.4136531 0.0264774

C 1.2258367 -0.7035899 0.0728311

C 2.4498735 -1.4107692 0.1457164

C 3.6736847 -0.6996933 0.1920056

C 3.6749612 0.6996338 0.1658077

C -3.6736920 0.6996292 -0.1919993

C -3.6749836 -0.6996413 -0.1658134

C -2.4524202 -1.4107359 -0.0928764

C 4.9116874 1.4276636 0.2126344

C 4.8957776 2.7989336 0.1862468

C -4.8906596 2.7989485 -0.2910803

C -4.9090654 1.4276964 -0.2661132

C -2.4516102 -2.8211800 -0.0664046

C -1.2314557 -3.5265000 0.0062456

C -0.0026153 -2.8167790 0.0527610

C 1.2250240 -3.5265342 0.1258389

C 2.4464325 -2.8211842 0.1720618

C -1.2313555 -4.9367802 0.0326490

C -0.0051586 -5.6455808 0.1056720

C 1.2223599 -4.9367626 0.1522028

C -0.0064357 -7.0559236 0.1321307

C 1.2328105 -7.7393529 0.2055147

C 2.4146113 -7.0642730 0.2504995

C 2.4531249 -5.6439350 0.2256563

C -2.4634369 -5.6439279 -0.0142611

C -2.4275073 -7.0642964 0.0141225

C -1.2469529 -7.7393530 0.0844100

C 3.6526818 -4.9280304 0.2707483

C 3.6795768 -3.5371182 0.2458092

C -3.6860301 -3.5371134 -0.1133101

C -3.6616902 -4.9280324 -0.0861588

C 4.8906285 -2.7989372 0.2910856

C 4.9090855 -1.4276937 0.2661231

C -4.9116949 -1.4276895 -0.2126351

C -4.8957579 -2.7989156 -0.1862484

C 6.1331076 -0.6735473 0.3118940

C 6.1343742 0.6735801 0.2866366

C -6.1331436 0.6736397 -0.3118964

C -6.1343532 -0.6735793 -0.2866332

H 7.0701237 1.2215798 0.3223169

H 7.0679168 -1.2213858 0.3681048

H 5.8282842 -3.3432307 0.3472588

H 4.5896895 -5.4732687 0.3268734

H 3.3518905 -7.6070488 0.3065010

H 1.2261674 -8.8240195 0.2255898

H -1.2423000 -8.8240309 0.1050292

H -3.3658067 -7.6070639 -0.0214469

H -4.5997092 -5.4732888 -0.1218510

H -5.8344072 -3.3432558 -0.2220383

H -7.0701429 -1.2214123 -0.3223188

H -7.0678864 1.2215548 -0.3681105

H -5.8282879 3.3433562 -0.3472726

H -4.5897087 5.4733075 -0.3268962

H -3.3519273 7.6070100 -0.3065168

H -1.2261066 8.8240017 -0.2255927

H 1.2422388 8.8240145 -0.1050374

H 3.3658424 7.6070379 0.0214517

H 4.5997176 5.4733289 0.1218596

H 5.8343926 3.3433628 0.2220440

C -0.8991078 2.2811426 3.7695142

H -1.6908102 2.8731760 4.2426155

H 0.0613449 2.6774536 4.1111961

H -0.9647705 2.4424795 2.6865267

C -1.0672121 0.7884563 4.1034905

H -0.9343296 0.6612867 5.1898301

C 0.0005340 -0.0828524 3.3944568

H -0.1481368 0.0644364 2.3181095

C -2.4958565 0.3485480 3.7383454

H -2.7148222 -0.6703655 4.0701117

H -3.2330757 1.0149972 4.2003764

H -2.6448833 0.3898076 2.6526837

C -0.1702494 -1.5829823 3.6844826

H -1.1227052 -1.9684248 3.3092330

H 0.6281787 -2.1578369 3.2018973

H -0.1224639 -1.7810847 4.7632017

C 1.4375907 0.3496803 3.7262983

H 1.6475737 1.3709803 3.3957672

H 1.6229659 0.2957936 4.8068419

H 2.1574801 -0.3094451 3.2283128

**26 (CAM-B3LYP)**

C -2.4145829 7.0642840 -0.2504966

C -2.4530977 5.6439310 -0.2256318

C -1.2223392 4.9367242 -0.1521745

C 0.0051579 5.6455466 -0.1056570

C 0.0064359 7.0558788 -0.1321203

C -1.2328199 7.7393022 -0.2055109

C 1.2313787 4.9367003 -0.0326416

C 2.4634174 5.6439573 0.0142562

C 2.4274908 7.0643013 -0.0141219

C 1.2469491 7.7393159 -0.0844082

C -1.2250196 3.5264963 -0.1258101

C 0.0025253 2.8166639 -0.0527674

C 1.2314804 3.5265307 -0.0062618

C 2.4515956 2.8211523 0.0663837

C 3.6860306 3.5370925 0.1132960

C 3.6617124 4.9280612 0.0861554

C -3.6526957 4.9280558 -0.2707400

C -3.6795473 3.5370934 -0.2457796

C -2.4464427 2.8211739 -0.1720357

C -2.4498365 1.4106976 -0.1457027

C -1.2259887 0.7035814 -0.0728565

C 0.0013792 1.4137069 -0.0264984

C 1.2271683 0.7035278 0.0464582

C 2.4524715 1.4106924 0.0928548

C -1.2271728 -0.7036200 -0.0464880

C -0.0012606 -1.4136531 0.0264774

C 1.2258367 -0.7035899 0.0728311

C 2.4498735 -1.4107692 0.1457164

C 3.6736847 -0.6996933 0.1920056

C 3.6749612 0.6996338 0.1658077

C -3.6736920 0.6996292 -0.1919993

C -3.6749836 -0.6996413 -0.1658134

C -2.4524202 -1.4107359 -0.0928764

C 4.9116874 1.4276636 0.2126344

C 4.8957776 2.7989336 0.1862468

C -4.8906596 2.7989485 -0.2910803

C -4.9090654 1.4276964 -0.2661132

C -2.4516102 -2.8211800 -0.0664046

C -1.2314557 -3.5265000 0.0062456

C -0.0026153 -2.8167790 0.0527610

C 1.2250240 -3.5265342 0.1258389

C 2.4464325 -2.8211842 0.1720618

C -1.2313555 -4.9367802 0.0326490

C -0.0051586 -5.6455808 0.1056720

C 1.2223599 -4.9367626 0.1522028

C -0.0064357 -7.0559236 0.1321307

C 1.2328105 -7.7393529 0.2055147

C 2.4146113 -7.0642730 0.2504995

C 2.4531249 -5.6439350 0.2256563

C -2.4634369 -5.6439279 -0.0142611

C -2.4275073 -7.0642964 0.0141225

C -1.2469529 -7.7393530 0.0844100

C 3.6526818 -4.9280304 0.2707483

C 3.6795768 -3.5371182 0.2458092

C -3.6860301 -3.5371134 -0.1133101

C -3.6616902 -4.9280324 -0.0861588

C 4.8906285 -2.7989372 0.2910856

C 4.9090855 -1.4276937 0.2661231

C -4.9116949 -1.4276895 -0.2126351

C -4.8957579 -2.7989156 -0.1862484

C 6.1331076 -0.6735473 0.3118940

C 6.1343742 0.6735801 0.2866366

C -6.1331436 0.6736397 -0.3118964

C -6.1343532 -0.6735793 -0.2866332

H 7.0701237 1.2215798 0.3223169

H 7.0679168 -1.2213858 0.3681048

H 5.8282842 -3.3432307 0.3472588

H 4.5896895 -5.4732687 0.3268734

H 3.3518905 -7.6070488 0.3065010

H 1.2261674 -8.8240195 0.2255898

H -1.2423000 -8.8240309 0.1050292

H -3.3658067 -7.6070639 -0.0214469

H -4.5997092 -5.4732888 -0.1218510

H -5.8344072 -3.3432558 -0.2220383

H -7.0701429 -1.2214123 -0.3223188

H -7.0678864 1.2215548 -0.3681105

H -5.8282879 3.3433562 -0.3472726

H -4.5897087 5.4733075 -0.3268962

H -3.3519273 7.6070100 -0.3065168

H -1.2261066 8.8240017 -0.2255927

H 1.2422388 8.8240145 -0.1050374

H 3.3658424 7.6070379 0.0214517

H 4.5997176 5.4733289 0.1218596

H 5.8343926 3.3433628 0.2220440

C -0.8965323 2.2670580 3.7711207

H -1.6840485 2.8568104 4.2419877

H 0.0581861 2.6649157 4.1101675

H -0.9628512 2.4301607 2.6927581

C -1.0626676 0.7849727 4.0998649

H -0.9286071 0.6567343 5.1812187

C -0.0040461 -0.0797941 3.3962759

H -0.1521151 0.0660094 2.3263995

C -2.4815488 0.3499263 3.7395763

H -2.7037236 -0.6631238 4.0695865

H -3.2155290 1.0138628 4.1980869

H -2.6315848 0.3901221 2.6578781

C -0.1732512 -1.5681735 3.6855336

H -1.1208733 -1.9561522 3.3154843

H 0.6206618 -2.1430306 3.2067138

H -0.1242375 -1.7635436 4.7600164

C 1.4225813 0.3481101 3.7252948

H 1.6364229 1.3641390 3.3981146

H 1.6065488 0.2935870 4.8014461

H 2.1402755 -0.3084046 3.2313900

**27**

C -2.4145829 7.0642840 -0.2504966

C -2.4530977 5.6439310 -0.2256318

C -1.2223392 4.9367242 -0.1521745

C 0.0051579 5.6455466 -0.1056570

C 0.0064359 7.0558788 -0.1321203

C -1.2328199 7.7393022 -0.2055109

C 1.2313787 4.9367003 -0.0326416

C 2.4634174 5.6439573 0.0142562

C 2.4274908 7.0643013 -0.0141219

C 1.2469491 7.7393159 -0.0844082

C -1.2250196 3.5264963 -0.1258101

C 0.0025253 2.8166639 -0.0527674

C 1.2314804 3.5265307 -0.0062618

C 2.4515956 2.8211523 0.0663837

C 3.6860306 3.5370925 0.1132960

C 3.6617124 4.9280612 0.0861554

C -3.6526957 4.9280558 -0.2707400

C -3.6795473 3.5370934 -0.2457796

C -2.4464427 2.8211739 -0.1720357

C -2.4498365 1.4106976 -0.1457027

C -1.2259887 0.7035814 -0.0728565

C 0.0013792 1.4137069 -0.0264984

C 1.2271683 0.7035278 0.0464582

C 2.4524715 1.4106924 0.0928548

C -1.2271728 -0.7036200 -0.0464880

C -0.0012606 -1.4136531 0.0264774

C 1.2258367 -0.7035899 0.0728311

C 2.4498735 -1.4107692 0.1457164

C 3.6736847 -0.6996933 0.1920056

C 3.6749612 0.6996338 0.1658077

C -3.6736920 0.6996292 -0.1919993

C -3.6749836 -0.6996413 -0.1658134

C -2.4524202 -1.4107359 -0.0928764

C 4.9116874 1.4276636 0.2126344

C 4.8957776 2.7989336 0.1862468

C -4.8906596 2.7989485 -0.2910803

C -4.9090654 1.4276964 -0.2661132

C -2.4516102 -2.8211800 -0.0664046

C -1.2314557 -3.5265000 0.0062456

C -0.0026153 -2.8167790 0.0527610

C 1.2250240 -3.5265342 0.1258389

C 2.4464325 -2.8211842 0.1720618

C -1.2313555 -4.9367802 0.0326490

C -0.0051586 -5.6455808 0.1056720

C 1.2223599 -4.9367626 0.1522028

C -0.0064357 -7.0559236 0.1321307

C 1.2328105 -7.7393529 0.2055147

C 2.4146113 -7.0642730 0.2504995

C 2.4531249 -5.6439350 0.2256563

C -2.4634369 -5.6439279 -0.0142611

C -2.4275073 -7.0642964 0.0141225

C -1.2469529 -7.7393530 0.0844100

C 3.6526818 -4.9280304 0.2707483

C 3.6795768 -3.5371182 0.2458092

C -3.6860301 -3.5371134 -0.1133101

C -3.6616902 -4.9280324 -0.0861588

C 4.8906285 -2.7989372 0.2910856

C 4.9090855 -1.4276937 0.2661231

C -4.9116949 -1.4276895 -0.2126351

C -4.8957579 -2.7989156 -0.1862484

C 6.1331076 -0.6735473 0.3118940

C 6.1343742 0.6735801 0.2866366

C -6.1331436 0.6736397 -0.3118964

C -6.1343532 -0.6735793 -0.2866332

H 7.0701237 1.2215798 0.3223169

H 7.0679168 -1.2213858 0.3681048

H 5.8282842 -3.3432307 0.3472588

H 4.5896895 -5.4732687 0.3268734

H 3.3518905 -7.6070488 0.3065010

H 1.2261674 -8.8240195 0.2255898

H -1.2423000 -8.8240309 0.1050292

H -3.3658067 -7.6070639 -0.0214469

H -4.5997092 -5.4732888 -0.1218510

H -5.8344072 -3.3432558 -0.2220383

H -7.0701429 -1.2214123 -0.3223188

H -7.0678864 1.2215548 -0.3681105

H -5.8282879 3.3433562 -0.3472726

H -4.5897087 5.4733075 -0.3268962

H -3.3519273 7.6070100 -0.3065168

H -1.2261066 8.8240017 -0.2255927

H 1.2422388 8.8240145 -0.1050374

H 3.3658424 7.6070379 0.0214517

H 4.5997176 5.4733289 0.1218596

H 5.8343926 3.3433628 0.2220440

C -1.8334830 1.7406498 4.2928329

H -2.8060937 1.9496395 4.7459455

H -1.0772672 2.2413440 4.8985597

H -1.8312489 2.1983774 3.2978760

C -1.6125793 0.2349012 4.1954388

H -1.5985749 -0.1674554 5.2192860

C -0.2629808 -0.1557035 3.5555078

H -0.2710435 0.2286275 2.5270307

C -2.8052289 -0.3755240 3.4662373

H -2.8037574 -1.4656085 3.4808562

H -3.7416916 -0.0433308 3.9215381

H -2.8197181 -0.0541418 2.4214058

C -0.1421700 -1.6737521 3.4646768

H -0.9319425 -2.0959321 2.8436070

H 0.8036344 -1.9791021 3.0165847

H -0.2115449 -2.1393146 4.4531212

C 0.9355633 0.5142755 4.2850607

H 0.6083449 0.7308769 5.3111147

C 1.3374010 1.8293556 3.6205502

H 0.4919674 2.4893341 3.4298581

H 2.0602452 2.3748071 4.2335383

H 1.8139319 1.6268397 2.6582378

C 2.1859384 -0.3529865 4.3965408

H 2.0086973 -1.2826073 4.9396306

H 2.5808700 -0.6092913 3.4082655

H 2.9699048 0.1956096 4.9256734

**28**

C -2.4145829 7.0642840 -0.2504966

C -2.4530977 5.6439310 -0.2256318

C -1.2223392 4.9367242 -0.1521745

C 0.0051579 5.6455466 -0.1056570

C 0.0064359 7.0558788 -0.1321203

C -1.2328199 7.7393022 -0.2055109

C 1.2313787 4.9367003 -0.0326416

C 2.4634174 5.6439573 0.0142562

C 2.4274908 7.0643013 -0.0141219

C 1.2469491 7.7393159 -0.0844082

C -1.2250196 3.5264963 -0.1258101

C 0.0025253 2.8166639 -0.0527674

C 1.2314804 3.5265307 -0.0062618

C 2.4515956 2.8211523 0.0663837

C 3.6860306 3.5370925 0.1132960

C 3.6617124 4.9280612 0.0861554

C -3.6526957 4.9280558 -0.2707400

C -3.6795473 3.5370934 -0.2457796

C -2.4464427 2.8211739 -0.1720357

C -2.4498365 1.4106976 -0.1457027

C -1.2259887 0.7035814 -0.0728565

C 0.0013792 1.4137069 -0.0264984

C 1.2271683 0.7035278 0.0464582

C 2.4524715 1.4106924 0.0928548

C -1.2271728 -0.7036200 -0.0464880

C -0.0012606 -1.4136531 0.0264774

C 1.2258367 -0.7035899 0.0728311

C 2.4498735 -1.4107692 0.1457164

C 3.6736847 -0.6996933 0.1920056

C 3.6749612 0.6996338 0.1658077

C -3.6736920 0.6996292 -0.1919993

C -3.6749836 -0.6996413 -0.1658134

C -2.4524202 -1.4107359 -0.0928764

C 4.9116874 1.4276636 0.2126344

C 4.8957776 2.7989336 0.1862468

C -4.8906596 2.7989485 -0.2910803

C -4.9090654 1.4276964 -0.2661132

C -2.4516102 -2.8211800 -0.0664046

C -1.2314557 -3.5265000 0.0062456

C -0.0026153 -2.8167790 0.0527610

C 1.2250240 -3.5265342 0.1258389

C 2.4464325 -2.8211842 0.1720618

C -1.2313555 -4.9367802 0.0326490

C -0.0051586 -5.6455808 0.1056720

C 1.2223599 -4.9367626 0.1522028

C -0.0064357 -7.0559236 0.1321307

C 1.2328105 -7.7393529 0.2055147

C 2.4146113 -7.0642730 0.2504995

C 2.4531249 -5.6439350 0.2256563

C -2.4634369 -5.6439279 -0.0142611

C -2.4275073 -7.0642964 0.0141225

C -1.2469529 -7.7393530 0.0844100

C 3.6526818 -4.9280304 0.2707483

C 3.6795768 -3.5371182 0.2458092

C -3.6860301 -3.5371134 -0.1133101

C -3.6616902 -4.9280324 -0.0861588

C 4.8906285 -2.7989372 0.2910856

C 4.9090855 -1.4276937 0.2661231

C -4.9116949 -1.4276895 -0.2126351

C -4.8957579 -2.7989156 -0.1862484

C 6.1331076 -0.6735473 0.3118940

C 6.1343742 0.6735801 0.2866366

C -6.1331436 0.6736397 -0.3118964

C -6.1343532 -0.6735793 -0.2866332

H 7.0701237 1.2215798 0.3223169

H 7.0679168 -1.2213858 0.3681048

H 5.8282842 -3.3432307 0.3472588

H 4.5896895 -5.4732687 0.3268734

H 3.3518905 -7.6070488 0.3065010

H 1.2261674 -8.8240195 0.2255898

H -1.2423000 -8.8240309 0.1050292

H -3.3658067 -7.6070639 -0.0214469

H -4.5997092 -5.4732888 -0.1218510

H -5.8344072 -3.3432558 -0.2220383

H -7.0701429 -1.2214123 -0.3223188

H -7.0678864 1.2215548 -0.3681105

H -5.8282879 3.3433562 -0.3472726

H -4.5897087 5.4733075 -0.3268962

H -3.3519273 7.6070100 -0.3065168

H -1.2261066 8.8240017 -0.2255927

H 1.2422388 8.8240145 -0.1050374

H 3.3658424 7.6070379 0.0214517

H 4.5997176 5.4733289 0.1218596

H 5.8343926 3.3433628 0.2220440

C -1.7119150 2.0479847 4.4166051

H -2.6116290 2.3894333 4.9353403

H -0.8519204 2.4122513 4.9799163

H -1.7018665 2.5272975 3.4320615

C -1.7343089 0.5294556 4.2793656

H -1.7560296 0.1033332 5.2933856

C -0.4874797 -0.0653519 3.5878892

H -0.4579496 0.3555497 2.5741194

C -3.0277731 0.1328816 3.5731082

H -3.2071104 -0.9423243 3.5866756

H -3.8862248 0.6151281 4.0478105

H -3.0080293 0.4557662 2.5281852

C -0.6801574 -1.5711288 3.4078681

H -1.4275277 -1.7649782 2.6379653

H 0.2310202 -2.0760292 3.0890114

H -1.0330021 -2.0520594 4.3241769

C 0.8351210 0.4034498 4.2783044

H 0.5821303 0.7040747 5.3054276

C 1.3619336 1.6368895 3.5442034

H 0.5623002 2.3300071 3.2882071

H 2.0990434 2.1861430 4.1322527

H 1.8389636 1.3406641 2.6080624

C 1.9419790 -0.6646859 4.4173453

H 1.9968869 -1.2236324 3.4747713

C 3.3284864 -0.0692604 4.6515829

H 4.0512969 -0.8703992 4.8274457

H 3.6863031 0.5132982 3.8018044

H 3.3350133 0.5780262 5.5346759

C 1.6467846 -1.6348509 5.5572587

H 0.6562578 -2.0806712 5.4916556

H 2.3781440 -2.4473928 5.5810338

H 1.7079742 -1.1072625 6.5148861
